# A [6+4]-cycloaddition adduct is the biosynthetic intermediate in streptoseomycin biosynthesis

**DOI:** 10.1038/s41467-021-22395-7

**Published:** 2021-04-07

**Authors:** Kai Biao Wang, Wen Wang, Bo Zhang, Xin Wang, Yu Chen, Hong Jie Zhu, Yong Liang, Ren Xiang Tan, Hui Ming Ge

**Affiliations:** 1 State Key Laboratory of Pharmaceutical Biotechnology, Institute of Functional Biomolecules, Chemistry and Biomedicine Innovation Center (ChemBIC), School of Life Sciences, Nanjing University, Nanjing, China; 2 State Key Laboratory of Coordination Chemistry, Jiangsu Key Laboratory of Advanced Organic Materials, Chemistry and Biomedicine Innovation Center, School of Chemistry and Chemical Engineering, Nanjing University, Nanjing, China

**Keywords:** Oxidoreductases, Biocatalysis, Applied microbiology, Biosynthesis

## Abstract

Streptoseomycin (STM, **1**) is a bacterial macrolactone that has a unique 5/14/10/6/6-pentacyclic ring with an ether bridge. We have previously identified the biosynthetic gene cluster for **1** and characterized StmD as [6 + 4]- and [4 + 2]-bispericyclase that catalyze a reaction leading to both 6/10/6- and 10/6/6-tricyclic adducts (**6** and **7**). The remaining steps, especially how to install and stabilize the required 10/6/6-tricyclic core for downstream modifications, remain unknown. In this work, we have identified three oxidoreductases that fix the required 10/6/6-tryciclic core. A pair of flavin-dependent oxidoreductases, StmO1 and StmO2, catalyze the direct hydroxylation at [6 + 4]-adduct (**6**). Subsequently, a spontaneous [3,3]-Cope rearrangement and an enol-ketone tautomerization result in the formation of 10/6/6-tricyclic intermediate **12b**, which can be further converted to a stable 10/6/6-tricyclic alcohol **11** through a ketoreduction by StmK. Crystal structure of the heterodimeric complex NtfO1-NtfO2, homologues of StmO1-StmO2 with equivalent function, reveals protein-protein interactions. Our results demonstrate that the [6 + 4]-adduct instead of [4 + 2]-adduct is the bona fide biosynthetic intermediate.

## Abstract (web summary)

Streptoseomycin is a potent antibiotic that contains a pentacyclic 5/14/10/6/6 ring system. Here, the authors report the enzymatic and non-enzymatic steps of the downstream modification of streptoseomycin biosynthesis and show a [6 + 4]-cycloaddition adduct as an unexpected biosynthetic intermediate.

## Introduction

**Fig. 1:**
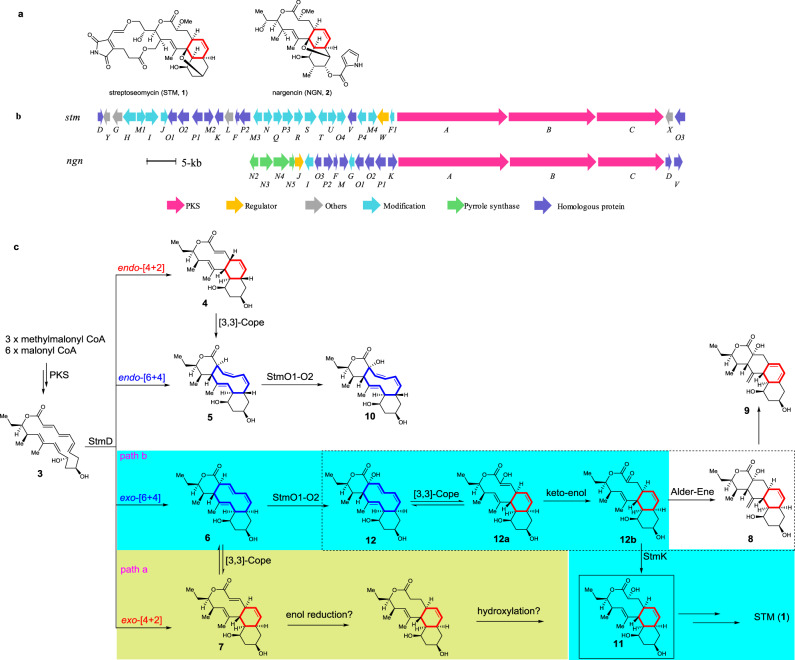
Biosynthesis of streptoseomycin. **a** Structures of streptoseomycin (**1**) and nargenicin (**2**); **b** Genetic organization of the *stm* and *ngn* gene clusters; **c** biosynthetic pathway involved in polyketide part of STM biosynthesis. Path a (yellow background) is the original pathway using [4 + 2]-adduct as the intermediate. Path b (blue background) is the revised pathway starting from [6 + 4]-adduct. Steps in the dashed region are nonenzymatic processes.

Streptoseomycin (STM, **1**) is a unique bacterial macrolactone produced from a marine bacterium *Streptomyces seoulensis* A01^1^. STM is structurally similar to nargenicin (NGN, **2**) (Fig. 1a)^2^, nodusmicin^3^, coloradocin^4,5^, as well as branimycin^6^, all of which have a common 10(9)/6/6-tricyclic core. They are potent antibiotics against array of anaerobic or microaerophilic bacteria including the human pathogenic bacterium *Helicobacter pylori*, representing a class of promising antibiotics^1–8^. Previously, we have cloned and sequenced the biosynthetic gene clusters for **1** and **2** producers^1,9^. The *stm* and *ngn* gene clusters are highly homologues in sequence (Fig. 1b and Supplementary Table 3). According to the domain organization of three giant polyketide synthases (PKSs)^10^, we proposed an 18-membered macrolactone intermediate **3** was the PKS product in **1** biosynthesis (Fig. 1)^9^. This hypothesis was supported by our recent biosynthetic study that a linear polyketide, a spontaneous hydrolyzed product of **3**, was obtained from a recombinant strain where all tailoring enzymes were eliminated from the *stm* gene cluster^9^. In addition, we have characterized StmD as the bispericyclase that catalyzes the formation of [6 + 4] and [4 + 2] adducts, both of which can interconvert to each other through a spontaneous [3,3]-Cope rearrangement (Fig. 1), in the biosynthesis of **1** through detailed in vivo gene deletion, in vitro biochemical assay as well as density functional theory (DFT) calculations^9^. However, the following questions remain unanswered: (i) which adduct, [6 + 4] or [4 + 2], is the real biosynthetic intermediate for downstream enzymes, (ii) at which step the [6 + 4]-adduct can fully convert to the [4 + 2]-adduct for remaining steps, and (iii) when the [4 + 2]-adduct is stable.

As **7** shares the same 10/6/6-tricyclic ring system with **1**, we proposed [4 + 2]-adduct **7** instead of [6 + 4]-adduct **6** is the real intermediate in **1** biosynthesis, and the thermodynamically more stable [6 + 4] adduct **6** is converted into the [4 + 2] adduct **7** through Cope rearrangement when a downstream enzyme, putatively an enoyl reductase-like enzyme is present to transform the C=C double bond in the α,β-unsaturated ester group of **7** to the C–C single bond and stabilize the ten membered ring (Fig. 1c, path a)^11,12^. To identify the downstream enzymes, we comparatively analyzed the encoded enzymes in *stm* and *ngn* gene cluster (Fig. 1b, Supplementary Table 3). Totally, 13 homologous enzymes were identified, five of which have already been experimentally characterized by us and other lab: StmA/NgnA, StmB/NgnB, and StmC/NgnC as three giant PKSs^1^, StmD/NgnD as a bispericyclase^9^, and StmO3/NgnO3 as a dioxygenase that catalyzes the ether-bridge formation^13,14^. In addition, StmF/NgnF, StmM/NgnM, and StmV/NgnV, which were predicted to be ferredoxin, methyltransferase and thioesterase, respectively, were unlikely to directly utilize **6** or **7** as substrates. The remaining uncharacterized homologous enzymes in **1** and **2** biosyntheses include two P450 monooxygenases, StmP1/NgnP1 and StmP2/NgnP2, as well as three NAD(P)H or flavin-dependent oxidoreductases StmO1/NgnO1, StmO2/NgnO2 and StmK/NgnK.

In this work, we report an unusual combination of enzymatic and nonenzymatic cascade installs the 10/6/6-tricyclic core in **1** biosynthesis by the following steps: (i) a pair of flavin-dependent hydroxylases, StmO1–StmO2 complex, hydroxylate C2 in **6** generating a hydroxyl group, (ii) a subsequent spontaneous [3,3]-Cope rearrangement followed by an enol-ketone tautomerization forms the 10/6/6-tricyclic core with a C2 ketone group, and (iii) an NADPH-dependent ketoreductase, StmK, reduces the C2 ketone group affording a stable 10/6/6-tricyclic core; alternatively, ketone-containing intermediate can undergo a nonenzymatic Alder-ene rearrangement forming a 6/6/6/6-tetracyclic shunt metabolite. Moreover, X-ray structure analysis of a pair of StmO1-StmO2 homologous enzymes, NtfO1–NtfO2, reveals protein–protein interactions.

## Results and discussion

### StmO1, StmO2, and StmK are essential for STM biosynthesis

**Fig. 2:**
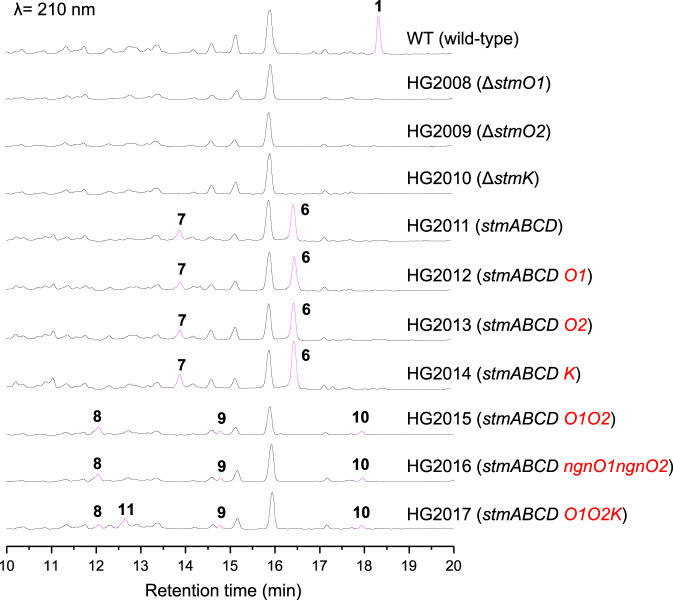
HPLC analysis of wild-type and recombinant strains. The highlighted peaks were intermediates or shunt metabolites in the *stm* pathway.

As we proposed above, an enoyl reductase-like enzyme was likely required to reduce the C=C double bond in the α,β-unsaturated ester group of **7** using NAD(P)H or reduced flavin as cofactor (Fig. 1c, path a)^1,9^. Thus, we turned our attention on three oxidoreductases, StmO1, StmO2, and StmK. We individually inactivated these three genes through in-frame deletion in *S. seoulensis* A01 wild-type strain, generating HG2008 (Δ*stmO1*), HG2009 (Δ*stmO2*), and HG2010 (Δ*stmK*) mutant strains, respectively (Supplementary Fig. 1). As expected, all these mutants abolished the production of **1** under the fermentation condition described previously^9^, confirming the involvement of these three genes in STM biosynthesis. However, it is strange that no new intermediates including **6** and **7** can be detected from these mutants (Fig. 2)^14^. The production of **1** can be restored when gene *stmO1*, *stmO2,* or *stmK* was individually complemented to their corresponding mutants (Supplementary Fig. 2), excluding the possible polar effects of each gene mutation.

### StmO1-StmO2 carries out a hydroxylation step

To exclude the possible effects of other tailoring enzymes encoded in the *stm* gene cluster, we carried out a ‘bottom-up’ strategy to reconstitute the biosynthetic pathway step by step. First, we removed two remaining putative tailoring enzymes encoding genes *stmX* and *stmO3* in the mutant strain HG2006, where a DNA fragment from *stmY* to *stmF1* were deleted^9^, yielding HG2011 (*stmABCD*) strain that only harbors three *pks* genes (*stmA*, *stmB* and *stmC*) and a bispericyclase gene (*stmD*) in the *stm* gene cluster. Under the fermentation condition, HG2011 lost the production of **1** but clearly accumulated *exo*-[6 + 4]-adduct (**6**) and *exo-*[4 + 2]-adduct (**7**) as well as a minor *endo*-[6 + 4]-adduct (**5**) (Fig. 2), which was barely detectable. Our previous study has confirmed that **6** and **7** were biosynthetic intermediates for **1**, while **5** is an off-pathway metabolite. In addition, in vivo experiments and DFT calculations indicated that **5** was formed exclusively in *endo-*pathway and the proposed [4 + 2] adduct **4** does not exist due to its high energy^9^.

Using HG2011 strain as a platform, we set out to evaluate if StmO1, StmO2 or StmK can directly accept and tailor **6** or **7**. Therefore, we cloned these genes individually to the *Streptomyces* integrative plasmid pSET152 under the control of a strong promoter *kasO*p*^15^, and introduced them into HG2011 strain, yielding HG2012 (*stmABCDO1*), HG2013 (*stmABCDO2*), and HG2014 (*stmABCDK*), respectively. However, HPLC analysis indicated none of these recombinant strains could generate any new products (Fig. 2). The partially overlapped coding regions of *stmO1* and *stmO2* suggest that they are located in the same operon. It is most likely that StmO1 and StmO2 also exist in a complex form. Thus, we cloned *stmO1* and *stmO2* genes together to pSET152 plasmid under the control of *kasO*p* promoter and transformed into HG2011, affording HG2015 (*stmABCDO1O2*) strain. After cultivation, LC-MS analysis of the organic extract showed the appearance of three new peaks, **8** (*m/z* 385.2 [M + Na]^+^), **9** (*m/z* 383.2 [M + Na]^+^) and **10** (*m/z* 385.2 [M + Na]^+^) (Fig. 2). A similar HPLC profile was observed when we co-introduced *ngnO1* and *ngnO2* genes into HG2011 strain (Fig. 2), suggesting StmO1–StmO2 are functionally equivalent to NgnO1–NgnO2. **8**–**10** were isolated and their structures were elucidated by NMR analysis (Experimental procedures and Supplementary Tables 5–7). While **10** contains an additional hydroxyl group at C-2 compared with **5**, both **8** and **9** have an unexpected 6/6/6/6-tetracyclic ring system with an exocyclic alkene (Fig. 1). To test if one of these compounds (**8**–**10**) is an on-pathway biosynthetic intermediate, we separately fed **8**, **9**, and **10** to Δ*stmA* mutant, a *pks* deletion strain^9^. LC-MS analysis of metabolic extracts revealed that none of them could restore the production of **1** (Supplementary Fig. 3), indicating **8**–**10** are not biosynthetic intermediates. Nonetheless, structure analyses for **8**–**10** imply that (i) StmO1–O2 catalyze an oxidation reaction, (ii) **10** could be the direct hydroxylated product of **5**, (iii) **8** could be a spontaneous rearranged result from a real StmO1–O2 enzymatic product as the stereochemistry of cyclohexene ring in **8** is consistent with that of **7**, and (iv) **9** might be an overoxidized product of **8**.

### In vivo characterization of StmK as a reductase

Due to our inability to obtain any biosynthetic intermediates from HG2015 strain, we reasoned that the introduction of one more downstream gene into HG2015 strain might catch and convert the labile intermediate to a more stable product. As we analyzed above that the reaction catalyzed by StmO1–StmO2 results in an increase in the oxidation state, a reduction step was still required to generate a proposed intermediate **11** from **6** or **7**. For this purpose, the only remaining NAD(P)H-dependent oxidoreductase encoding gene *stmK* was cloned into the replicative expression vector pUWL201, placing it under the control of a strong promoter *ermE*p*^15^. The resulting construct was conjugated into HG2015 to afford HG2017 (*stmABCDO1O2K*)^16,17^. LC-MS analysis showed that HG2017 strain could alter the HPLC profile and generate an additional peak (*m/z* 387.2 [M + Na]^+^) (Fig. 2). A large-scale fermentation of HG2017 strain led to the isolation of 3.6 mg of **11**. Its structure was then determined by analysis of its HRESIMS and NMR data (Experimental procedures, and Supplementary Table 8). Gratifyingly, the structure of **11** is consistent with the proposed biosynthetic intermediate (Fig. 1). Chemical complementation of **11** into Δ*stmA* mutant strain restored the production of **1** (Supplementary Fig. 3), confirming **11** is an on-pathway intermediate. Therefore, three redox enzymes, StmO1, StmO2 and StmK, are sufficient to yield the stable 10/6/6-tricyclic intermediate in **1** biosynthesis.

With compounds **8**–**11** in hand, we re-analyze the LC-MS data of the above generated mutant strains. The extracted ion chromatography (EIC) revealed the presence of **5**–**7** in Δ*stmO1* and Δ*stmO2* mutant strains (Supplementary Fig. 4), albeit in low liters; while, ion peaks corresponding to **8**–**10** were observed in Δ*stmK* strain. These observations are in agreement with functional assignments for StmO1, StmO2, and StmK. The reason that no significant products were generated from mutant strains are probably due to other genes in the gene cluster or the genome may also somehow act on the intermediates. Alternatively, these steps are the key bottleneck steps in the biosynthesis of **1**^14^. Placing these genes under strong promoters in our ‘bottom-up’ strategy may boost the metabolic flux, resulting in the increased production of **8**–**10**.

### In vitro characterization of NgnO1-NgnO2 as hydroxylase

**Fig. 3:**
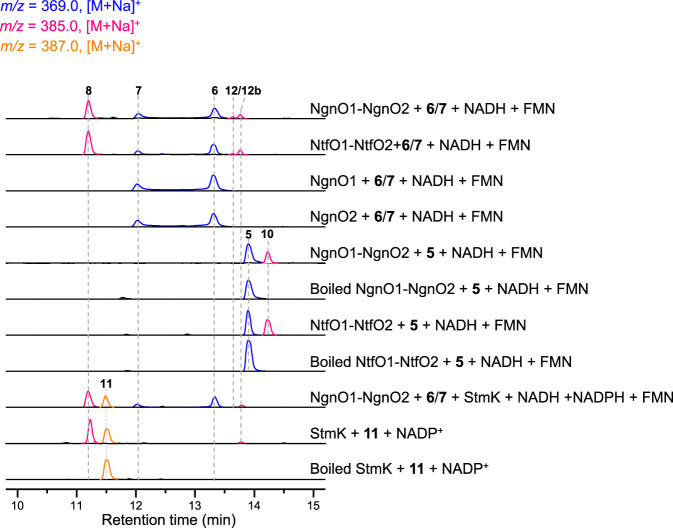
LC-MS analysis of NgnO1-NgnO2, StmK, and NtfO1–NtfO2 catalyzed reaction. NgnO1-NgnO2 catalyzes a hydroxylation step on 6/7 or 5 when incubated with FMN and NADH; NgnO1 or NgnO2 alone has no effects on 6/7; NtfO1-NtfO2 has the same function with NgnO1-NgnO2; StmK catalyzes a ketoreduction step on the putative intermediate **12b**; StmK can catalyze a reverse reaction using **11** as substrate and NADP^+^ as a cofactor.

Given that our in vivo data did not give a clear insight into the conversion from **6**/**7** to **11**, we turned to in vitro reconstitution. We cloned and overexpressed *stmO1* and *stmO2* in *E. coli* BL21(DE3). StmO1 was soluble, while StmO2 was not soluble on its own or when co-expressed with StmO1. After several attempts, we successfully obtained their homologous enzymes NgnO1 and NgnO2 as soluble proteins (Supplementary Fig. 5)^18^. Incubation of NgnO1 and NgnO2 with the substrate mixture of **6**/**7** in the presence of cofactors NADH and FMN led to the production of **8** (Fig. 3 and Supplementary Fig. 6b). The requirement of cofactors (NADH, NADPH, FAD, and FMN) were systematically investigated. No product can be detected when either NAD(P)H or flavin was omitted from the reaction mixture. NgnO1–NgnO2 prefers to utilize NADH and FMN as a cofactor combination (Supplementary Fig. 7).

Considering that **5** and **6** have the same planar structure, we then tested if NgnO1-NgnO2 can also accept **5**. The NgnO1-NgnO2 assay of **5** under the same reaction condition resulted in the production of **10** (Fig. 3 and Supplementary Fig. 6d), clearly indicating that NgnO1–NgnO2 could directly hydroxylate the *sp*^*3*^-hybrized carbon C2 of **5**. This result strongly suggested NgnO1–NgnO2 utilized **6** instead of **7** as substrate, and **12** is the real product. Similar to the interconversion between **6** and **7**^9^, **12** can convert to **12a** through a spontaneous [3,3]-Cope rearrangement followed by an enol-ketone tautomerization forms the 10/6/6-tricyclic compound **12b**^7,19,20^. The enol-ketone tautomerization is the driving force for the Cope rearrangement from **12** to **12a**. Because we only observed **8** instead of **12b** from in vivo and in vitro experiments, therefore a spontaneous Alder-ene reaction may take place and convert **12b** to **8** (Fig. 1)^21,22^. The selection of one component in equilibrium with another for biosynthesis is reminiscent of the biosynthesis of phosphonate from PEP, where a homocitrate synthase, FrbC, provides the thermodynamic driving force needed to pull the unfavorable PEP mutase reaction^23^.

### DFT calculation for the proposed pathway

**Fig. 4:**
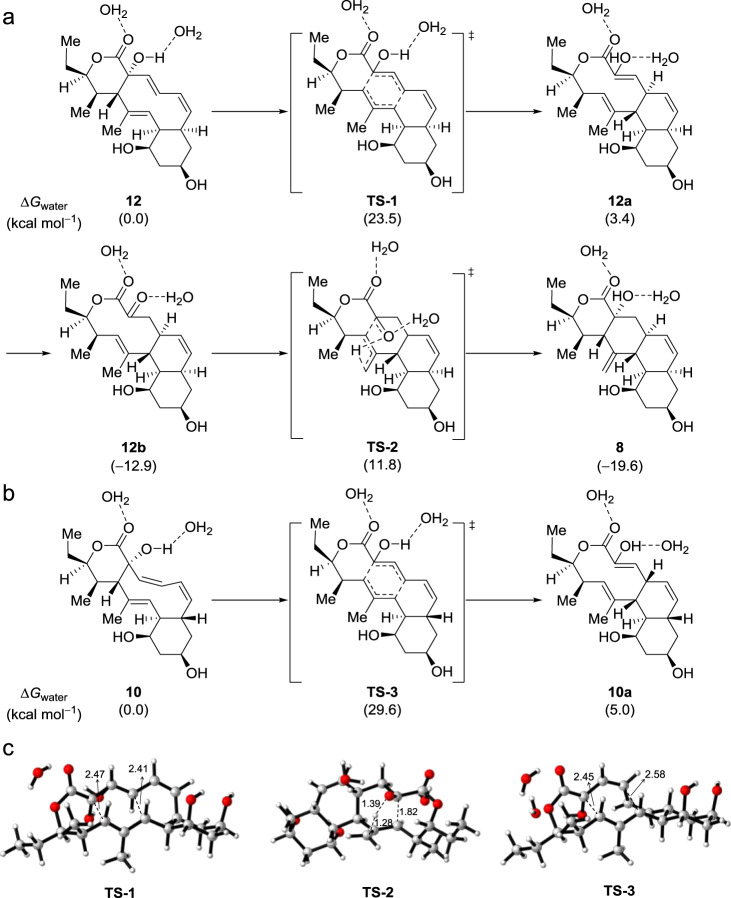
DFT-computed Gibbs free energies. **a** the Cope rearrangement of **12** and the subsequent Alder-ene reaction; **b** the Cope rearrangement of **10**; **c** transition-state structures (Carbon: gray, Hydrogen: white, Oxygen: red, and distances are shown in Å).

To validate the above hypotheses, we performed DFT calculations (Supplementary Data 1). As shown in Fig. 4a, the [3,3]-Cope rearrangement of the predicted C2-hydroxylated intermediate **12** requires an activation free energy of 23.5 kcal mol^−1^ in water. Such a barrier means that kinetically, this transformation can smoothly occur at room temperature. The generated 10/6/6/-tricyclic enol **12a** is slightly energonic by 3.4 kcal mol^-1^, but the subsequent tautomerization from enol **12a** to ketone **12b** dramatically stabilizes the 10/6/6-tricyclic core, since this process is highly exergonic by 16.3 kcal mol^−1^. Kinetically, the enol-ketone tautomerization can easily occur at room temperature in the presence of water^24^. Further calculations show that the trisubstituted alkene and ketone moieties within the 10-membered ring of **12b** undergo an Alder-ene reaction through transition state **TS-2**, in which the forming C–C and O–H bonds are 1.82 and 1.39 Å, respectively, and the breaking C–H bond is 1.28 Å (Fig. 4c). The computed free energy barrier for this transannular Alder-ene reaction is 24.7 kcal mol^−1^, just a bit higher than that for the Cope rearrangement of **12**, and the resulting 6/6/6/6-tetracyclic product **8** is exergonic by 6.7 kcal mol^−1^. All these computational results support that the conversion of C2-hydroxylated compound **12** to the observed product **8** can spontaneously take place in water via Cope rearrangement, enol-ketone tautomerization, and Alder-ene reaction. In addition, DFT calculations showed that for the isolated C2-hydroxylated product **10**, its [3,3]-Cope rearrangement transition state **TS-3** (Fig. 4b) has an activation barrier of 29.6 kcal mol^−1^, which is about 6 kcal mol^−1^ higher than that for intermediate **12** (**TS-3**: 29.6 kcal mol^−1^ versus **TS-1**: 23.5 kcal mol^−1^). This indicates that compound **10** is stable enough in the water at ambient temperature, which is in agreement with the experimental findings.

### In vitro characterization of StmK as a ketoreductase

We reasoned that StmK may account for the ketoreduction step of **12b** to yield **11**. StmK was then overproduced and purified from *E. coli* BL21(DE3) to homogeneity. Because the proposed substrate **12b** is not available, we performed a StmK and NgnO1–NgnO2 coupled enzymatic reaction. Upon incubating StmK and NADH into the above NgnO1–NgnO2 reaction system, the production of **11**, together with the shunt metabolite **8**, was observed through LC-MS analysis (Fig. 3).

To further confirm the function of StmK, we carried out a StmK catalyzed reverse reaction using **11** as substrate. The purified recombinant StmK was incubated with **11** in the presence of excessive NADP^+^, which led to the formation of **8** along with a small ion peak ([M + Na]^+^ at 385.1990) corresponding to C_21_H_30_O_5_ (Supplementary Fig. 6g), which most likely is **12b**. Further analysis of EIC spectrum of NgnO1–NgnO2 catalyzed reaction indicated the presence of two minor peaks that could be assigned to **12** and **12b** according to their high-resolution mass data (Fig. 3 and Supplementary Fig. 6b). Though these two compounds cannot be obtained in sufficient amount for structure elucidation, the time-course analysis of NgnO1–NgnO2 biochemical reaction showed the production of these peaks increased at an early time point, and finally converted to the end product (Supplementary Fig. 8), suggesting both compounds are biosynthetic intermediates (Fig. 1c). The DFT calculations indicated **12** and **12b** may have a reasonably long half-life for LC-MS detection (Fig. 4). Collectively, these results support the proposed biosynthetic pathway and confirm StmK is a ketoreductase responsible for reducing C2-keto group of **12b** to afford **11**.

### Complex structure of NtfO1–NtfO2

**Fig. 5:**
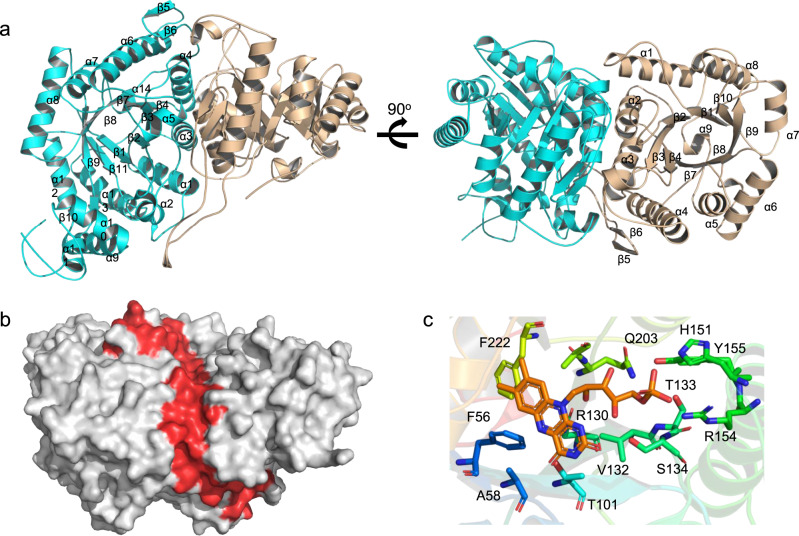
Crystal structure of NtfO1–NtfO2. **a** the cartoon structure of NtfO1 (wheat) and NtfO2 (cyan) complex; **b** the surface of O1/O2 complex and the interface was colored red (for more detail see Supplementary Fig. 11); **c** Key residues of NtfO2, which bind the cofactor, FMN.

Finally, we set out to characterize the structure of NgnO1–NgnO2 for understanding the presumed protein–protein interaction. A highly similar gene cluster (*ntf*) of *stm* or *ngn* was identified from *Nocardia tenerifensis* NBRC 101015 strain (Supplementary Table 4)^9^. Most likely, this gene cluster encodes a nargenicin-like compound. Indeed, the recombinant NtfO1–NtfO2 is functionally equivalent to NgnO1–NgnO2, as they also catalyze the same reaction on **5** or **6**/**7** (Fig. 3). Though we failed to get the crystals of NgnO1–NgnO2, the complex of NtfO1–NtfO2 was successfully crystallized in the P12_1_1 space group as a heterodimer in the asymmetric unit with resolution at 2.0 Å (Fig. 5, Supplementary Table 9 and PDB: 7C2R). Consistent with crystal analysis, the quaternary state of NtfO1–NtfO2 complex in solution was determined as a heterodimer by size-exclusion chromatographic analysis (Supplementary Fig. 5). The flat interface area is around 15, 120 Å^2^, covering 21.6% of the total surface. The interface is mainly composed of two sets of α-helices, α2/α3 from NtfO1 and α3/α4 from NtfO2 (Fig. 5 and Supplementary Fig. 10). Besides polar interactions between NtfO1 and NtfO2, like hydrogen bonding network among NtfO2/D32, NtfO1/R156, NtfO2/E78, and NtfO1/Y163, the predominant interactions are contributed by the hydrophobic residues (Supplementary Fig. 10). Although StmO1, NgnO1, and NtfO1 share relatively low sequence identity in contrast to StmO2, NgnO2, and NtfO2, amino acid residues located in the interface are highly conserved (Supplementary Figs. 10−12), suggesting StmO1–StmO2 and NgnO1–NgnO2, should also form similar heterodimers.

The overall structures of NtfO1 and NtfO2 adopted a typical TIM-barrel with five additional insertions and C-terminus extension to block the barrel, which is a typical structure of luciferase-like monooxygenase family (Supplementary Fig. 13)^25–29^. While NtfO2 possesses the conserved lid over the C-terminal end of the β-barrel, it lacked a highly conserved side loop tail (Supplementary Fig. 13). Conversely, NtfO1 has the side loop tail and misses the lid (Supplementary Fig. 13). Since the side loop tail is quite conserved and contributes extensive interaction between each subunit in the luciferase-like monooxygenase family, the complementary structure feature of NtfO1 and NtfO2 might be the reason why they forge a heterodimer.

In addition, the FMN was docking into the NtfO2 with −8.2 kcal/mol affinity by using structurally similar luciferase-like monooxygenases (1YW1, 5XKD, 3B9O, 6LR5) as templates^25,26^. The phosphate end of FMN was hydrogen bonded to several hydrophilic residues including Q203, Y155, H151, and T133, and the isoalloxazine ring of FMN was laying on the top of the TIM barrel core and surrounded by F222, F56, A58, and T101. To evaluate the putative interaction between key residues and FMN, we carried out site-directed mutagenesis on NtfO2. The activity of these mutated proteins were analyzed by measuring the formation of **8** by LC-MS analysis. The results showed that H151A, T133A, T101A, and F56A almost completely lost activities compared with wild type (Supplementary Fig. 14). The activities of Y155A and Q203A were decreased by around 80 and 90%, and F222A mutant remains ~40% activity.

In conclusion, we have identified three oxidoreductases StmO1–O2 and StmK that act in tandem to construct the 10/6/6-tricyclic skeleton required for STM biosynthesis (Fig. 1, path b). Notably, StmO1–StmO2 hydroxylate [6 + 4]-adduct **6** instead of [4 + 2]-adduct **7** to **12**, which is unexpected. The proposed intermediate **12** undergoes a reversible, nonenzymatic Cope rearrangement to give **12a**. To completely shift the equilibrium toward **12a**, the reaction has exploited driving force by a subsequent irreversible, nonenzymatic enol-ketone tautomerization. Without the downstream enzyme StmK, **12b** can be further converted to **8** through a spontaneous Alder-ene reaction. However, with StmK, the off-pathway reaction can be suppressed in favor of a ketoreduction on the C2-carbonyl group. Our work therefore demonstrates an interesting example of how nature creates a combination of enzymatic and nonenzymatic reactions, which are precisely controlled, to synthesize natural products.

## Methods

### General experimental procedures

All 1D and 2D NMR experiments were run on a Bruker Avance 600 with a cryoprobe at 600 MHz for ^1^H and 150 MHz for ^13^C nuclei or a Bruker Avance III 400 at 400 MHz for ^1^H and 100 MHz for ^13^C nuclei. High-resolution LC-MS analysis was performed on an Agilent 6530 TOF LC-MS spectrometer equipped with a Porshell 120 EC-C18 column (4.5 × 50 mm, 2.7 μm, Agilent Technologies). Optical rotation values were measured in methanol on a Rudolph Autopol IV automatic polarimeter. UV spectra were recorded on a Nanodrop 2000 spectrometer (Thermo Scientific, USA) with a 10 mm cuvette. IR spectra were measured on a Nexus870 FT-IR. Semipreparative RP-HPLC was performed on an Agilent 1260 HPLC system with a DAD detector equipped with an Eclipse XDB-C18 column (C-18, 9.4 × 250 mm, 5 µm, Agilent Technologies). MPLC fractionation was performed on a Biotage Isolera One using a Biotage SNAP Cartridge C18 column (120 g). Recombinant proteins were purified on a GE ÄKTA pure chromatography system with a 5 mL Histrap HP column (GE Lifesciences).

### Bacterial strains, plasmids, culture conditions, and chemicals

The strains and plasmids used in this study are listed in Supplementary Table 1. PCR primers were synthesized by Genscript Biological Technology Co. and listed in Supplementary Table 2. The restriction endonucleases and Phanta Max Super-Fidelity DNA Polymerase were obtained from Thermo Fisher and Vazyme Biotech, respectively. DNA gel extraction and plasmid preparation kits were purchased from Omega Bio-Tek. *E. coli* strains harboring plasmids were grown in LB (Luria-Bertani medium) with appropriate antibiotics, such as ampicillin (100 μg/mL), apramycin (50 μg/mL), kanamycin (50 μg/mL) or chloramphenicol (25 μg/mL)^30^. *E. coli* ET12567/pUZ8002 was used as the host for intergeneric conjugation to *S. seoulensis* A01 and mutant strains were cultivated on ISP4 medium at 28 °C for sporulation. Biological reagents, chemicals, media, and enzymes were purchased from standard commercial sources unless otherwise indicated.

### Gene inactivation and complementation

Double crossover homologous recombination was used for gene (*stmX-stmO3, stmO1*, *stmO2,* and *stmK*) disruption. To construct the plasmid for inactivation of the *stmX-stmO3* gene, the upstream and downstream homologous arms were amplified with up-F/R primers and down-F/R primers (Supplementary Table 2) using genomic DNA of *S. seoulensis* A01 as a template. Both arms were cloned into the *Hind*III and *EcoR*I sites of pKC1139 to afford plasmid pHG02034. pHG02034 was then conjugated into *S. seoulensis* A01 following the standard procedure^31^. After cultured for 3 days at 30 °C, the colonies with apramycin resistant were transferred to ISP4 plates supplied with apramycin antibiotics at a final concentration of 50 μg/mL. The apramycin-sensitive colonies were picked as candidate for double-crossover mutants. The genotype of Δ*stmX-stmO3* was verified by diagnostic PCR using the primers listed in Supplementary Table 2 and DNA-gel analysis (Supplementary Fig. 1). Inactivation of *stmO1*, *stmO2,* and *stmK* were performed using the same method described above.

For gene complementation, the fragment containing *stmO1* has ligated to the *Spe*I and *EcoR*I sites of pSET152-*KasO*p* plasmid to produce pHG02038. Then, the plasmid pHG02038 was transformed into HG2008 (∆*stmO1*) by conjugation to generate strain HG2018, and transformed to HG2011 (*stmABCD*) to obtain strain HG2012 (*stmABCDO1*).

Similarly, the pSET152-*KasO*p* derived plasmid pHG02039 that contained the gene *stmO2* was conjugated to HG2009 (Δ*stmO2*) and HG2011 (*stmABCD*) to afford HG2019 (Δ*stmO2::stmO2*) and HG2013 (*stmABCDO2*), respectively. The pSET152-*KasO*p* derived plasmid pHG02040 that contained the gene *stmK* was conjugated to HG2010 (Δ*stmK*) and HG2011 (*stmABCD*) to afford HG2020 (Δ*stmK::stmK*) and HG2014 (*stmABCDK*), respectively. The pSET152-*KasO*p* derived plasmid pHG02041 containing genes *stmO1* and *stmO2* was transformed to HG2011 (*stmABCD*) to generate strain HG2015 (*stmABCDO1O2*). The plasmid pHG02042 containing the gene *ngnO1* and *ngnO2* was transformed to HG2011 (*stmABCD*) to generate strain HG2016 (*stmABCDngnO1O2*). The pUWL201-*ErmE*p* derived plasmid pHG02043 that contained *stmK* gene was transferred to the strain HG2015 to generate strain HG2017 (*stmABCDO1O2K*).

### Fermentation and analysis of intermediates

The small-scale fermentations for wild-type and gene recombinant strains were carried out using the same method reported previously^1^. In brief, fresh spores of mutant strains were inoculated into 50 mL TSB medium at 28 °C and 200 rpm for two days. The seed culture was then inoculated into a production medium (5 g peptone, 2 g beef extract, 5 g glucose, 1.0 g K_2_HPO_4_, 0.5 g FeSO_4_ in 1 L water, pH 7.0) at 28 °C and cultivated for eight days. Then, the supernatant of the fermentation broth was extracted with ethyl acetate and concentrated for LC-MS analysis. LC-MS analysis was performed in a 23 min linear gradient system from 20 to 90% (v/v) methanol in water containing 0.1% formic acid.

### Isolation of the intermediates

For compound isolation, a 10-L scale fermentation for (HG2015 and HG2017) was carried out individually. The fermentation broth was filtered and the supernatant was extracted three times with ethyl acetate, then the crude extract was separated by a medium-pressure liquid chromatography (MPLC) system equipped a Biotage SNAP Ultra C18 120 g column using a linear gradient system from 20 to 90% (v/v) methanol/water. The fractions were analyzed by HPLC. Those fractions containing the target compounds were combined and further purified by semi-preparative HPLC. Compound **8** (8.3 mg, t_*R*_ = 21.5 min) was purified from the HG2015 strain by semi-preparative HPLC using 25% MeCN in H_2_O at a flow rate of 2 mL/min. Compound **9** (7.5 mg, *t*_*R*_ = 18.6 min) and compound **10** (1.5 mg, *t*_*R*_ = 25.3 min) were purified from the same strain by semi-preparative HPLC using 25% MeCN in H_2_O and 21% MeCN in H_2_O, respectively, at a flow rate of 2 mL/min. Compound **11** (3.6 mg, *t*_*R*_ = 27.1 min) was purified from the HG2017 strain by semi-preparative HPLC using 34% MeCN in H_2_O at a flow rate of 2 mL/min.

### Structure elucidation for 8–11

The molecular formula of compound **8** was determined to be C_21_H_30_O_5_ based on its HR-ESIMS analysis (Supplementary Fig. 15) and NMR data (Supplementary Table 5, Supplementary Figs. 16–22), indicating seven degrees of unsaturation. The ^1^H NMR, ^13^C NMR, and HSQC spectra displayed the presence of one ester carbonyl (C-1), one trisubstituted olefinic carbon (C-14), one terminal olefinic carbon (C-21), two olefinic methines (C-5, C-6), accounting for three out of seven degrees of unsaturation. Therefore, **8** should be a tetracyclic structure. In addition, NMR data revealed the presence of three oxygenated aliphatic methines (C-9, C-11, and C-17), six aliphatic methines (C-4, C-7, C-12, C-13, C-15, and C-16), four aliphatic methylenes (C-3, C-8, C-10, and C-18), two methyl groups (C-19 and C-20), one oxygenated quaternary carbon (C-2) and three D_2_O exchangeable signals (*δ*_H_ 3.27, 3.69, 4.38) corresponding to three hydroxyl groups. Interpretation of the ^1^H-^1^H COSY spectrum revealed the presence of two distinctive spin systems, H-3 to H-13, and H-15 to H-20. The two spin systems were connected through HMBC and ^13^C chemical shift analysis. The presence of one decalin subunit was indicated by ^1^H-^1^H COSY and HMBC corrections from H-3, H-5 to C-13, and H-6, H-12 to C-4. HMBC corrections from H-15, H-17, and 2-OH to C-1 allowed for the formation of a 6-membered lactone ring. Another 6-membered ring was established from H-21 to C-13, C-14, and C-15, and from 2-OH to C-1, C-2, C-3, and C-15. Thus, the planar structure of **8** was determined. The NOE correlations of 2-OH with H-4, of H-17 with H-6, and of H-20 with H-15 established the stereochemistry of C-2, C-15, C-16, and C-17.

The molecular formula of compound **9** was determined to be C_21_H_28_O_5_ based on the HR-ESIMS analysis (Supplementary Fig. 23) and NMR data (Supplementary Table 6, Supplementary Figs. 24–30), suggesting that **9** has one more degree of unsaturation than that of **8**. The NMR data of **9** were highly similar to those of **8**, except for the absence of signals corresponding to aliphatic methine at C-4 and C-7 in **8**. Instead, there were two additional sp^2^-hybridized carbons corresponding to trisubstituted olefinic carbons (*δ*_C_ 133.6 and 131.7). The above observation suggested that one of the single bond in **8** might be desaturated in **9**, which was in agreement with the molecular mass of **9**. HMBC correlations from H-12 to C-4, from H-6 to C-4 and C-12, and from H-5 to C-7 and C-13 revealed the presence of a cyclohexadiene ring. Further interpretation of 1D and 2D NMR data established the structure of **9**.

The molecular formula of compound **10** was determined to be C_21_H_30_O_5_ according to its HR-ESIMS (Supplementary Fig. 31) and NMR data (Supplementary Table 7, Supplementary Figs. 32–38), indicating 7 degrees of unsaturation. The NMR data of **10** were very similar to those of **5**^9^, except for the absence of signals corresponding to aliphatic methine at C-2 in **5**. Instead, there were additional NMR signals corresponding to an oxygenated quaternary carbon (*δ*_C_ 79.4) and one hydroxyl proton (2-OH, *δ*_H_ 4.92, brs). The HMBC correlations from 2-OH to C-2 and C-3 indicated **10** has an additional 2-hydroxyl group at C-2 position compared with **5**. Compound **10** showed a similar NOE pattern to that of **5**^9^. In addition, the NOE correlation of 2-OH with H-16 established the stereochemistry of C-2 as shown in Fig. 1.

The molecular formula of compound **11** was determined to be C_21_H_32_O_5_ based on the HR-ESIMS analysis (Supplementary Fig. 39) and NMR data (Supplementary Table 8, Supplementary Figs. 40–46), indicating six degrees of unsaturation. The ^1^H NMR, ^13^C NMR, and HSQC spectra indicated the presence of one ester carbonyl (C-1), three olefinic methines (C-5, C-6, and C-15), one trisubstituted olefinic carbon (C-14), accounting for three out of six degrees of unsaturation. Therefore, **11** is a tricyclic structure. Analysis 1D and 2D NMR data indicated the presence of four oxygenated aliphatic methines (C-2, C-9, C-11, and C-17), six aliphatic methines (C-4, C-7, C-12, C-13, and C-16), four aliphatic methylenes (C-3, C-8, C-10, and C-18), three methyl groups (C-19, C-20, and C-21). Interpretation of the ^1^H-^1^H COSY spectrum displayed the presence of two distinctive spin systems, H-2 to H-13, and H-15 to H-20. One methyl group (C-21) was attached to C-14 through HMBC correlations of H-21 to C-13, C-14, and C-15. The presence of one decalin subunit was indicated by ^1^H-^1^H COSY and HMBC corrections from H-3, H-5, and H-7 to C-13 and H-6, H-12 to C-4^9^. In addition, the HMBC correlations from H-2, H-3, H-17, and 2-OH to C-1 allowed for the formation of a 10-membered lactone ring. Thus, the planar structure of **8** was determined. The NOE correlations from H-2 (*δ*_H_ 4.02) to H-3β (*δ*_H_ 1.78), and from H-3β (*δ*_H_ 1.78) to H-5 (*δ*_H_ 5.58) established the stereochemistry of C-2, which was confirmed by our feeding experiment that **11** is a biosynthetic intermediate in streptoseomycin biosynthesis.

### Physicochemical data of 8–11

Compound **8**, yellow oil, [α]_D_^25^ −47.5 (*c* 0.16, MeOH); UV(MeOH): λmax (log *ε*) = 208 (3.25) nm; IR (KBr) *ν*_max_ 3396, 3011, 2968, 2934, 2879, 1725, 1644 cm^−1^; HR-ESIMS (positive mode): *m/z* calcd. for C_21_H_30_O_5_Na^+^: 385.1985, found 385.1991 [M + Na]^+^. ^1^H and ^13^C NMR data see Supplementary Table 5.

Compound **9**, yellow oil, [α]_D_^25^ −177.3 (*c* 0.56, MeOH); UV(MeOH): *λ*max (log *ε*) = 217 (3.27) nm, 270 (3.55) nm, 281 (3.59) nm, 291 (3.41) nm; IR (KBr) *ν*_max_ 3395, 3094, 2967, 2932, 2879, 1730, 1642 cm^−1^; HR-ESIMS (positive mode): *m/z* calcd. for C_21_H_28_O_5_Na^+^: 383.1829, found 383.1832 [M + Na]^+^. ^1^H and ^13^C NMR data see Supplementary Table 6.

Compound **10**, colorless needle, [α]_D_^25^ + 45.3 (*c* 0.07, MeOH); UV(MeOH): *λ*max (log *ε*) = 214 (3.38) nm, 228 (3.34) nm; IR (KBr) ν_max_ 3295, 2924, 2855, 1736, 1710, 1599 cm^−1^; HR-ESIMS (positive mode): *m/z* calcd. for C_21_H_30_O_5_Na^+^: 385.1985, found 385.1993 [M + Na]^+^. ^1^H and ^13^C NMR data see Supplementary Table 7.

Compound **11**, white soild, [α]_D_^25^ -16.0 (*c* 0.05, MeOH); UV(MeOH): *λ*max (log *ε*) = 210 (3.41) nm; IR (KBr) *ν*_max_ 3371, 3013, 2964, 2929, 2876, 1718, 1603 cm^−1^; HR-ESIMS (positive mode): *m/z* calcd. for C_21_H_32_O_5_Na^+^: 387.2142, found 387.2149 [M + Na]^+^. ^1^H and ^13^C NMR data see Supplementary Table 8.

### Gene expression and protein purification

The gene *stmO1* was amplified by PCR using primers 28a-*stmO1*-F/R with genomic DNA of *S. seoulensis* A01 as template. After purification, the fragment was ligated with linearized pET28a(+) (digested by *Hind*III and *Nde*I) using the ClonExpress MultiS One Step Cloning Kit (Vazyme Co., Biotech) according to the manufacturer’s protocol to generate pHG02044. For gene expression of NgnO1, NgnO2, NtfO1–NtfO2, and StmK, similar procedures were carried out, the fragment of *ngnO1*, *ngnO2, ntfO1-ntfO2,* and *stmK* was amplified by PCR using the genomic DNA of *N. argentinensis* ATCC 31306, *N. tenerifensis* NBRC 101015 and *S. seoulensis* A01 as template respectively. After purification, the fragments were ligated with linearized pET22b(+) (digested by *Hind*III and *Nde*I) except the *ngnO1* ligated with linearized pET28a(+), then generate plasmids pHG02045, pHG02046, pHG02047, and pHG02048, respectively.

For gene *stmO1* expression, the plasmid pHG02044 was individually transformed into *E. coli* BL21(DE3), and grown in 400 mL LB at 37 °C with shaking at 220 rpm until an OD_600_ of 0.4–0.6 was reached. The culture was cooled to 4 °C and induced with 0.125 mM IPTG and continued to cultivate at 16 °C and 220 rpm for 18 h. The cells were harvested by centrifugation at 9000 × *g* for 10 min. Then the cells were resuspended in 25 mL lysis buffer (100 mM Tris, pH 8.0, 15 mM imidazole, 300 mM NaCl, 10 % glycerol) and sonicated at 4 °C. Lysated cell was centrifugated at 18,500 × *g* for 30 min at 4 °C. The supernatant was collected and filtered, then purified by ÄKTA FPLC system using a 5 mL Histrap HP column (GE lifesciences). The protein was concentrated with an Amicon Ultra-15 10 kDa spinfilter (EMD Millipore) and desalted by a PD10 column (GE Healthcare) with 100 mM phosphate buffer (pH 7.0) contained 10% glycerol. The protein was frozen in liquid nitrogen and stored at −80 °C.

For gene *ngnO1* expression, the plasmid pHG02045 was introduced into *E. coli* BL21(DE3) and using the similar procedure described in *stmO1* expression, we obtained the recombinant NgnO1.

For gene *ngnO2* expression, the plasmid pHG02046 was introduced into *E. coli* BL21(DE3) containing the molecular chaperone plasmid, pGtf2 (Cat. # 3340; Takara Bio Inc)^18,32,33^. The single transformant was cultivated overnight at 37 °C in 5 mL LB containing ampicillin (100 μg/mL) and chloramphenicol (25 μg/mL). Then, the overnight culture of the recombinant strain was transferred 1% to 400 mL LB medium added 100 μg/mL ampicillin, 25 μg/mL chloramphenicol, and 10 ng/mL tetracyclin (inducing the expression of *groES-groEL-tig*) cultivated at 37 °C and 220 rpm until the OD_600_ value reached 0.6. The culture was cooled to 4 °C and induced with 0.125 mM IPTG and continued to cultivate at 16 °C and 220 rpm for 18 h. The cells were harvested and resuspended in lysis buffer and centrifuged after sonication. The supernatant was filtered and purified by ÄKTA FPLC system equipped with a 5 mL Histrap HP column (GE Lifesciences). And the protein was desalted and stored at −80 °C.

For gene *NtfO1-NtfO2* expression, the plasmid pHG02047 was introduced into *E. coli* BL21(DE3). Using the similar procedure described in *stmO1* expression, the NtfO1–NtfO2 were obtained as heterodimer.

For gene *stmK* expression, the plasmid pHG02048 was introduced into *E. coli* BL21(DE3) containing the chaperone plasmid, pKJE7 (Cat. # 3340; Takara Bio Inc)^18,32,33^. Using the similar procedure described in *ngnO2* expression, we obtained the recombinant StmK.

The concentration of protein was determined from the absorbance at 280 nm using a molar absorptivity constant calculated by the ExPASy/ProtParam tool (https://web.expasy.org/protparam/).

### In vitro assay of NgnO1, NgnO2, NgnO1–NgnO2

The NgnO1, NgnO2 or NgnO1–NgnO2 catalyzed reaction was carried out in a 100 μL reaction system containing 50 mM MES buffer (pH 6.7), 200 μM substrate, 1 mM NADH, 2 μM FMN, 2 μM NgnO1 or 2 μM NgnO2 or 2 μM NgnO1 and 2 μM NgnO2. After incubation at 30 °C for 3 h, 100 μL acetonitrile was added to quench the reaction. Then, the reaction mixture was centrifuged at 18,500 × *g* for 10 min, and the supernatant was subjected to LC-MS analysis. The LC-MS analysis was performed with an 18 min gradient elution system from 10 to 90% (1–13 min), 100% (13–15 min), and 10% (15–18 min) methanol in water supplied with 0.1% formic acid at a flow rate of 0.5 mL/min.

### In vitro assay of NtfO1–NtfO2

The NtfO1–NtfO2 catalyzed reaction was carried out in a 100 μL reaction system containing 50 mM MES buffer (pH 6.7), 200 μM substrate, 1 mM NADH, 2 μM FMN, 2 μM NtfO1–NtfO2. After incubation at 30 °C for 3 h, 100 μL acetonitrile was added to quench the reaction. Then, the reaction mixture was centrifuged at 18,500 × *g* for 10 min, and the supernatant was subjected to LC-MS analysis. The LC-MS analysis was performed with an 18 min gradient elution system from 10 to 90% (1–13 min), 100% (13–15 min) and 10% (15–18 min) methanol in water supplied with 0.1% formic acid at a flow rate of 0.5 mL/min.

### In vitro assay of StmK

The StmK catalyzed reaction was carried out in a 100 μL reaction system containing 50 mM PIPES buffer (pH 7.1), 200 μM substrate, 1 mM NADH, 1 mM NADPH, 20 μM FMN, 100 mM NaCl, 10 mM KCl, 2 μM NgnO1, 2 μM NgnO2 and 80 μM StmK. After incubation at 30 °C for 4 h, 100 μL acetonitrile was added to quench the reaction, and then centrifuged at 18,500 × *g* for 10 min. The supernatant was subjected to LC-MS analysis. The LC-MS analysis was performed with an 18 min gradient elution system from 10 to 90% (1–13 min), 100% (13–15 min), and 10% (15–18 min) methanol in water supplied with 0.1% formic acid at a flow rate of 0.5 mL/min.

### In vitro assay of StmK-catalyzed reverse reaction

The reverse-reaction of StmK was carried out in a 100 μL system containing 50 mM PIPES buffer (pH 7.1), 100 μM compound **11** as substrate, 2 mM NADP^+^, 100 mM NaCl, 10 mM KCl and 40 μM StmK. After incubation at 30 °C for about 2 h, 100 μL acetonitrile was added to quench the reaction, and then centrifuged at 18,500 × *g* for 10 min. The supernatant was subjected to LC-MS analysis. The LC-MS analysis was performed with an 18 min gradient elution system from 10 to 90% (1–13 min), 100% (13–15 min) and 10% (15–18 min) methanol in water supplied with 0.1% formic acid at a flow rate of 0.5 mL/min.

### Analytical size-exclusion chromatography

The molecular weights (MW) and quaternary state of NtfO1 and NtfO2 complex in solution were determined by size-exclusion chromatography using a Superose 6 Increase 10/300 GL column (GE Healthcare LifeSciences) connected to an ÄKTA Express system (GE Healthcare LifeSciences). The column was pre-equilibrated with two column volumes of 50 mM MES buffer, pH 6.7, and calibrated with ribonuclease A (13.7 kDa), ovalbumin (44 kDa), conalbumin (75 kDa), IgG (150 kDa), and ferritin (440 kDa). The chromatography was carried out at 4 °C at a flow rate of 0.3 mL min^−1^. The column void volume was determined by using Blue Dextran as standard. The calibration curve of *K*_av_ versus log(MW) was prepared using Eq. 1

1 $${K}_{{\mathrm{av}}} = \left( {{V}_{\mathrm{e}}-{V}_{\mathrm{o}}} \right)/\left( {{V}_{\mathrm{t}}-{V}_{\mathrm{o}}} \right)$$

where *V*_e_, *V*_o_, and *V*_t_ is the elution volume, column void volume, and total bed volume, respectively.

### Computational details

The DFT calculations were performed with the Gaussian 09 program package^34^. The geometry optimizations of minima and transition states involved (with two or one explicit water molecule or without water, Fig. 4 and Supplementary Fig. 9) were carried out at the B3LYP-D3 level of theory^35^ with the 6–31 G(d) basis set. The vibrational frequencies were computed at the same level to check whether each optimized structure is an energy minimum or a transition state and to evaluate its zero-point vibration energy (ZPVE) and thermal corrections at 298 K. Solvation energies were computed at the M06-2X level of theory^36,37^ with the 6-311 + G(d,p) basis set using the gas-phase optimized structures and the CPCM model^38–40^ in water.

### Protein crystallization, structural elucidation, and docking study

Crystals were grown at 22 °C with the sitting-drop vapor-diffusion method. 1 μL drop consisted of 1:1 ratio of proteins (10 mg/mL, 50 mM NaCl, 20 mM Tris, pH 8.0) and crystallization buffer (1.6 M magnesium sulfate, 0.1 M MES monohydrate pH 6.5). Crystals of NtfO1 and NtfO2 complex were briefly soaked in the crystallization buffer containing additional 15% glycerol before flash-freezing for protection.

A single-wavelength anomalous diffraction data of the NtfO1 and NtfO2 complex was collected at BL17U1 beamline at the Shanghai Synchrotron Radiation Facility (SSRF) at wavelengths of 0.97918 Å^41^. The diffraction datasets were processed and scaled using imosflm^42^. The phase problem of the complex was solved by the molecular replacement method using the structure of the YTNJ protein (PDB ID: 1TVL) as the search model with PHASER^43^ and further autobuilded and refined by PHENIX^44^, COOT was used for manually model rebuilding and adjustments^45^. Finally, additional TLS refinement was performed in PHENIX. The final refinement statistics are listed in Supplementary Table 9. Structural diagrams were prepared using the program PyMOL (http://www.pymol.org/). The FMN was docked into the putative binding pocket by using Autodock Vina^46^.

### Site-directed mutagenesis of NtfO2

Mutated fragments were amplified with primers listed in Supplementary Table 2 by using plasmid pHG02047 as template. The purified PCR products were incubated with *Dpn*I, T4 polynucleotide kinase, and T4 DNA ligase, according to the standard procedure of Q5^®^ Site-Directed Mutagenesis Kit purchased from NEB (USA). Each mutation was confirmed by sequencing. The recombined plasmids were expressed in *E. coli* BL21(DE3) and purified as described above for native protein.

### Reporting summary

Further information on research design is available in the Nature Research Reporting Summary linked to this article.

## Supplementary information

Supplementary Information

Supplementary Data 1

Description of Additional Supplementary Files

Reporting Summary

## Data Availability

Data supporting the findings of this work are available within the paper and its Supplementary Information files. A reporting summary for this Article is available as a Supplementary Information file. All additional data supporting the current study in the article or its Supplementary Information files are available from the corresponding author upon request. Atomic coordinates of NtfO1–NtfO2 have been deposited in the Protein Data Bank (PDB) with accession codes 7E36 Data of the sequences supporting this work have been deposited in Genbank with accession codes MG891745 for streptoseomycin gene cluster and MH544245 for nargenicin gene cluster. The source data underlying Supplementary Figs. 5f, 7, and 14 are provided as a Source Data file. Source data are provided with this paper.

## Source data

Source Data
