# Supplementary material for: A [6+4]-cycloaddition adduct is the biosynthetic intermediate in streptoseomycin biosynthesis
Source: Nat Commun. 2021 Apr 7;12:2092. doi: 10.1038/s41467-021-22395-7 (PMC8027225; doi:10.1038/s41467-021-22395-7)
Supplement: Supplementary file 1 — Supplementary Information [file 41467_2021_22395_MOESM1_ESM.pdf]

A [6+4]-cycloaddition adduct is the biosynthetic intermediate in streptoseomycin biosynthesis

Wang *et al.*

Supplementary Table 1. Bacterial plasmids and strains.

Plasmid/strain	Relevant characteristics	Reference
**Plasmid**		
pKC1139	*E. coli-Streptomyces* shuttle plasmid used for gene disruption, temperature sensitive	1
pSET152-*kasO*p*	pSET152 derived plasmid containing the promoter *kasO*p*	2
pUWL201	*E. coli/Streptomyces* shuttle vector, containing the promoter *ermE**, ampicillin and thiostrepton resistance	3
pET-22b(+)	Protein expression vector used in *E. coli*, encoding C-terminal His-tag, ampicillin resistance	Novagen
pET-28a(+)	Protein expression vector used in *E. coli*, encoding N-terminal His-tag, kanamycin resistance	Novagen
pHG02034	pKC1139 derived plasmid for disruption of *stmX-stmO3*	This study
pHG02035	pKC1139 derived plasmid for disruption of *stmO1*	This study
pHG02036	pKC1139 derived plasmid for disruption of *stmO2*	This study
pHG02037	pKC1139 derived plasmid for disruption of *stmK*	This study
pHG02038	pSET152-*kasO*p* derived plasmid for complementation of *stmO1* in HG2008 and HG2011	This study
pHG02039	pSET152-*kasO*p* derived plasmid for complementation of *stmO2* in HG2009 and HG2011	This study
pHG02040	pSET152-*kasO*p* derived plasmid for complementation of *stmK* in HG2010 and HG2011	This study
pHG02041	pSET152-*kasO*p* derived plasmid for complementation of *stmO1O2* in HG2011	This study
pHG02042	pSET152-*kasO*p* derived plasmid for complementation of *ngnO1O2* in HG2011	This study
pHG02043	pUWL201 derived plasmid for complementation of *stmK* in HG2015	This study
pHG02044	pET-28a(+) derived plasmid for expressing N-terminal His-tag StmO1	This study
pHG02045	pET-28a(+) derived plasmid for expressing N-terminal His-tag NgnO1	This study
pHG02046	pET-22b(+) derived plasmid for expressing C-terminal His-tag NgnO2	This study
pHG02047	pET-22b(+) derived plasmid for expressing C-terminal His-tag NtfO1-NtfO2	This study
pHG02048	pET-22b(+) derived plasmid for expressing C-terminal His-tag StmK	This study
***E. coli* strains**		
DH5*α*	General cloning host	4
BL21(DE3)	Heterologous host for protein expression	NEB
ET12567/pUZ8002	Methylation-deficient host used for *E. coli-Streptomyces* intergeneric conjugation	1
***S. seoulensis* strains**		
A01	Wild type strain for streptoseomycin (STM) production	5
HG2006	Δ*stmY-stmF1*, in-frame deletion mutant strain in WT, STM non-producing	6
HG2008	Δ*stmO1*, in-frame deletion mutant strain in WT, STM non-producing	This study
HG2009	Δ*stmO2*, in-frame deletion mutant strain in WT, STM non-producing	This study
HG2010	Δ*stmK*, in-frame deletion mutant strain in WT, STM non-producing	This study
HG2011	*stmABCD*, in frame deletion of genes *stmX-stmO3* in Δ*stmY-stmF1*, STM non-producing	This study
HG2012	*stmABCDO1*, complementation of gene *stmO1* to strain *stmABCD*, STM non producing	This study
HG2013	*stmABCDO2*, complementation of gene *stmO2* to strain *stmABCD*, STM non producing	This study
HG2014	*stmABCDK*, complementation of gene *stmK* to strain *stmABCD*, STM non producing	This study
HG2015	*stmABCDO1O2*, complementation of genes *stmO1O2* to strain *stmABCD*, STM non-producing	This study
HG2016	*stmABCDngnO1O2*, complementation of genes *ngnO1O2* to strain *stmABCD*, STM non-producing	This study
HG2017	*stmABCDO1O2K*, complementation of gene *stmK* to strain *stmABCDO1O2*, STM non-producing	This study
HG2018	complementation of Δ*stmO1* mutant by *stmO1*, STM producing	This study
HG2019	complementation of Δ*stmO2* mutant by *stmO2*, STM producing	This study
HG2020	complementation of Δ*stmK* mutant by *stmK*, STM producing	This study

Supplementary Table 2. Primers used in this study.

Name	Sequence [a]	Enzyme sites
**For amplification of homologous arms from genomic DNA for gene disruption (5’-3’)**		
Δ*stmX-stmO3*-UP-F	AACGACGGCCAGTGCCAAGCTTACCCTCCTCGTACTGTTCTCTT	*Hind*III
Δ*stmX-stmO3*-UP-R	CTGGCGCAAGATCGCGCTGGCACCCGTGTTCGTGCT	
Δ*stmX-stmO3*-Down-F	AGCGCGATCTTGCGCCAG	
Δ*stmX-stmO3*-Down-R	AGCTATGACATGATTACGAATTCGTCGCACTTCGCCAACTATCA	*EcoR*I
Δ*stmO1*-UP-F	AACGACGGCCAGTGCCAAGCTTCATCGTGACCGCCAACTA	*Hind*III
Δ*stmO1*-UP-R	GAGGGAGGGATCGACGCCACGGTACGGCAGAAGTTC	
Δ*stmO1*-Down-F	GGCGTCGATCCCTCCCTC	
Δ*stmO1*-Down-R	AGCTATGACATGATTACGAATTCACGAGTTCCTGCGCTACTTCA	*EcoR*I
Δ*stmO2*-UP-F	AACGACGGCCAGTGCCAAGCTTTCCGCCAGGTCATCGATTT	*Hind*III
Δ*stmO2*-UP-R	CGGCTGGGAGCGATCATCGTCACGGGTAGGTGTGCA	
Δ*stmO2*-Down-F	GATGATCGCTCCCAGCCG	
Δ*stmO2*-Down-R	AGCTATGACATGATTACGAATTCTTCCTTGCGAGATTCGGATACC	*EcoR*I
Δ*stmK*-UP-F	AACGACGGCCAGTGCCAAGCTTCTGGGTCAGGAAGCTGATGAA	*Hind*III
Δ*stmK*-UP-R	GTGAAGTACGTTCCTCTCCGGAGCCGGACGATCGAC	
Δ*stmK*-Down-F	GAGAGGAACGTACTTCAC	
Δ*stmK*-Down-R	AGCTATGACATGATTACGAATTCTTCCGATGTCGTCCAAG	*EcoR*I
**For screening of the double-crossover mutants (5’-3’)**		
ScreenΔ*stmX-stmO3*-F	TCGAGCGCGGATTCGATG	
ScreenΔ*stmX-stmO3*-R	TGCTGGGTGATTCCGAGATT	
ScreenΔ*stmO1*-F	CCGTTCGCGAGTTTCGT	
ScreenΔ*stmO1*-R	CGGGTTCATCGTCGACTTC	
ScreenΔ*stmO2*-F	GTACTCGGTGGAGATCGTG	
ScreenΔ*stmO2*-R	GGAAGCTGCTCATCGACT	
ScreenΔ*stmK*-F	CACGGCTTCACCGATGT	
ScreenΔ*stmK*-R	ATCCGAACCGCATGACC	
**For genes complementation (5’-3’)**		
152-StmO1-F	CTATGACATGATTACGAATTCACTGCGCGACAATCCTCTGTA	*EcoR*I
152-StmO1-R	TGCTGCATGCATACGTACTAGTCGGGATCGCTGGACGACTT	*Spe*I
152-StmO2-F	CTATGACATGATTACGAATTCTGACCGTCATTGCACACCTACCC	*EcoR*I
152-StmO2-R	TGCTGCATGCATACGTACTAGTCATGCCGGTGCGCTTCACC	*Spe*I
152-StmK-F	CTATGACATGATTACGAATTCACGTTCGGCGTCCACTCGATCA	*EcoR*I
152-StmK-R	TGCTGCATGCATACGTACTAGTCGGTACGTGGCGGAGCACCAT	*Spe*I
152-StmO1O2-F	TGCTGCATGCATACGTACTAGTACGAGTTCCTGCGCTACTTC	*Spe*I
152-StmO1O2-R	CTATGACATGATTACGAATTCCGCGACAATCCTCTGTACG	*EcoR*I
152-NgnO1O2-F	TGCTGCATGCATACGTACTAGTAATCTGGATGCACAGGTCTTC	*Spe*I
152-NgnO1O2-R	CTATGACATGATTACGAATTCGAGATTCGGATATGCGCTCA	*EcoR*I
201-StmK-F	AAAGAGGAGAAATTACATATGACCATCCGAACCGCATGAC	*Nde*I
201-StmK-R	CAGGAATTCGATATCAAGCTTGGTCGACGCCATGATCACGG	*Hind*III
**For protein expression (5’-3’)**		
28a-StmO1-F	GGTGCCGCGCGGCAGCCATATGACGGTCACGCTGCTCACT	*Nde*I
28a-StmO1-R	GCTCGAGTGCGGCCGCAAGCTTTCAGCGGCCCGCCGGGCG	*Hind*III
28a-NgnO1-F	GGTGCCGCGCGGCAGCCATATGACCCTCGTACTCTTCGCG	*Nde*I

28a-NgnO1-R	GCTCGAGTGCGGCCGCAAGCTTTCACGCCGCGCTCCATTT	*Hind*III
22b-NgnO2-F	AAGAAGGAGATATACATATGCCCCAATCCACAAGATCACT	*Nde*I
22b-NgnO2-R	CTCGAGTGCGGCCGCAAGCTTTGCCGCCGGGCGTCGGCCGA	*Hind*III
22b-StmK-F	AAGAAGGAGATATACATATGAAGTACGTTCCTCTCGGC	*Nde*I
22b-StmK-R	CTCGAGTGCGGCCGCAAGCTTCGGAGCCGGACGATCGAC	*Hind*III
22b-NtfO1O2-F	AAGAAGGAGATATACATATGCCCCAATCCACAAGATCACT	*Nde*I
22b-NtfO1O2-R	CTCGAGTGCGGCCGCAAGCTTTGCCGCCGGGCGTCGGCCGA	*Hind*III
NtfO1O2-F222A-F	GGAGGTGATCgcaACCAGGCACACGCAGC	
NtfO1O2-F222A-R	CCGTGTCGCGCCGCGAAA	
NtfO1O2-Y155A-F	CGACCTGCGCgccACCAGAGCGG	
NtfO1O2-Y155A-R	TGTTCCATGTGCTCGGGG	
NtfO1O2-Q203A-F	GGTGATCTTCgccGCCGGTGCCTCGCCGGTCGGACG	
NtfO1O2-Q203A-R	GGCCGCCCCTGCGGCGTC	
NtfO1O2-H151A-F	GCACATGGAAgccGACCTGCGCTAC	
NtfO1O2-H151A-R	TCGGGGAACCCGAAGTTG	
NtfO1O2-T133A-F	GAACGTGGTCgccTCGGCCGCGC	
NtfO1O2-T133A-R	CAGCCGGACCGGCCACCG	
NtfO1O2-T101A-F	TGTCGTCGCGgccGCGACGACGA	
NtfO1O2-T101A-R	CCGAGATGCTCGGTGCTCGCC	
NtfO1O2-F56A-F	CGATTGCGTGgcaGTCGCGGACG	
NtfO1O2-F56A-R	AATATCCCGCGCTCCAAT	

Supplementary Table 3. Annotation of the *stm* cluster.

Gene	Size [a]	Proposed function	homolog [b]	Identity%/similarity%
stmD	152	Bispericyclase	*ngnD* (AXG22404.1)	86/91
stmY	256	Alpha/beta hydrolase family	(WP_030241324.1)	89/83
stmG	368	AMP-binding enzyme/ conserved hypothetical protein	*ttnN* (ABV91299.1)	81/67
stmH	402	Coa transferases	*ttnP* (ABV91301.1)	77/68
stmM1	253	Methyltransferase	(EWM14171.1)	59/46
stmI	475	Esterase	*ttnK* (ABV91296.1)	62/49
stmJ	262	Protein tyrosine/serine phosphatase	(WP_030241324.1)	85/80
stmO1	315	LLM class flavin-dependent oxidoreductase	*ngnO1* (AXG22411.1)	56/66
stmO2	415	Pristinamycin IIA synthase subunit A	*ngnO2* (AXG22410.1)	82/90
stmP1	421	Cytochrome P450	*ngnP1* (AXG22409.1)	78/89
stmM2	281	Methyltransferase	*ngnM* (AXG22413.1)	55/68
stmK	349	Dehydratase	*ngnK* (AXG22408.1)	70/82
stmL	290	Phosphotransferase enzyme	(WP_051710832.1)	88/83
stmF	64	Ferredoxin-2	*ngnF*(AXG22414.1)	41/55
stmP2	390	Cytochrome P450	*ngnP2* (AXG22415.1)	41/58
stmM3	253	Methyltransferase	NCAST_32_10170 (GAD86530.1)	65/56
stmN	257	Citryl coa lyase	*ttnO* (ABV91300.1)	76/58
stmQ	275	Putative cyclase	*ttnS* (ABV91304.1)	71/57
stmP3	392	Cytochrome P450	Tcs_55098_003 (ADU56356.1)	83/73
stmR	276	Citryl coa lyase	*ttnO* (ABV91300.1)	57/41
stmS	471	Dehydratase	*ttnR* (ABV91303.1)	75/66
stmT	316	Snoal-like polyketide cyclase	(ELP69668.1)	68/53
stmU	248	Unknow	no hit	
stmO4	331	Taurine dioxygenase	ADK34_41075 (KOG07028.1)	75/60
stmV	257	Thioesterase	*ngnV* (AXG22403.1)	54/67
stmP4	405	Cytochrome P450	*ttnI* (ABV91294.1)	47/36
stmM4	260	Methyltransferase	(WP_007029563.1)	57/45
stmW	495	Multidrug resistance protein	(ABW10189.1)	57/41
stmF	67	Ferredoxin	(KIE53750.1)	65/57
stmA	5966	Type I polyketide synthase	*ngnA* (AXG22407.1)	68/77
stmB	4778	Type I polyketide synthase	*ngnB* (AXG22406.1)	64/73
stmC	3665	Type I polyketide synthase	*ngnC* (AXG22405.1)	64/74
stmX	211	Uncharacterized protein	(WP_030246067.1)	94/89
stmO3	270	Iron-α-ketoglutaratedependent dioxygenase	*ngnO3*(AXG22416.1)	51/66

[a]Numbers are in amino acids.

[b]Given in numbers are NCBI accession numbers.

Supplementary Table 4. Annotation of the *stmO1* and *stmO2* homologues and their neighboring genes in *Nocardia tenerifensis* NBRC 101015.

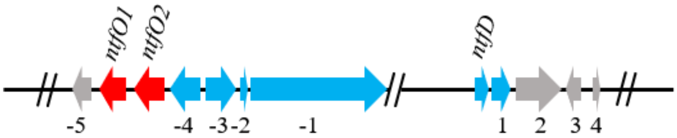

Protein	Size(aa)	Proposed function	Homolog[a]	Identity/similarity, (%/%)
Orf_-5	283	hypothetical protein	TU76_02335 (KMN02617.1)	53/72
NtfO1	324	LLM class flavin-dependent oxidoreductase	StmO1 (AWH12914.1)	53/64
NtfO2	421	pristinamycin IIA synthase subunit A	StmO2 (AWH12915.1)	82/90
Orf_-4	423	cytochrome P450	StmP1 (AWH12916.1)	75/87
Orf_-3	394	cytochrome P450	StmP3 (AWH12925.1)	76/86
Orf_-2	63	ferredoxin	StmF1 (AWH12935.1)	76/87
Orf_-1	2771	type I polyketide synthase	StmC (AWH12938.1)	47/58
NtfD	152	nuclear transport factor 2 family protein	StmD (AWH12907.1)	82/90
Orf_1	256	thioesterase	StmV (AWH12931.1)	50/63
Orf_2	1214	DNA polymerase III subunit alpha	WP_081869253.1	86/93
Orf_3	112	hypothetical protein	CLV40_101308 (PPK71119.1)	58/67
Orf_4	59	hypothetical protein	WP_043660705.1	58/72

[a]Given in numbers are NCBI accession numbers. Gene marked in red is the homolog of *stmO1* and *stmO2*; genes marked in blue is those conserved in *stm* gene cluster.

Supplementary Table 5. NMR spectroscopic data of compound 8 (acetone-d_6).

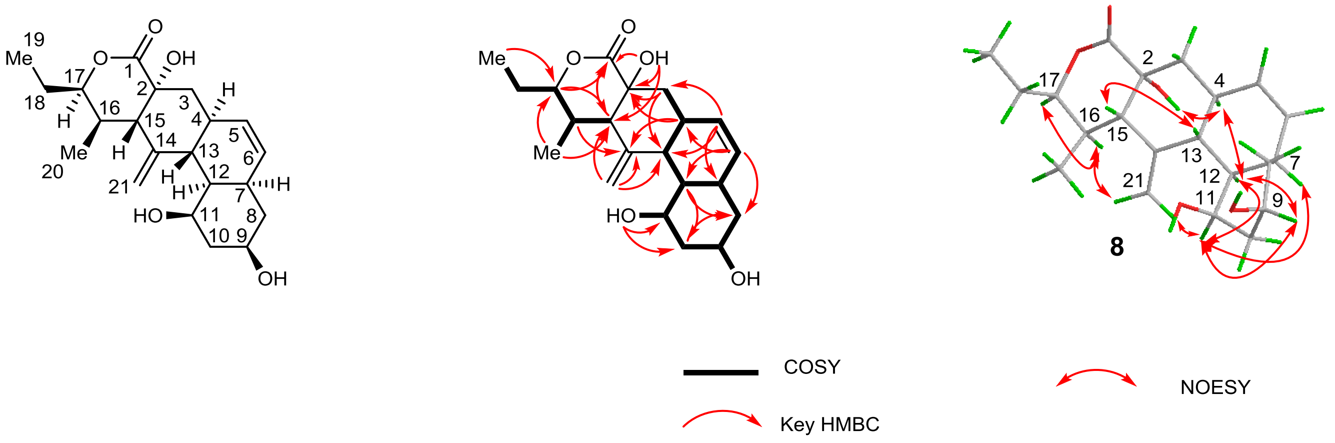

No.	δ_C	δ_H (J in Hz)	COSY	HMBC	NOESY
1	172.2, C				
2	73.3, C				
3	42.1, CH2	1.68, m	H4	1, 2, 5, 13, 15	
		2.24, m			
4	32.5, CH	2.22, m	H3, H5, H13	2, 6, 12, 14	2-OH, H12
5	126.7, CH	5.42, dt (9.6, 2.0)	H4, H6	3, 4, 7, 13	
6	131.9, CH	5.54, ddd (9.6, 3.8, 2.4)	H5, H7	4, 7, 8, 12	
7	39.5, CH	2.26, m	H6, H8, H12	5,	H11
8	37.5, CH2	1.56, ddd (13.0, 11.3, 8.7)	H7, H9	6, 10	
		1.94, m			
9	67.1, CH	3.95, m	H8, H10, 9-OH		H12
10	38.1, CH2	1.81, m	H9, H11	8	
		2.39, m			
11	68.9, CH	4.17, m	H10, H12, 11-OH		H7, H12
12	38.3, CH	1.83, d (6.3)	H7, H11, H13	8, 10	H4, H9, H11
13	44.9, CH	2.22, d (6.3)	H4, H12	3, 5, 15, 21	H15
14	146.5, C				
15	55.4, CH	2.21,d (6.5)	2-OH, H16	1, 13, 20, 21	H13
16	29.2, CH	2.42, m	H15, H17, H20	14, 15, 20	H17, H21
17	79.7, CH	5.03, m	H16, H18	1, 15, 18, 19, 20	H16
18	24.3, CH2	1.64, m	H17, H19	16, 17, 19	
19	10.1, CH3	1.02, t (7.4)	H18	17, 18	
20	14.3, CH3	0.96, d (6.9)	H16	2, 15, 16, 17	
21	106.7, CH2	5.12, s		2, 13, 14, 15	H16
		5.38, s			
	2-OH	4.38, s	H15	1, 2, 3, 15	
	9-OH	3.69, d (3.9)	H9		
	11-OH	3.27, d (6.9)	H11	10, 11	

Supplementary Table 6. NMR spectroscopic data of compound 9 (acetone-d_6).

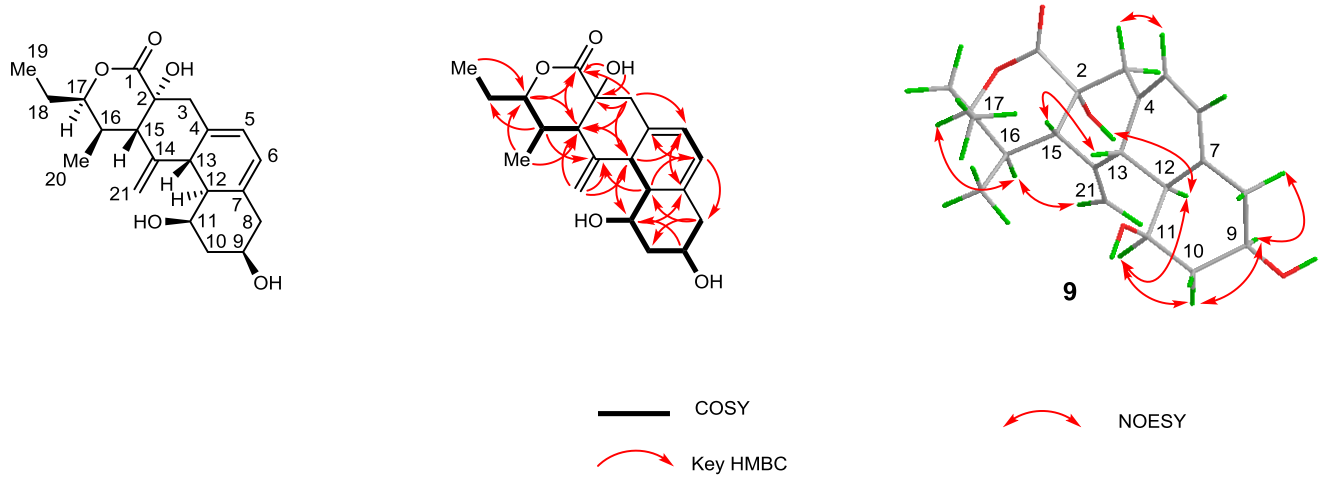

No.	δ_C	δ_H (J in Hz)	COSY	HMBC	NOESY
1	171.7, C				
2	74.1, C				
3	43.7, CH2	2.60, s	H5, H13	1, 2, 4, 5, 13, 15	H5
4	133.6, C				
5	118.4, CH	5.48, d (5.3)	H3, H6, H13	3, 6, 7, 13	
6	120.1, CH	5.67, d (5.3)	H5, H8, H12	4, 5, 8, 12	
7	131.7, C				
8	42.5, CH2	2.44, m	H6, H9	6, 10, 12	H9
		2.49, dd (13.9, 8.2)			
9	69.9, CH	4.24, br s	H8, H10	11	H8, H10
10	38.1, CH2	1.95, dt (12.0, 2.3)	H9, H11	8	H9, H11
		2.11, m			
11	75.3, CH	3.65, br s	H10, H12, 11-OH	7, 9, 10, 13	H10, H12
12	43.9, CH	2.95, d (6.3)	H6, H11, H13	6, 8, 11, 14	H11, 2-OH
13	47.3, CH	3.06, d (6.3)	H5, H12, H21	3, 5, 11, 15, 21	H15
14	146.7, C				
15	54.5, CH	2.26,d (6.5)	H14, H16	1, 3, 13, 17, 20	H13
16	29.8, CH	2.46, m	H15, H17, H20	14, 15, 20	H17, H21
17	79.3, CH	5.01, m	H16, H18	1, 15, 19, 20	H16
18	24.4, CH2	1.66, m	H17, H19	16, 17, 19	
19	10.1, CH3	1.01, t (7.4)	H18	17, 18	
20	14.3, CH3	0.96, d (7.0)	H16	15, 16, 17	
21	106.1, CH2	5.08, s	H13, H15	13, 14, 15	
		5.15, s			H16
	2-OH	4.17, br s		1, 2, 15	H12
	11-OH	4.09, d (4.1)	H11		

Supplementary Table 7. NMR spectroscopic data of compound 10 (acetone-d_6).

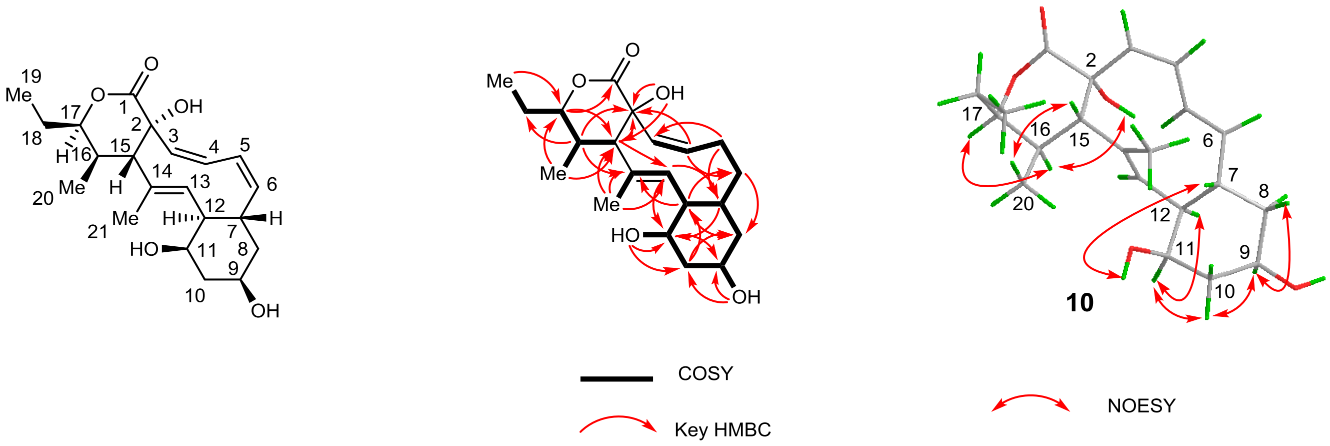

No.	δ_C	δ_H (J in Hz)	COSY	HMBC	NOESY
1	171.7, C				
2	79.4, C				
3	130.5, CH	6.29, dd (10.0, 5.6)	H4	5	
4	130.7, CH	5.66, dd (10.0, 4.5)	H3, H5	2, 6	
5	128.3, CH	6.27, dd (10.2, 5.0)	H4, H6	3, 7	
6	133.4, CH	5.11, dt (11.2, 3.2)	H5, H7	5, 7, 8, 12	
7	32.0, CH	2.61, m	H6, H8, H12	5, 9, 11, 13	11-OH
8	38.9, CH2	1.59, m	H7, H9	6, 10, 12	H9
		2.06, m			
9	66.9, CH	4.18, br s	H8, H10, 9-OH	11	H8, H10
10	36.8, CH2	1.70, m	H9, H11	8, 12	H9
		2.10, m			
11	69.1, CH	3.92, d (4.9)	H10, H12, 11-OH	7, 9	H10, H12
12	47.8, CH	2.17, m	H7, H11, H13	6, 8, 10, 14	H11
13	132.3, CH	5.30, d (10.4)	H12, H20	7, 11,15, 21,	
14	128.9, C				
15	57.6, CH	1.82, d (5.9)	2-OH, H16	2, 3, 13, 14, 17	H20
16	31.2, CH	2.45, qd (11.6, 6.9)	H15, H17, H20	2, 14, 18, 20	H17, 2-OH
17	81.0, CH	4.99, m	H16, H18	1, 15, 19, 20	H16
18	24.3, CH2	1.63, m	H17, H19	16, 17, 19	
19	9.9, CH3	1.01, t (7.4)	H18	17, 18	
20	14.3, CH3	0.91, d (7.0)	H16	15, 16	H15
21	15.9, CH3	1.66, br s	H13	13, 14, 15	
	2-OH	4.92, br s	H15	2, 15	H16
	9-OH	4.51, d (5.0)	H9	9, 10	
	11-OH	4.43, d (7.7)	H11	10, 11	H7

Supplementary Table 8. NMR spectroscopic data of compound 11 (acetone-d_6).

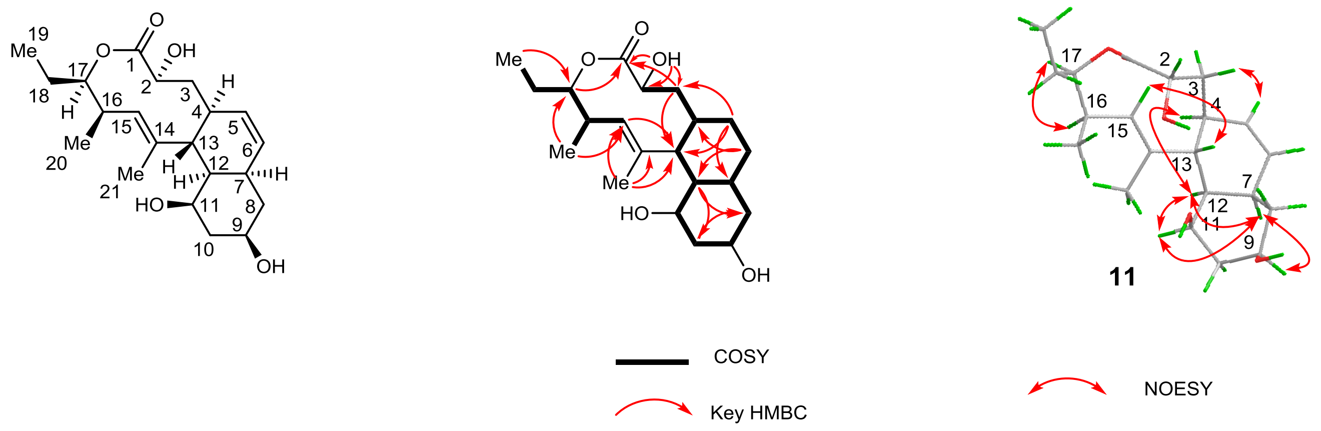

No.	δ_C	δ_H (J in Hz)	COSY	HMBC	NOESY
1	173.3, C				
2	73.8, CH	4.02, dd (11.1, 3.6)	H3, 2-OH	1, 3	
3	43.8, CH2	1.78, m	H2, H4	1, 2, 4, 5, 13	H5
		2.10, m			
4	34.7, CH	1.86, m	H3, H5, H13	2, 3, 6	H12
5	131.0, CH	5.58, dd (9.8, 2.8)	H4, H6	3, 4, 7	
6	128.7, CH	5.68, ddd (9.8, 5.0, 2.2)	H5, H7	4, 5, 7	
7	33.3, CH	1.97, ddd (13.1, 9.1,5.0)	H6, H8, H12	4, 5, 8, 9, 11, 12	H9, H11, H12
8	38.4, CH2	1.11, m	H7, H9	6, 9, 12	
		1.81, m		5, 6, 7, 9, 12,	
9	68.1, CH	3.58, ddd (15.7, 11.0, 4.6)	H8, H10	7, 10, 11	H7
10	39.8, CH2	1.52, dd (23.4, 11.8)	H9, H11	8, 9, 12	
		1.88, m		9, 11, 12,	
11	72.1, CH	3.71, dt (12.3, 3.5)	H10, H12	7, 9, 10, 12,	H7, H12
12	38.7, CH	2.17, dt (12.3, 4.3)	H7, H11, H13	7, 11, 6, 13	H4, H7, H11
13	47.7, CH	2.28, dd (12.3, 9.0)	H4, H12	11, 15, 20	H15
14	137.6, C				
15	130.3, CH	5.07, d (7.1)	H16	2, 3, 13, 16, 17, 21	H13
16	34.1, CH	2.87, td (13.8, 7.1)	H15, H17, H20	2, 15, 21	H17
17	77.1, CH	5.21, dt (8.3, 5.8)	H16, H18	15, 18, 19, 21	H16
18	23.7, CH2	1.63, m	H17, H19	16, 17, 19	
19	10.2, CH3	0.92, t (7.3)	H18	17, 18	
20	14.6, CH3	1.06, d (7.1)	H16	13, 14, 15	
21	13.2, CH3	1.65, br s		15, 16, 17	
	2-OH	4.47, d (6.0)	H2	1, 2, 3	

Supplementary Table 9. Data collection and refinement statistics (molecular replacement) of NtfO1-NtfO2.

7E36	NtfO1 and NtfO2
**Data collection**	
Space group	$P\ 1\ 2_1\ 1$
Cell dimensions	
a, *b*, *c* (Å)	51.28, 104.99, 75.95
α, β, γ (°)	90, 95.95, 90
Resolution (Å)	51.00-2.00 (2.05-2.00)
R_{merge} (%)	10.0 (57.3)
$I / \sigma I$	7.3 (1.7)
CC(1/2)	0.99 (0.62)
Completeness (%)	99.7 (97.4)
Redundancy	3.3 (2.9)
**Refinement**	
Resolution (Å)	51.00-2.00 (2.070-1.998)
No. reflections	53909
R_{work} / R_{free} (%)	16.45 (23.22) / 20.51 (23.39)
No. atoms	5989
Protein	5459
Ligand/ion	38
Water	492
B-factors	33.5
Protein	33.0
Ligand/ion	51.8
Water	38.4
R.m.s. deviations	
Bond lengths (Å)	0.004
Bond angles (°)	0.70

*Values in parentheses are for highest-resolution shell.

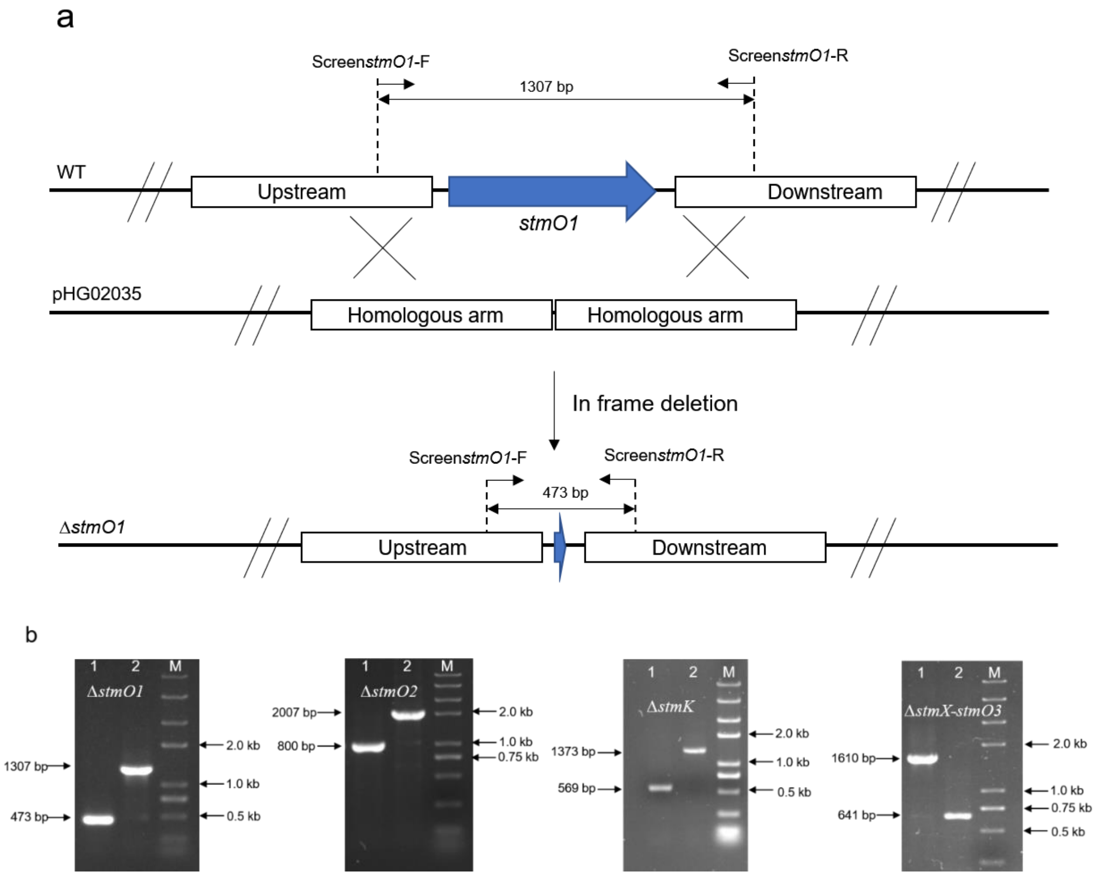

Supplementary Fig. 1. Gene in-frame deletion in *S. seoulensis* A01. (a) The strategies used in gene in-frame deletion. (b) PCR verification of *stm* mutants: Lane1, amplified with Screen-F/R and mutants; Lane 2, amplified with Screen-F/R and WT; Lane M, Trans2K® Plus II DNA marker. And in the Δ*stmX-stmO3* mutant: Lane 1 is amplified with Screen-F/R and WT; Lane 2 is amplified with Screen-F/R and mutant. The experiments were repeated three times with similar results (a representative example is shown).

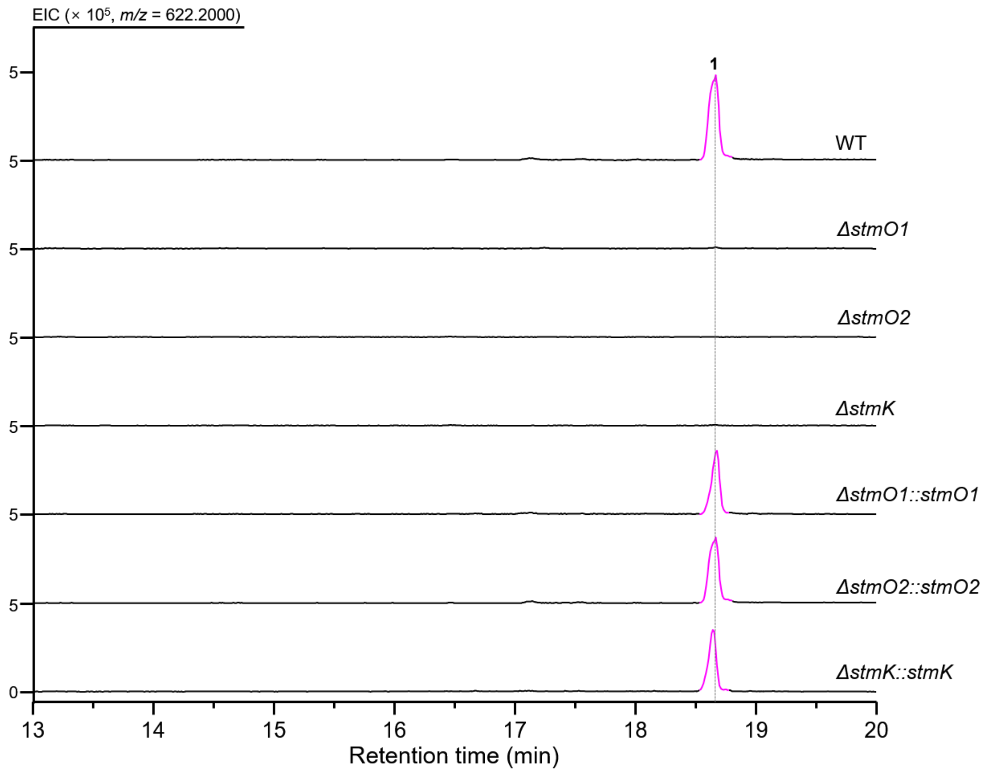

Supplementary Fig. 2. LC-MS analysis of gene complementation for Δ*stmO1*, Δ*stmO2* and Δ*stmK* strains.

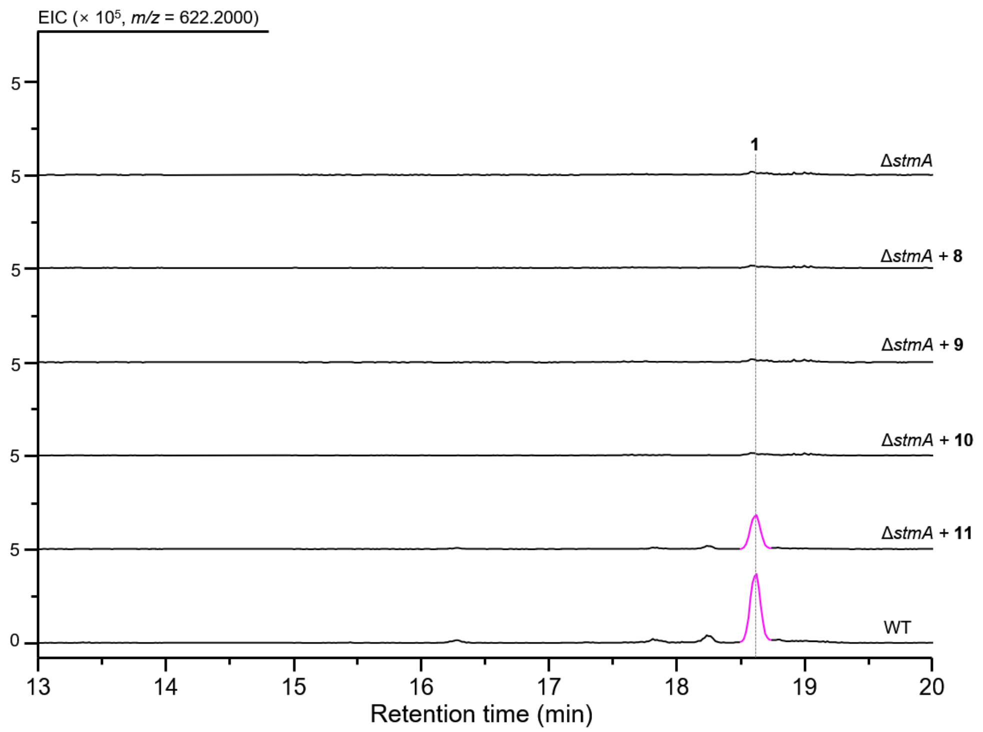

Supplementary Fig. 3. LC-MS analysis of chemical complementation of compounds 8, 9, 10 and 11 in the Δ*stmA* mutant strain.

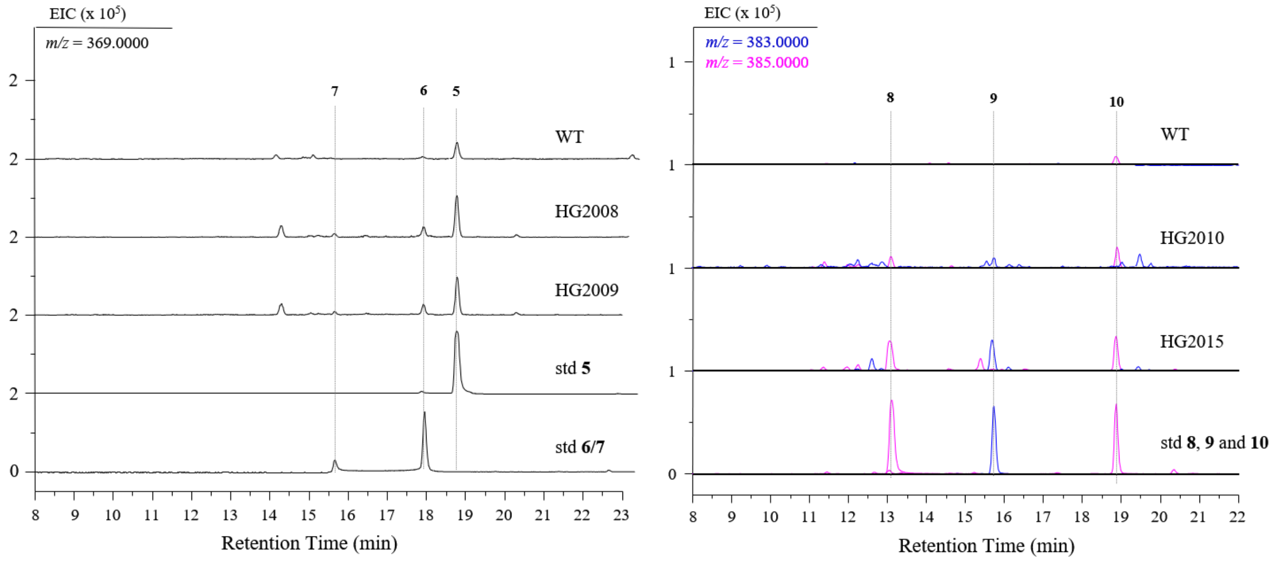

Supplementary Fig. 4. LC-MS analysis of mutant strains Δ*stmO1*, Δ*stmO2* and Δ*stmK*. a) LC-MS analysis of strain WT (wild-type), HG2008 (Δ*stmO1*), HG2009 (Δ*stmO2*), standard of **5** and standards of **6**/**7**. EICs at *m/z* = 369.0 (**5**, **6**, **7**, $[M + Na]^+$). b) LC-MS analysis for strain WT (wild-type), HG2010 (Δ*stmK*), HG2015 (*stmABCDO1O2*) and standard of **8**, **9** and **10**. EICs at *m/z* = 383.0 (**9**, $[M + Na]^+$) and at *m/z* = 385.0 (**8**, **10**, $[M + Na]^+$).

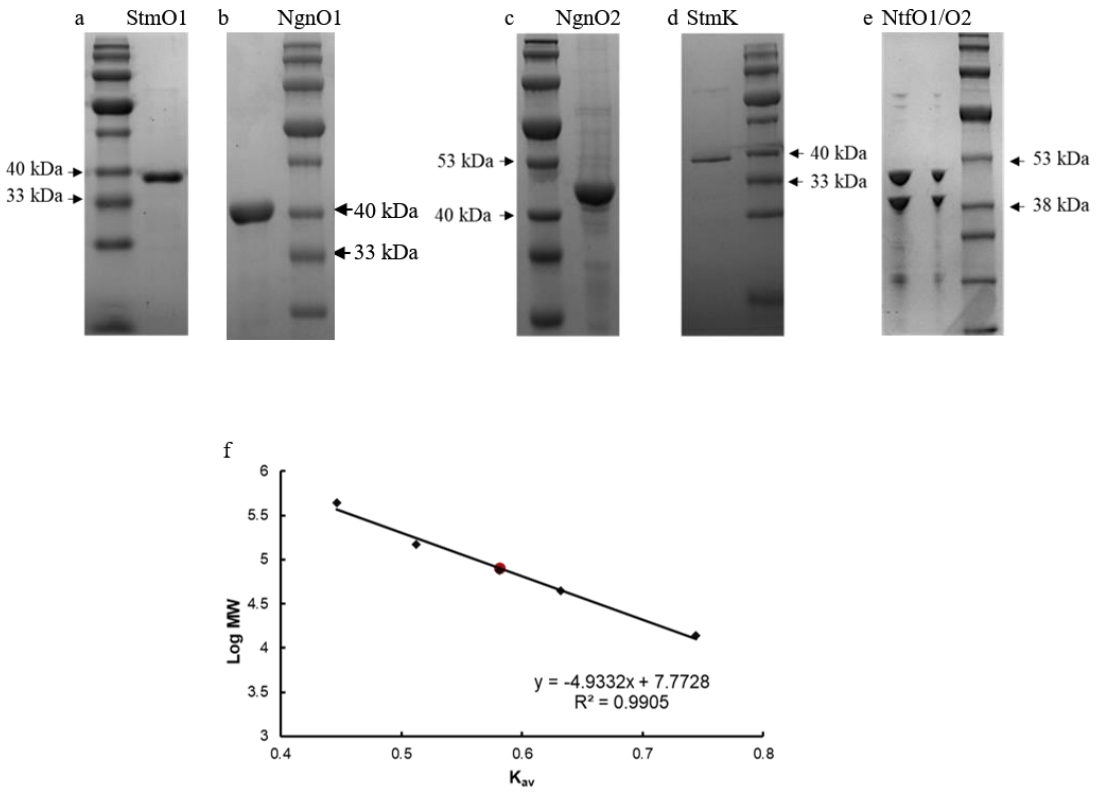

Supplementary Fig. 5. SDS-PAGEs and size-exclusion chromatography of the purified proteins. (a) StmO1 (Molecular weight, 34.99 kDa); (b) NgnO1 (Molecular weight, 34.87 kDa); (c) NgnO2 (Molecular weight, 47.88 kDa); (d) StmK (Molecular weight, 39.82 kDa); (e) NtfO1 and NtfO2 (Molecular weight, 35.57 kDa and 46.36 kDa), which was run in Tris-MOPS buffer, while others were run in Tris-glycine buffer. The SDS-PAGEs were repeated three times with similar results (a representative example is shown). (f) Size-exclusion chromatography of NtfO1 and NtfO2 complex. The complex (red dot) eluted at retention volumes of 17.11 mL, correlating to molecular weights (MWs) of 80.3 kDa, respectively. The calculated MWs for NtfO1 and NtfO2 are ~35.6 and ~46.4 kDa, respectively. Thus, NtfO1 and NtfO2 are formed as a heterodimer in solution. The experiments were repeated twice with similar results (a representative example is shown). Source data underlying Supplementary Figure 5f are provided as a Source Data file.

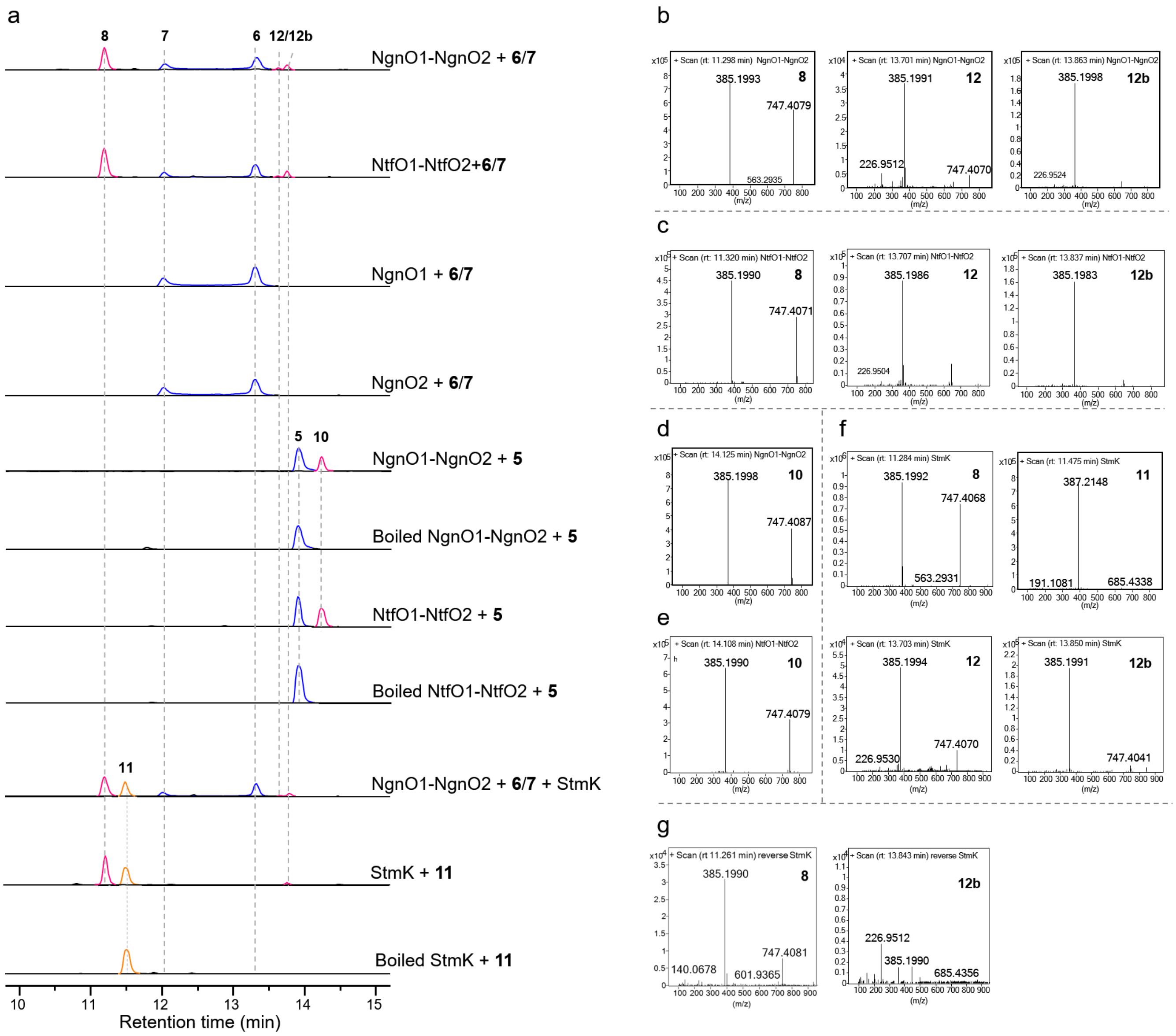

Supplementary Fig. 6. High resolution LC-MS analysis the products of *in vitro* assays. a) LC-MS analysis of NgnO1-NgnO2, StmK and NtfO1-NtfO2 catalyzed reaction. b) the high resolution MS of **8** (*m/z* 385.1993 [M + Na]$^+$), **12** (*m/z* 385.1991 [M + Na]$^+$) and **12b** (*m/z* 385.1998 [M + Na]$^+$) produced by NgnO1-NgnO2 reaction with **6**/**7**. c) The high resolution MS of **8** (*m/z* 385.1990 [M + Na]$^+$), **12** (*m/z* 385.1986 [M + Na]$^+$) and **12b** (*m/z* 385.1983 [M + Na]$^+$) produced by NtfO1-NtfO2 reaction with **6**/**7**. d) The high resolution MS of **10** (*m/z* 385.1990 [M + Na]$^+$) produced by NgnO1-NgnO2 reaction with **5**. e) The high resolution MS of **10** (*m/z* 385.1992 [M + Na]$^+$) produced by NtfO1-NtfO2 reaction with **5**. f) The high resolution MS of **8** (*m/z* 385.1992 [M + Na]$^+$), **11** (*m/z* 387.2148 [M + Na]$^+$), **12** (*m/z* 385.1994 [M + Na]$^+$) and **12b** (*m/z* 385.1992 [M + Na]$^+$) produced by NgnO1-NgnO2 and StmK coupled reaction with **6**/**7**. g) The high resolution MS of **8** (*m/z* 385.1990 [M + Na]$^+$) and **12b** (*m/z* 385.1990 [M + Na]$^+$) produced by StmK-catalyzed reaction using **11**. The calculated HRESIMS datum for $C_{21}H_{30}O_5$ (**8**, **10**, **12** and **12b**) is *m/z* 385.1986 [M + Na]$^+$, the calculated HRESIMS datum for $C_{21}H_{32}O_5$ (**11**) is *m/z* 387.2142 [M + Na]$^+$.

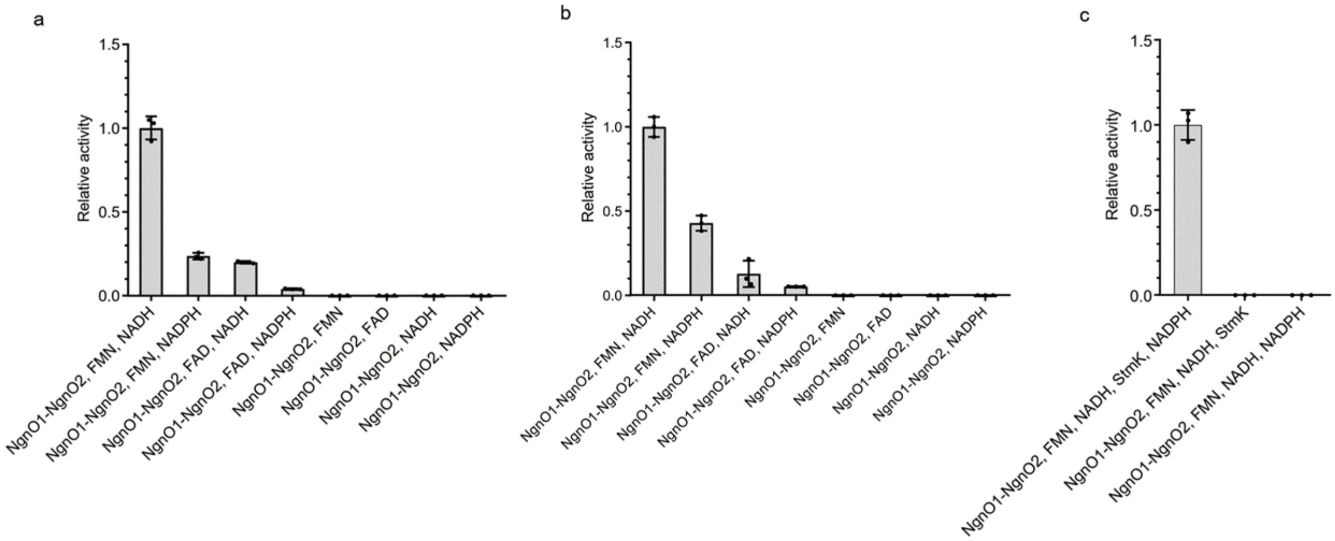

Supplementary Fig. 7. Effects of cofactors on NgnO1-NgnO2 and StmK catalyzed reactions. a) Effects of cofactors on NgnO1-NgnO2 catalyzed reaction using **6**/**7** as substrates. b) Effects of cofactors on NgnO1-NgnO2 catalyzed reaction using **5** as substrate. c) Effects of NADPH on StmK catalyzed reaction when **6**/**7**, NgnO1-NgnO2 and StmK were added in one pot. For each tested condition n=3 independent replicates were analyzed and data are presented as mean values +/- SD. Source data are provided as a Source Data file.

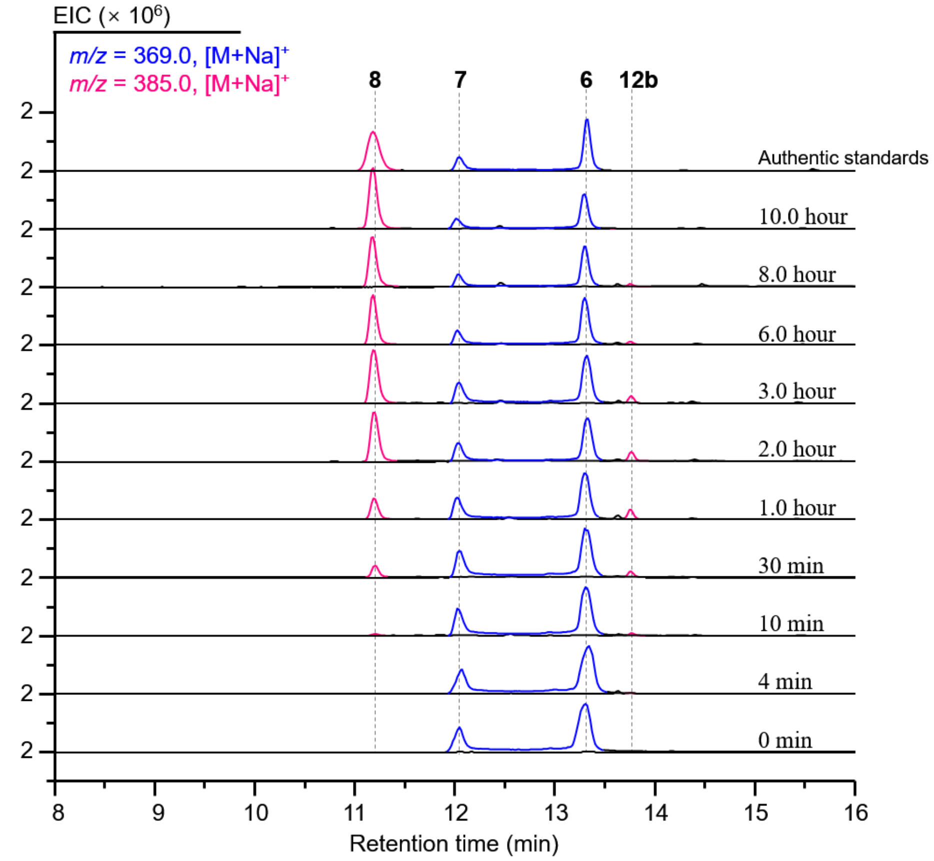

Supplementary Fig. 8. Time-course analysis of NgnO1-NgnO2 catalyzed reaction.

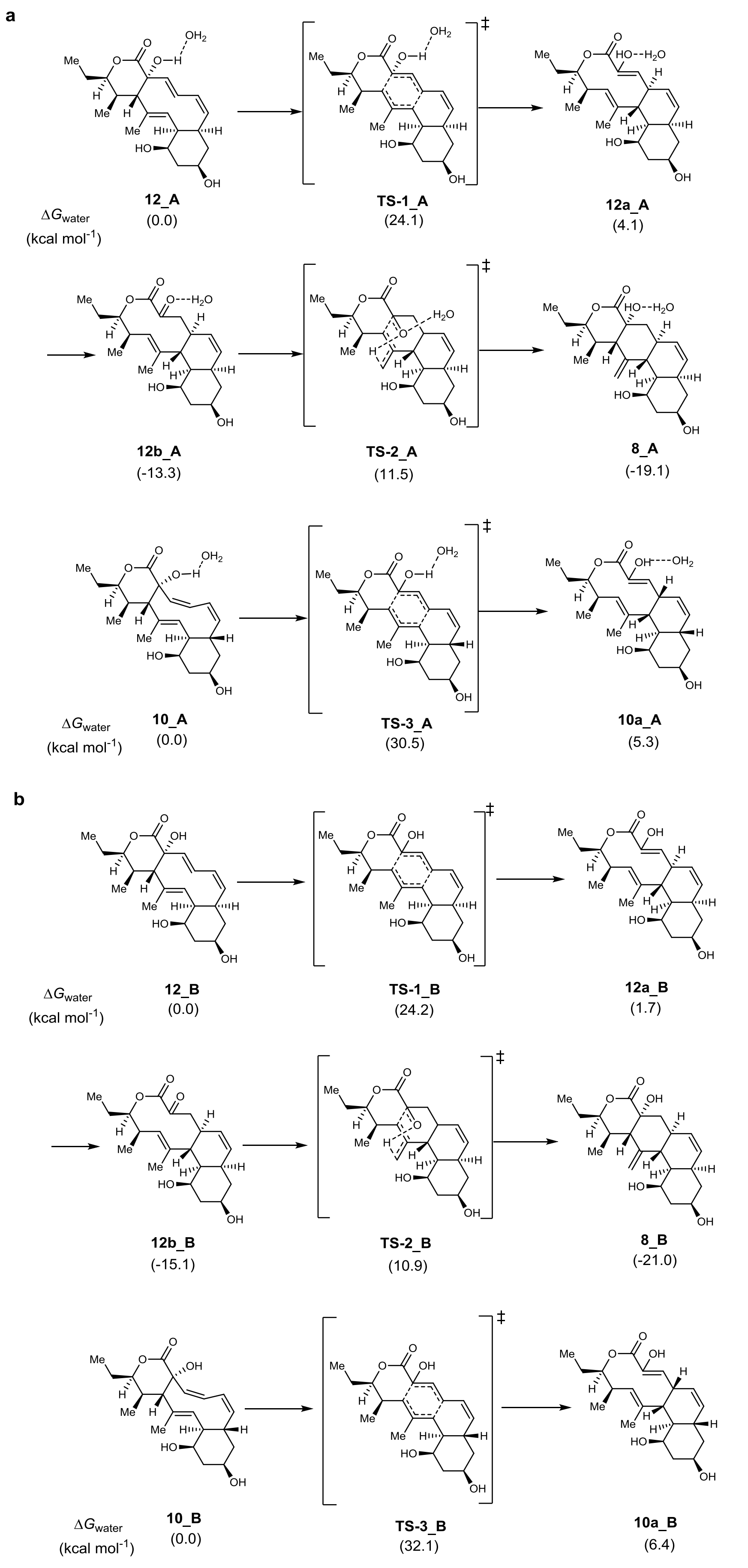

Supplementary Fig. 9. DFT-computed Gibbs free energies with one (a) or no (b) coordinated water molecule.

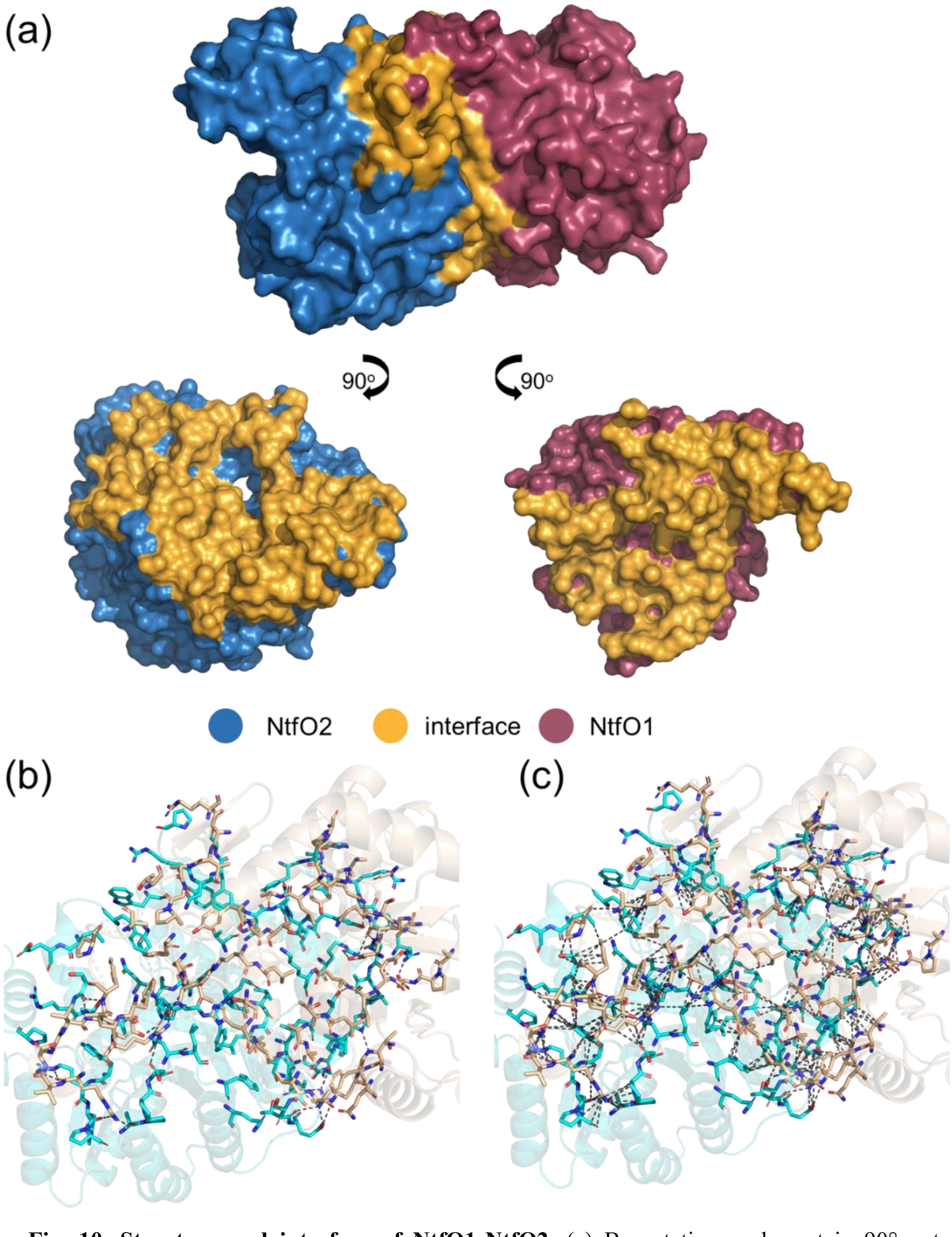

Supplementary Fig. 10. Structure and interface of NtfO1-NtfO2. (a) By rotating each protein 90° outwards, the residues located at the interface residues are colored yellow. (b) the residues of interface and polar interaction indicated by black dash line. (c) the residues of interface and all interactions indicated by black dash line. NtfO1 was presented by wheat and NtfO2 was presented by cyan.

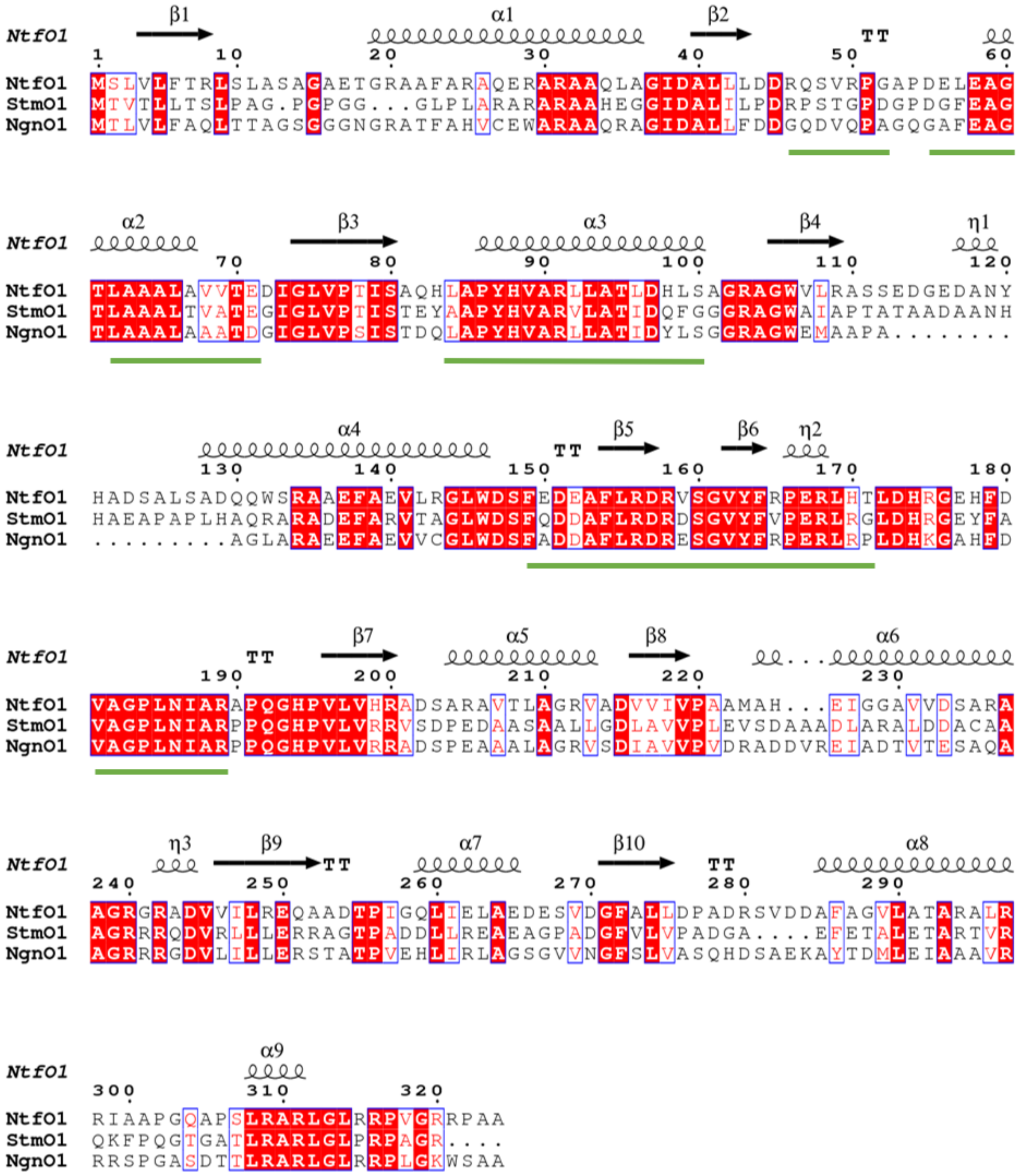

Supplementary Fig. 11. Multiple amino acid sequences alignment of StmO1 and its homologues. The multiple alignment was generated by MUSCLE[7] and rendered with ESPript 3.0[8]. The NCBI accession number of sequences NtfO1, StmO1 and NgnO1 are WP_051187833.1, AWH12914.1 and AXG22411.1, respectively. The green bar under the sequences present the key interface region.

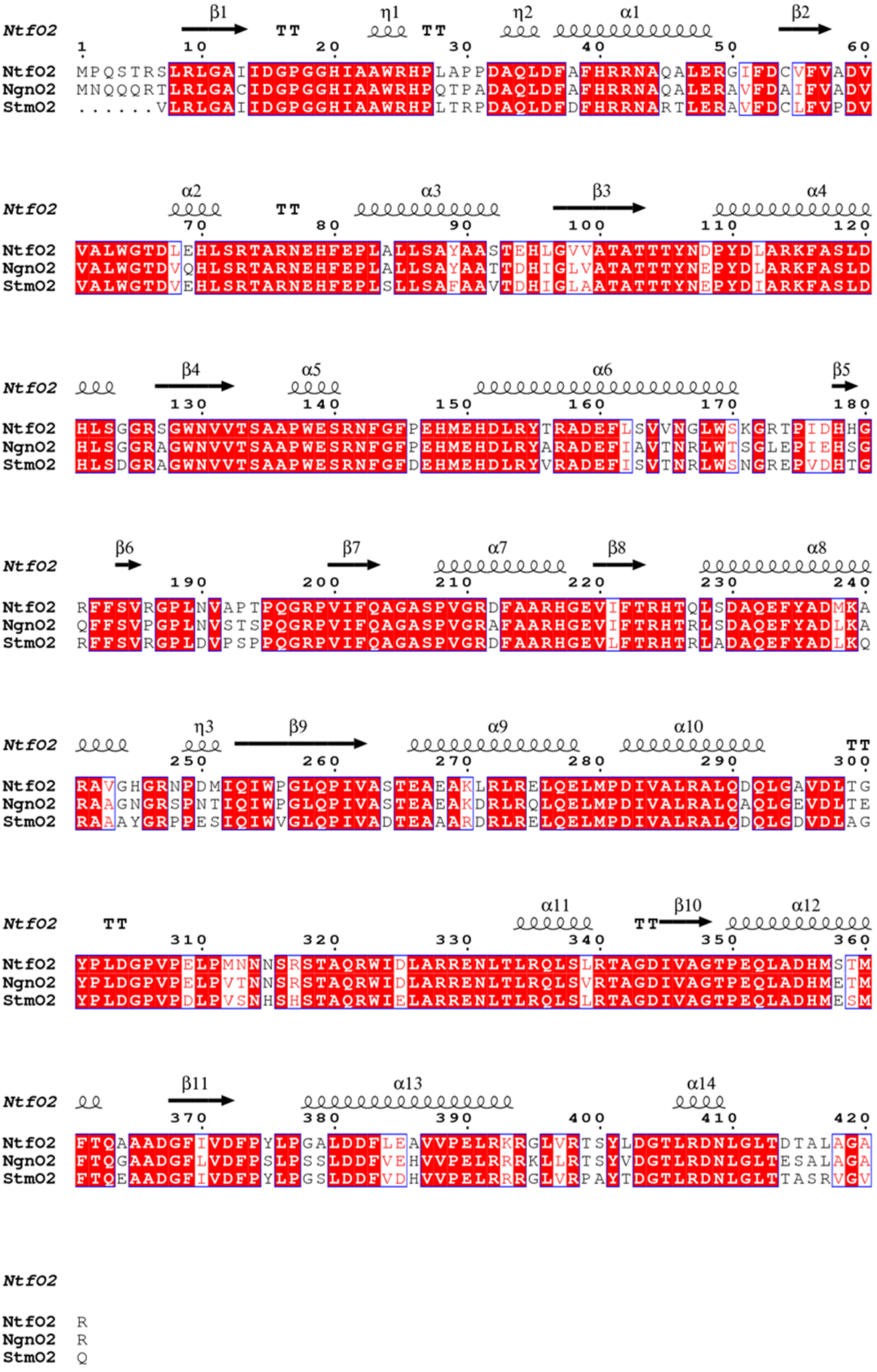

Supplementary Fig. 12. Multiple amino acid sequences alignment of StmO2 and its homologues. The multiple alignment was generated by MUSCLE[7] and rendered with ESPript 3.0[8]. The NCBI accession number of sequences NtfO2, StmO2 and NgnO2 are WP_040742799.1, AWH12915.1, and AXG22410.1, respectively.

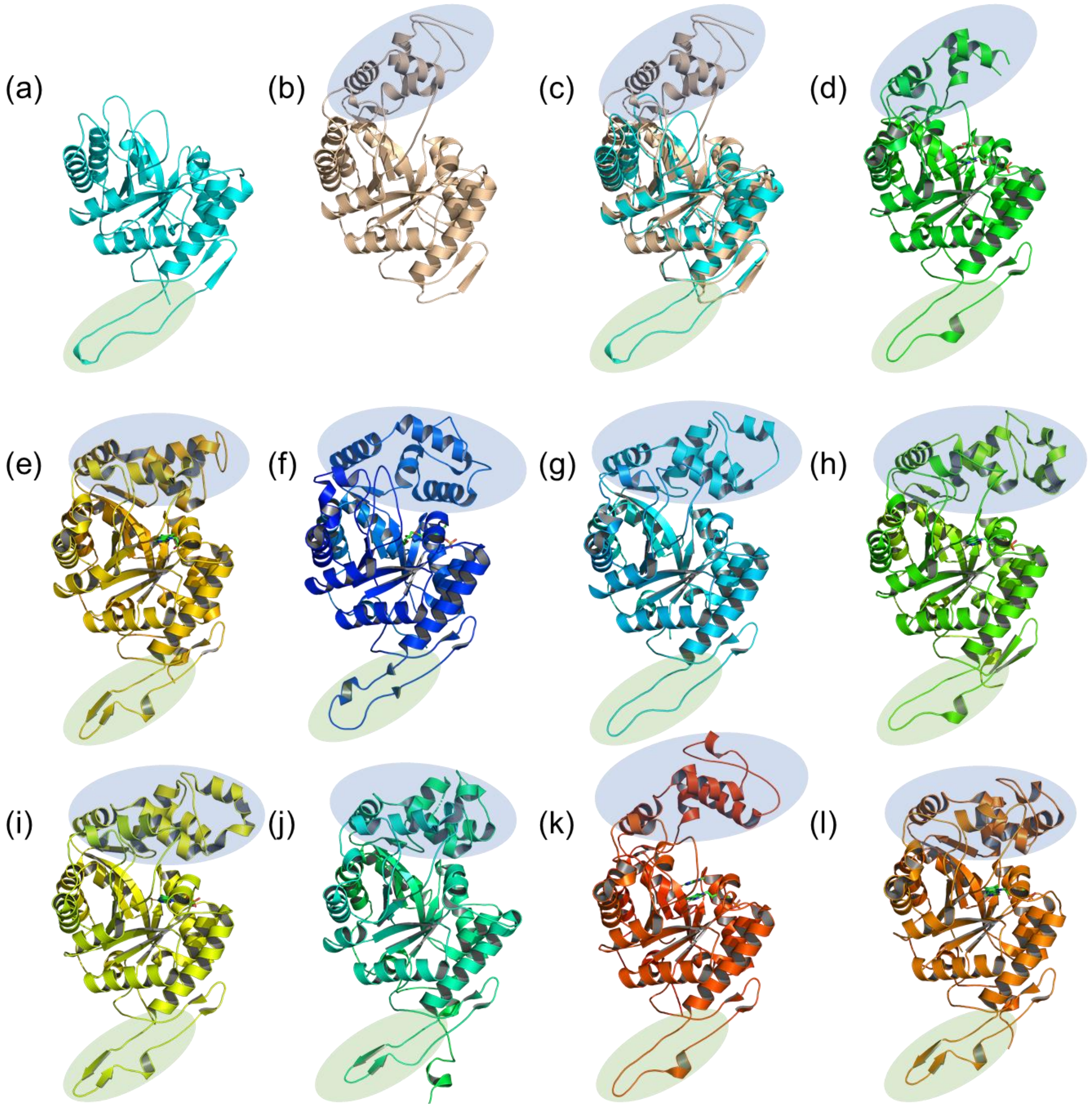

Supplementary Fig. 13. Monomeric crystal structures of the Luciferase-like monooxygenase family. (a) Crystal structure of NtfO1, (b) Crystal structure of NtfO2, (c) The superimposed image of NtfO1 and NtfO2, (d) Crystal structure of Ytnj, a protein of unknown function from *Bacillus subtilis* (1YW1) with a FMN and a glucose in the active site, (e) Crystal structure of long-chain alkane monooxygenase (LadA) from *Geobacillus thermodenitrificans* (3B9O) with a FMN in the active site, (f) Crystal structure of EDTA monooxygenase (EmoA) from *Chelativorans* sp. BNC1 (5DQP), (g) Crystal structure of dibenzothiophene sulfone monooxygenase (BdsA) from *Bacillus subtilis* WU-S2B (5TLC), (h) Crystal structure of Riboflavin Lyase (RcaE) with FMN (5W4Y), (i) Crystal structure of dibenzothiophene sulfone monooxygenase (BdsA) in complex with FMN (5XKD), (j) Crystal structure of the dimethylsulfide monooxygenase (DmoA) from *Hyphomicrobium sulfonivorans* (6AK1), (k) Crystal structure of flavin monooxygenase (CmoJ) from *Bacillus subtilis* subsp. 168 bound with FMN (6ASL), (l) Crystal structure of hexachlorobenzene monooxygenase (HcbA1) from *Nocardioides* sp. strain PD653 complexed with FMN (6LR5). The blue shade ellipse indicates top lid, while the green shade ellipse indicates the side loop tail.

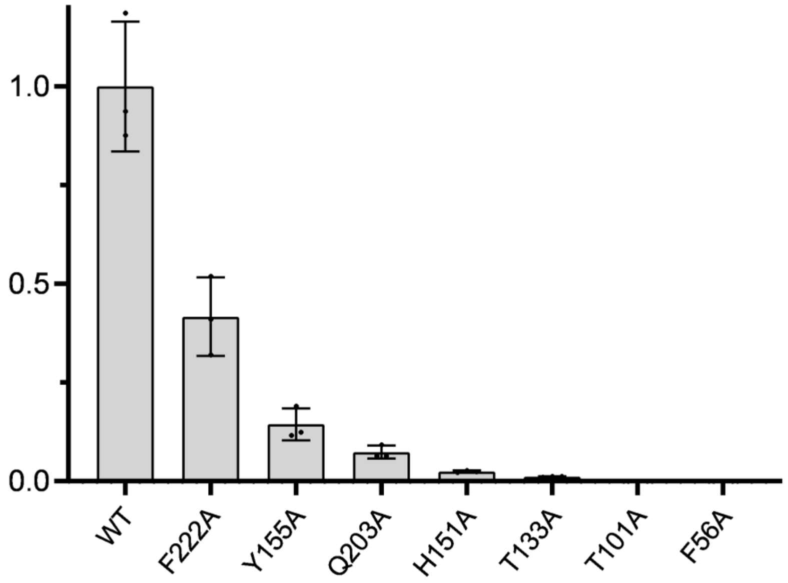

Supplementary Fig. 14. Relative enzymatic activity of NtfO1-NtfO2 and site-specific mutants. Data are presented as mean values +/- SD and n = 3 biologically independent samples were measured. Source data are provided as a Source Data file.

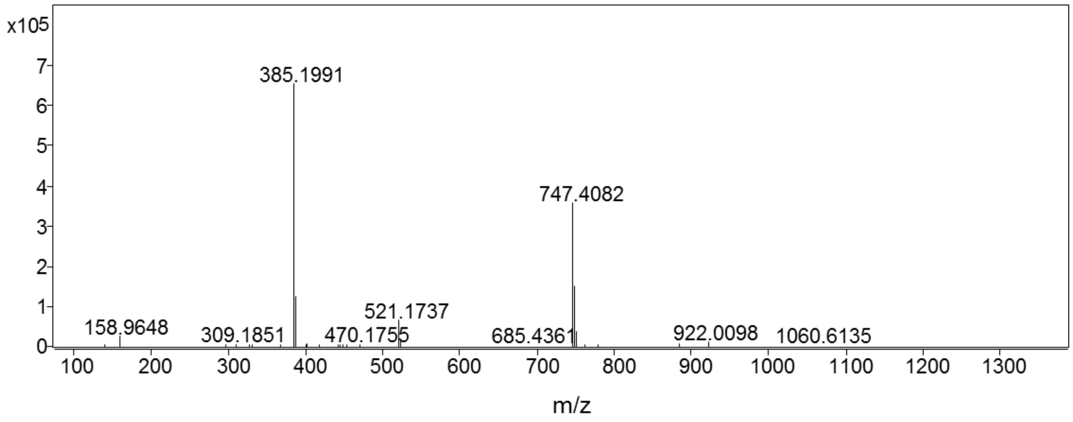

Supplementary Fig. 15. HR-ESIMS of spectrum of 8.

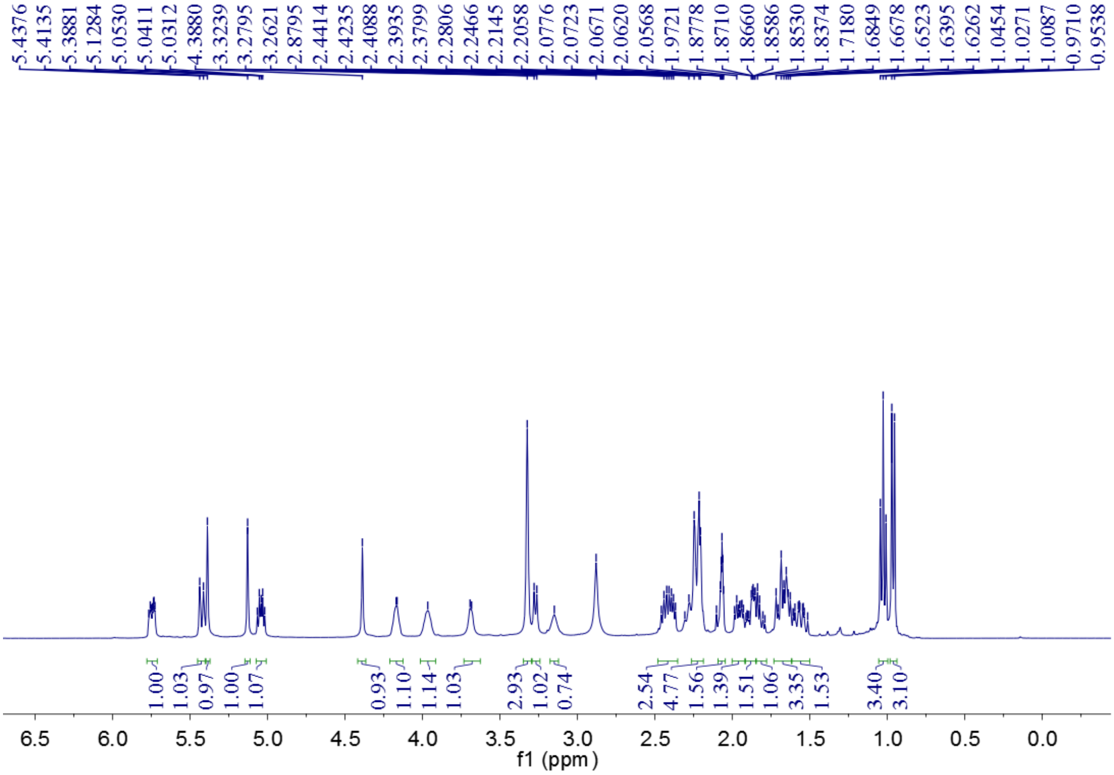

Supplementary Fig. 16. ^{1}**H NMR spectrum of 8 in acetone-*d*$_6$ (400 MHz).**

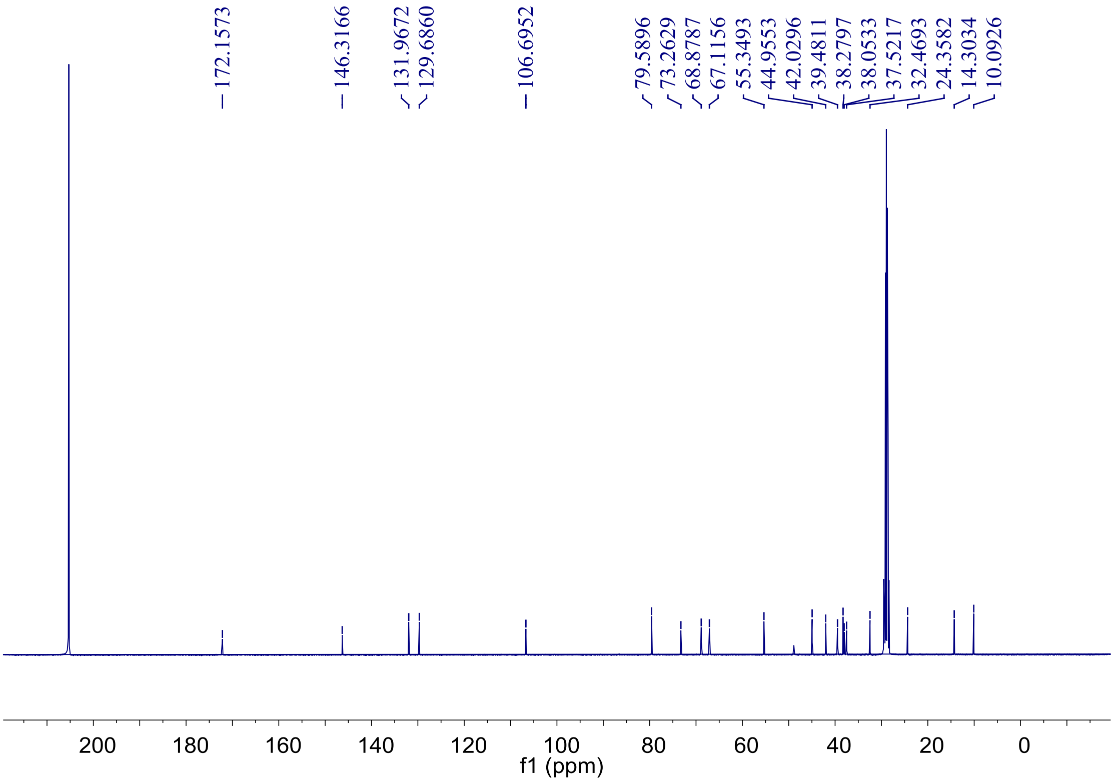

Supplementary Fig. 17. ^{13}**C NMR spectrum of 8 in acetone-*d*$_6$ (100 MHz).**

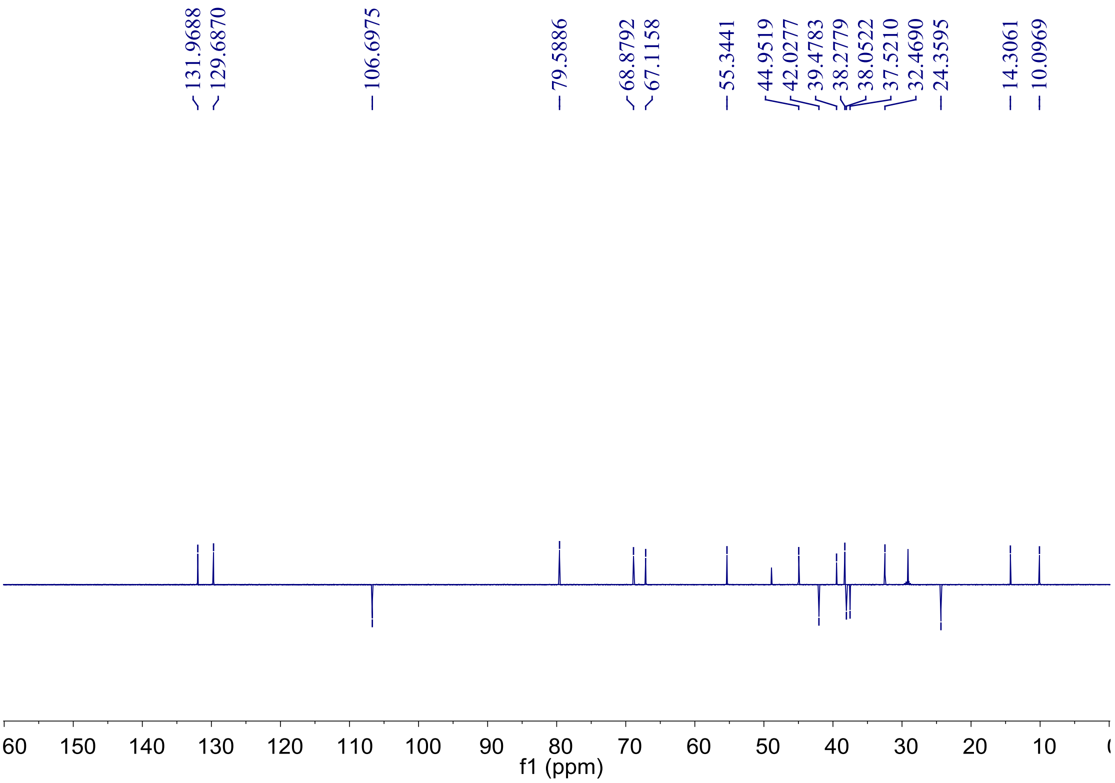

Supplementary Fig. 18. DEPT-135 NMR spectrum of 8 in acetone-d_6 (100 MHz).

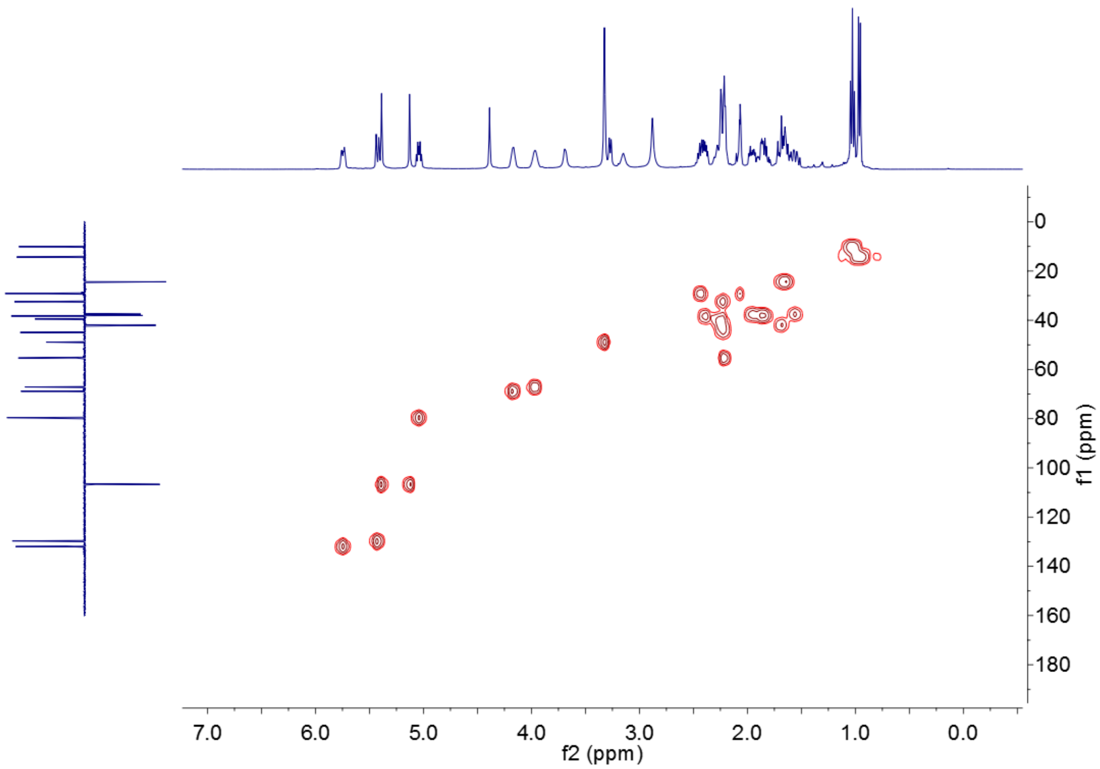

Supplementary Fig. 19. HSQC NMR spectrum of 8 in acetone-d_6 (400 MHz).

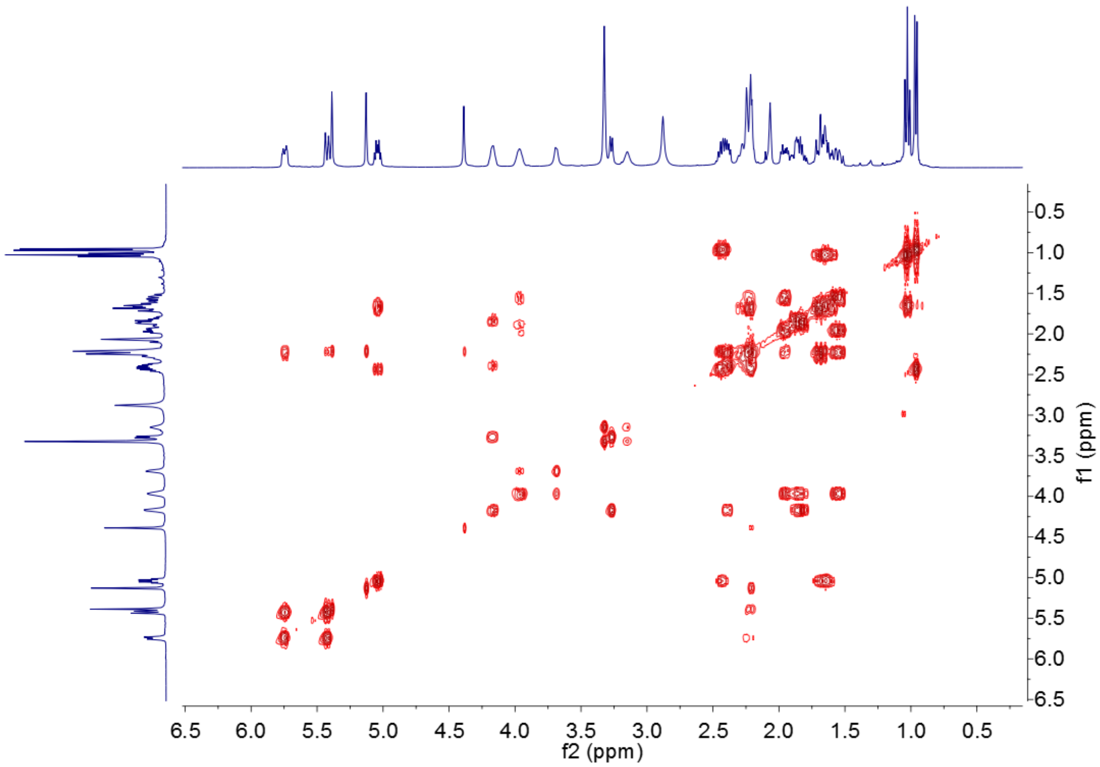

Supplementary Fig. 20. ^{1}H-^{1}H **COSY NMR spectrum of 8 in acetone-*d*$_6$ (400 MHz).**

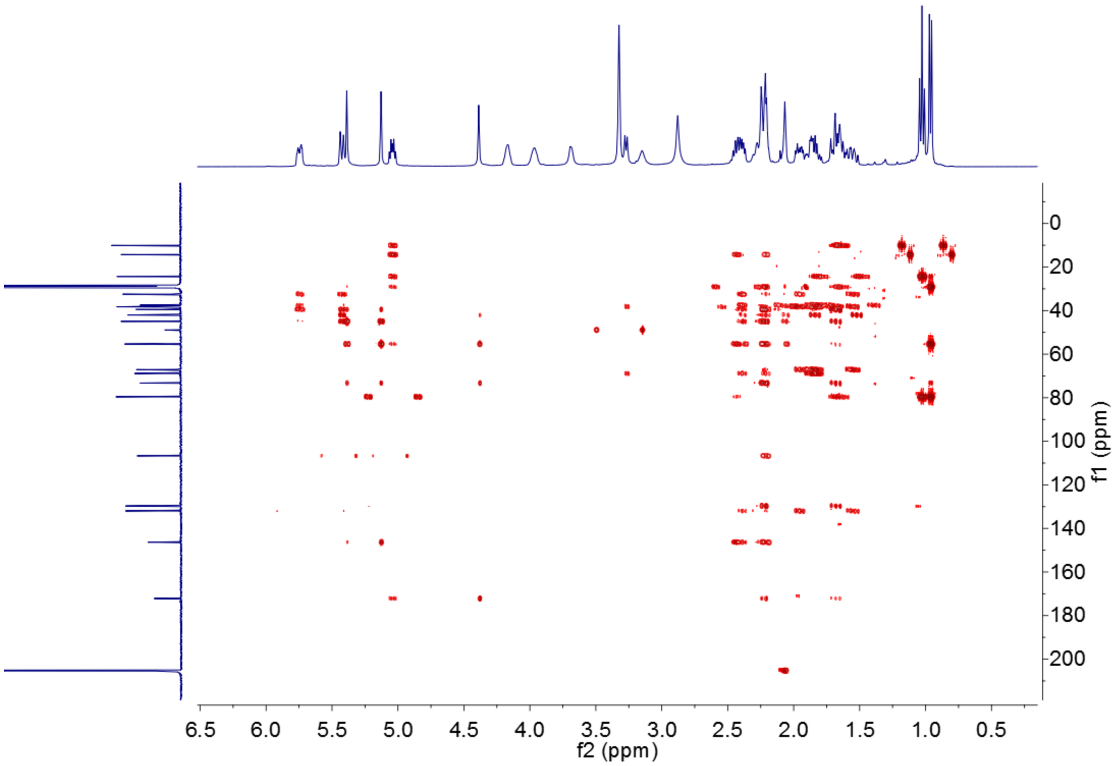

Supplementary Fig. 21. HMBC NMR spectrum of 8 in acetone-*d*$_6$ (400 MHz).

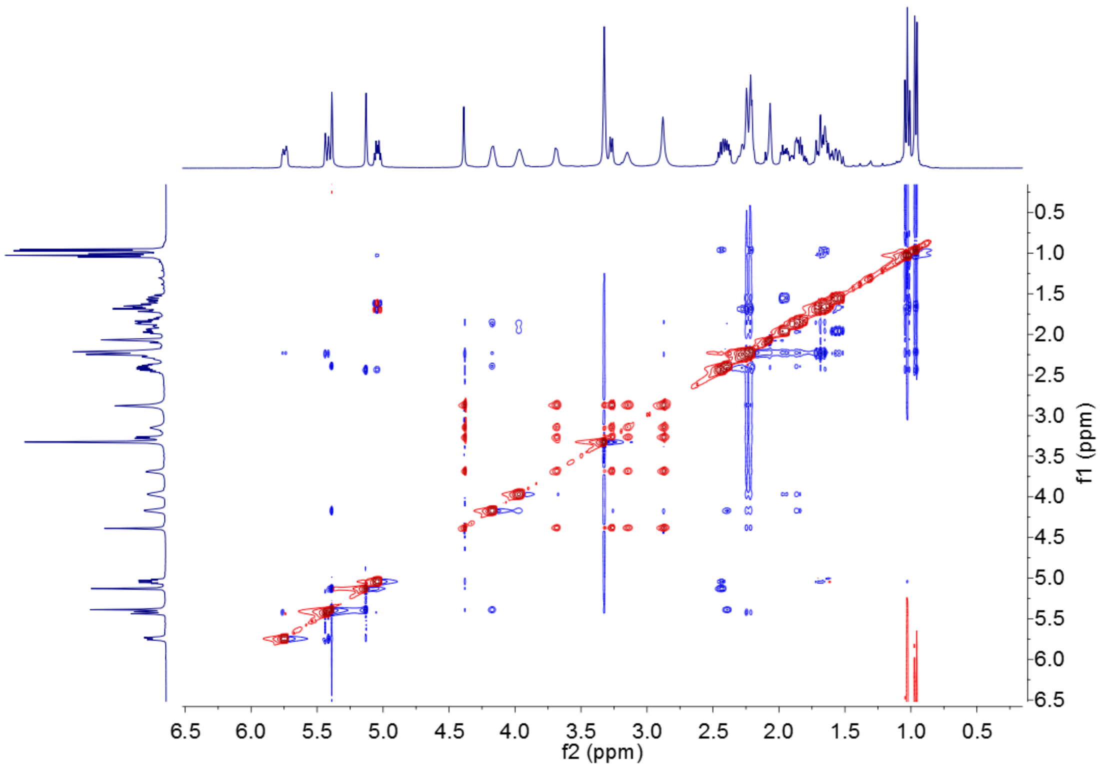

Supplementary Fig. 22. NOESY spectrum of 8 in acetone-*d*$_6$ (600 MHz).

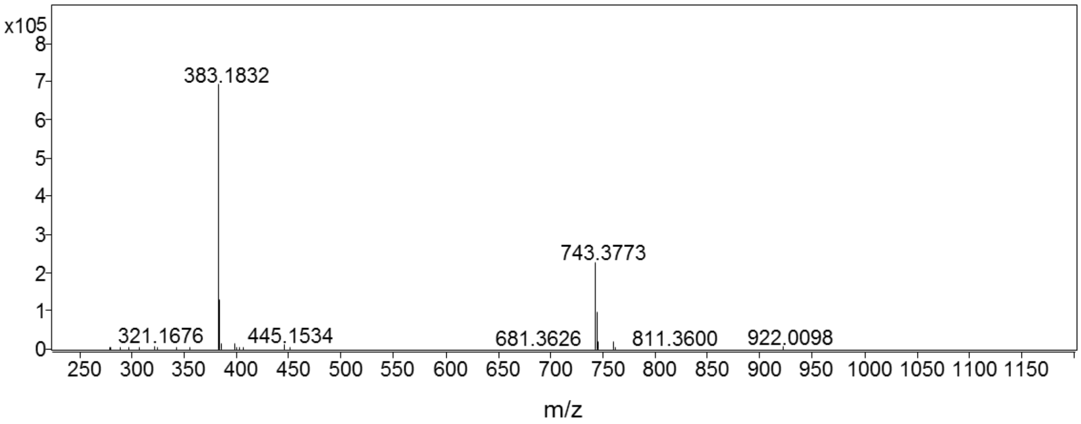

Supplementary Fig. 23. HR-ESIMS spectrum of 9.

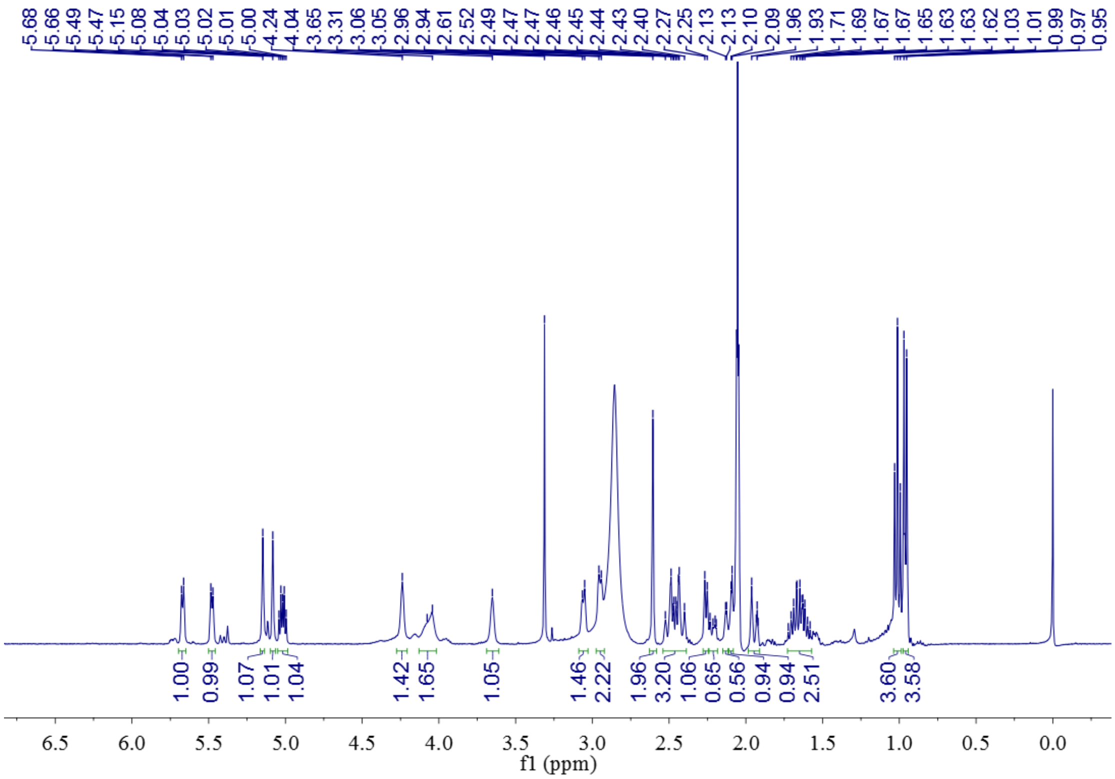

Supplementary Fig. 24. **^{1}H NMR spectrum of 9 in acetone-*d*6 (400 MHz).**

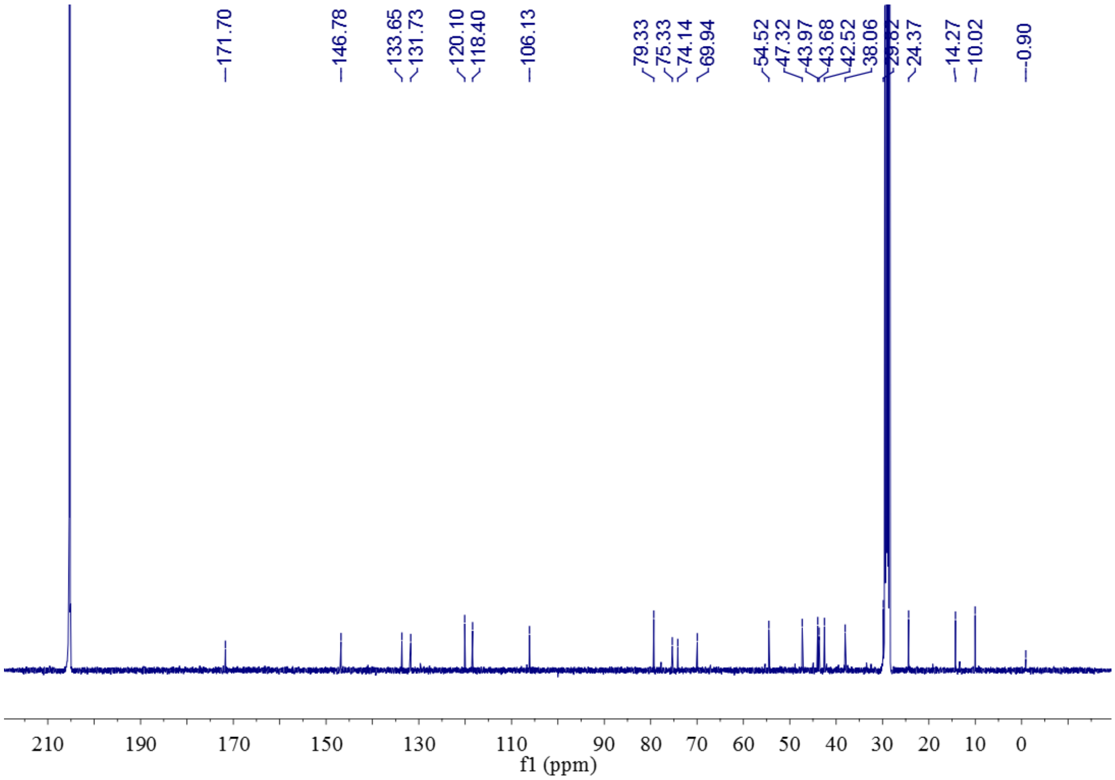

Supplementary Fig. 25. ^{13}C NMR spectrum of 9 in acetone-*d*6 (100 MHz).

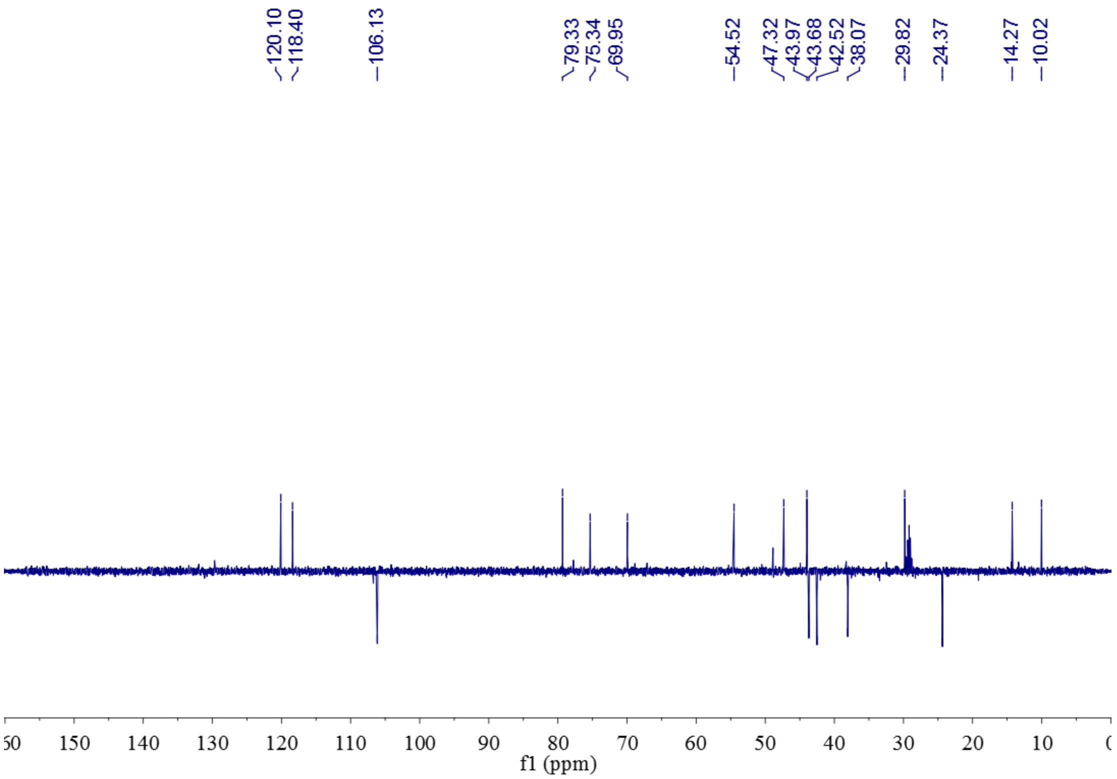

Supplementary Fig. 26. DEPT-135 NMR spectrum of 9 in acetone-d_6 (100 MHz).

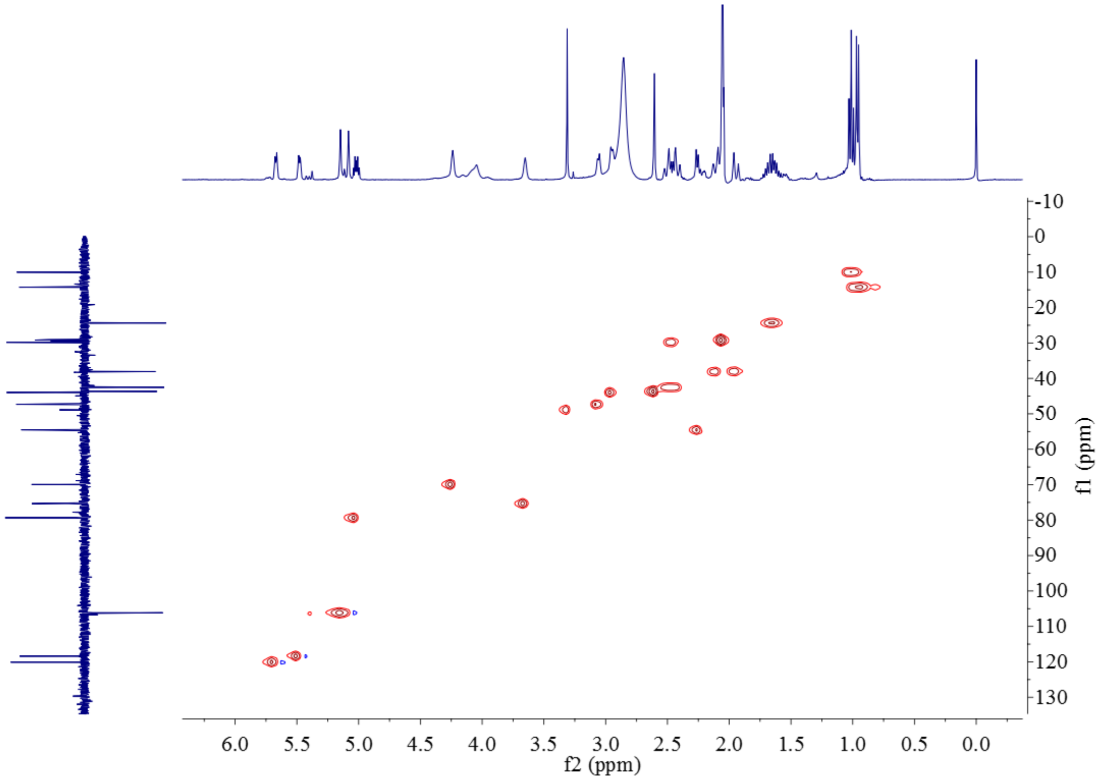

Supplementary Fig. 27. HSQC NMR spectrum of 9 in acetone-d_6 (400 MHz).

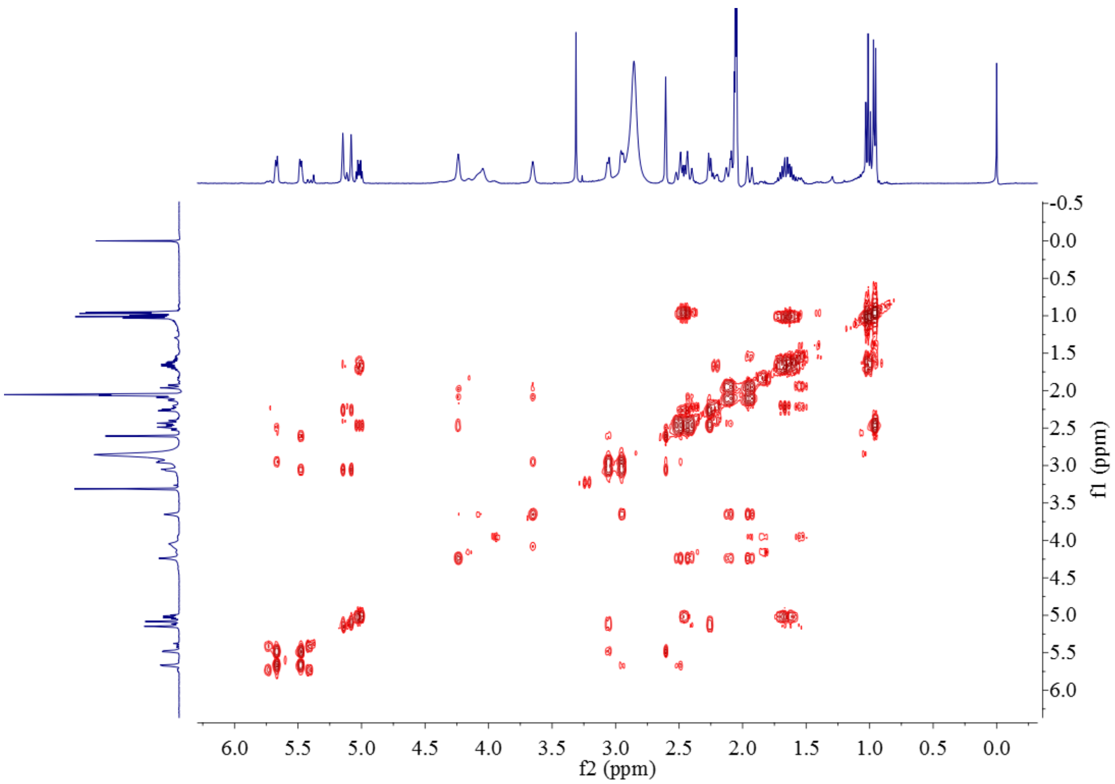

Supplementary Fig. 28. ^{1}H-^{1}H **COSY NMR spectrum of 9 in acetone-d_6 (400 MHz).**

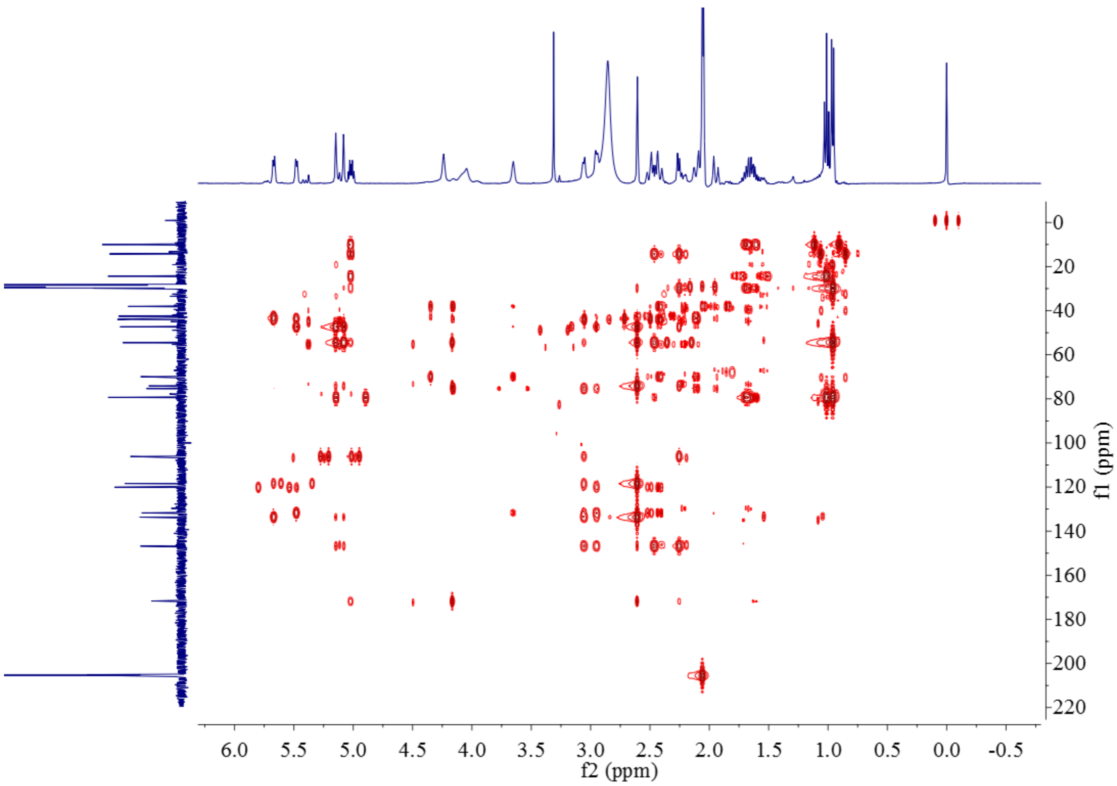

Supplementary Fig. 29. HMBC NMR spectrum of 9 in acetone-d_6 (600 MHz).

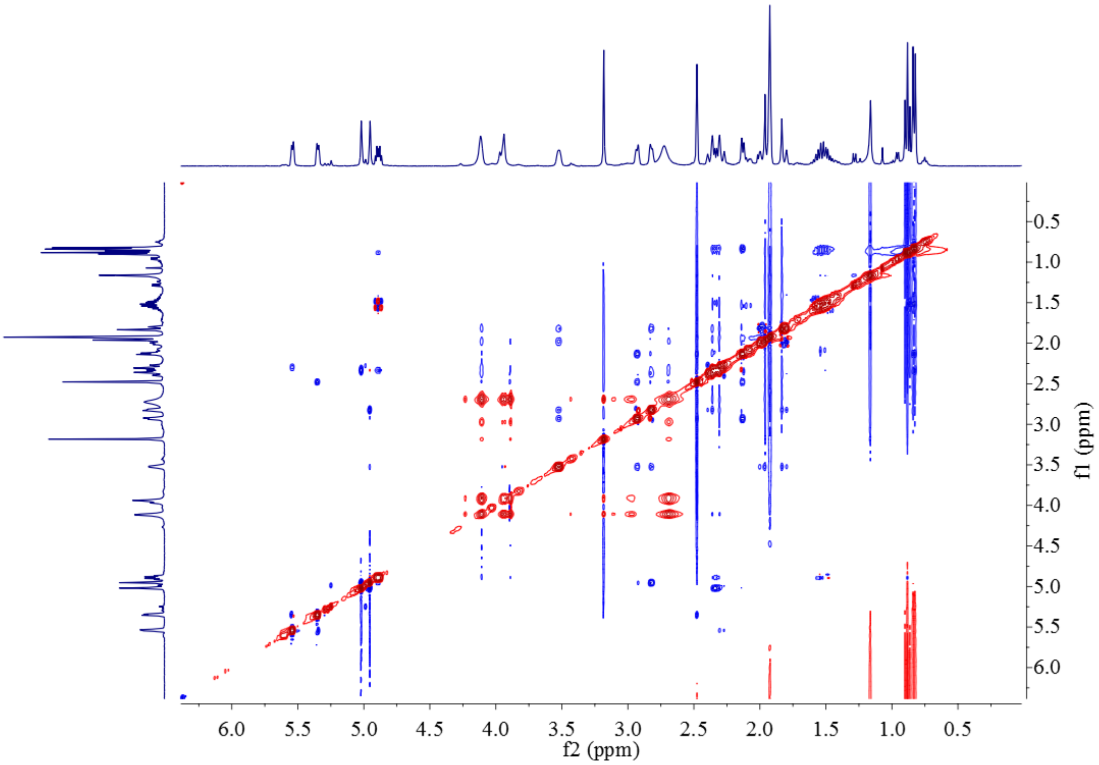

Supplementary Fig. 30. NOESY spectrum of 9 in acetone-d_6 (400 MHz).

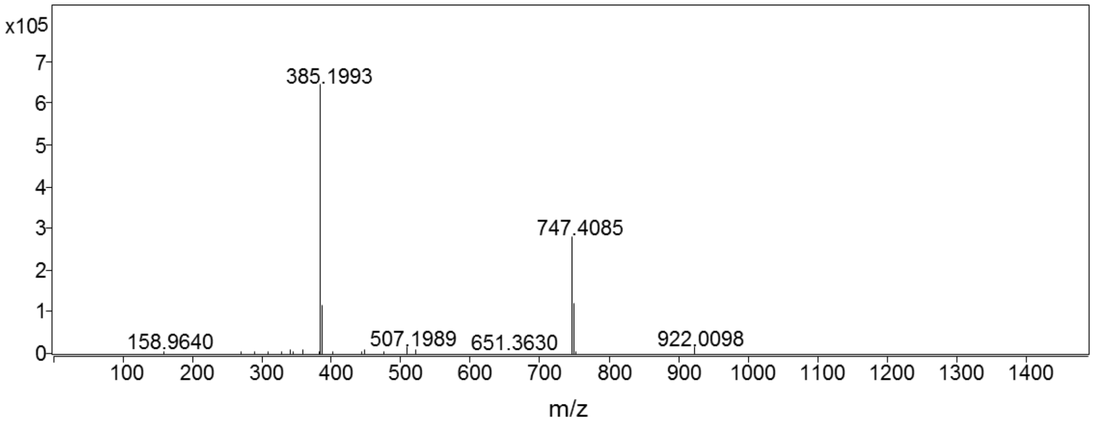

Supplementary Fig. 31. HR-ESIMS of spectrum of 10.

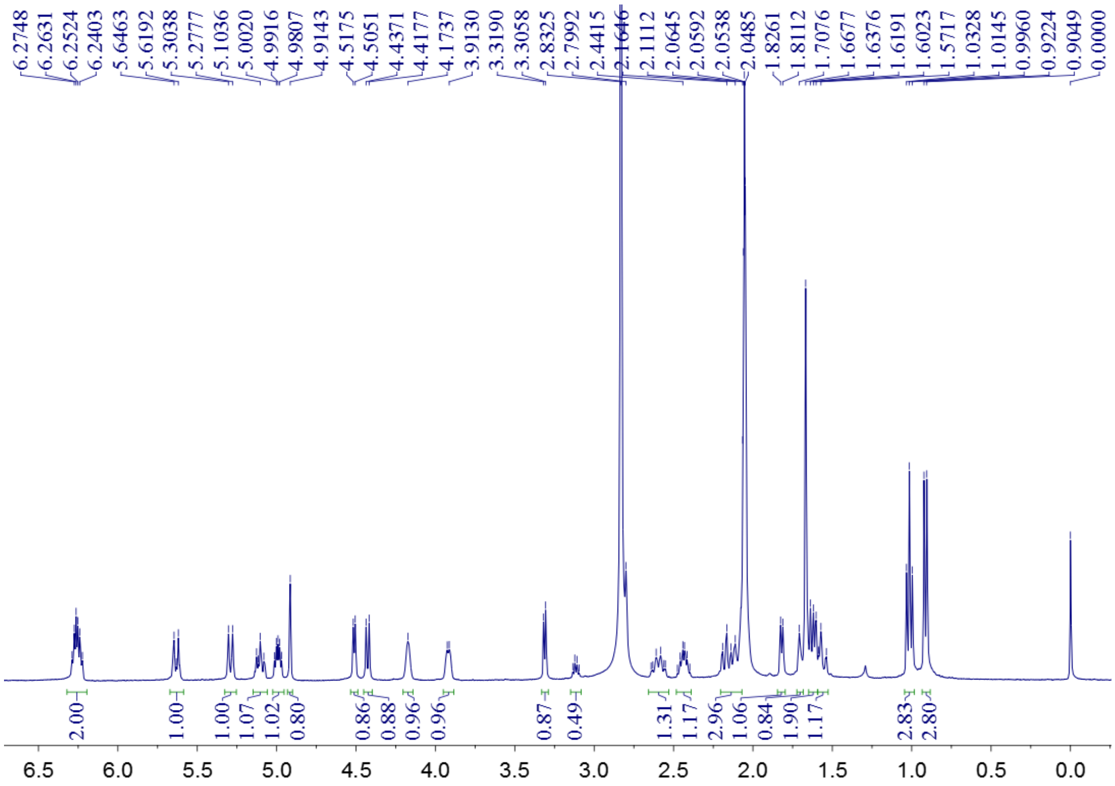

Supplementary Fig. 32. ^{1}H NMR spectrum of 10 in acetone-d_6 (400 MHz).

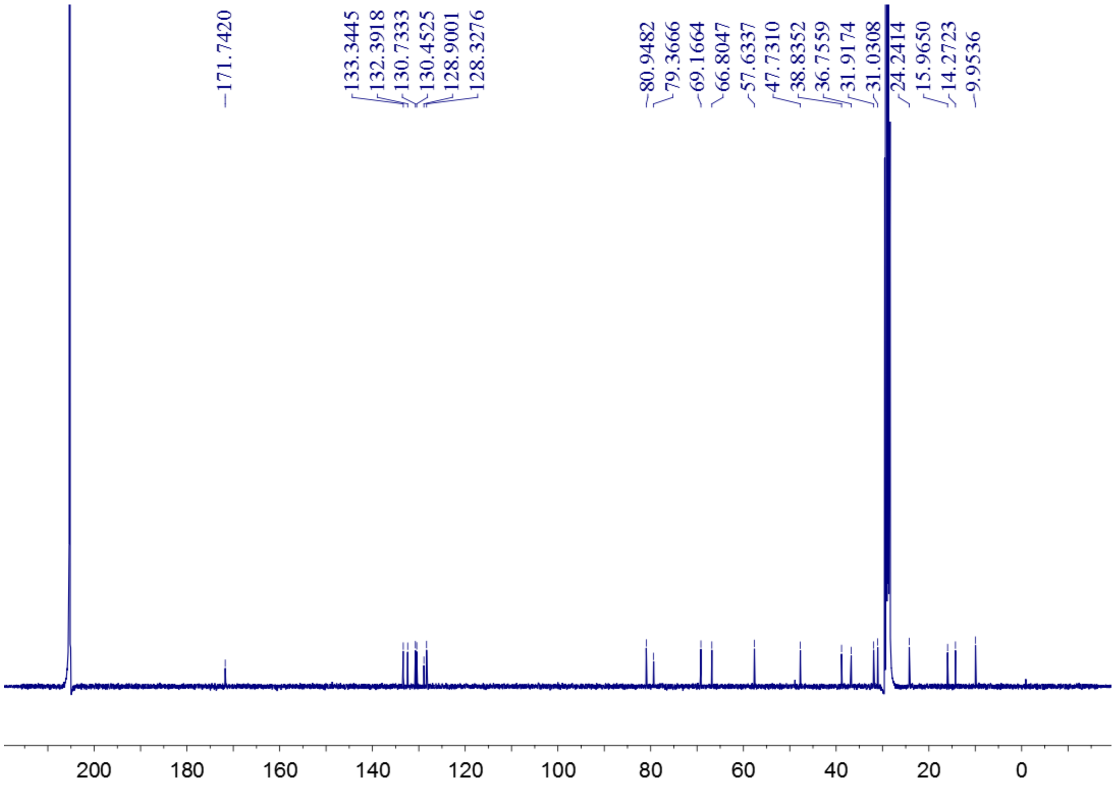

Supplementary Fig. 33. ^{13}C NMR spectrum of 10 in acetone-d_6 (100 MHz).

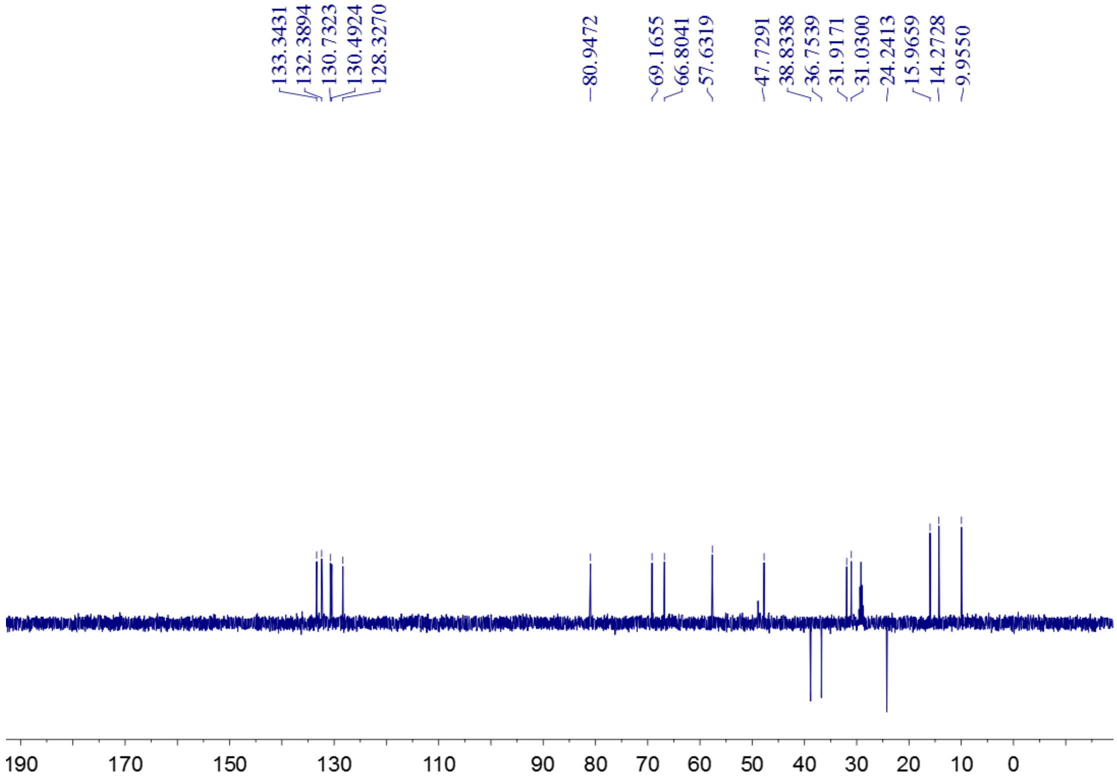

Supplementary Fig. 34. DEPT-135 NMR spectrum of 10 in acetone-d_6 (100 MHz).

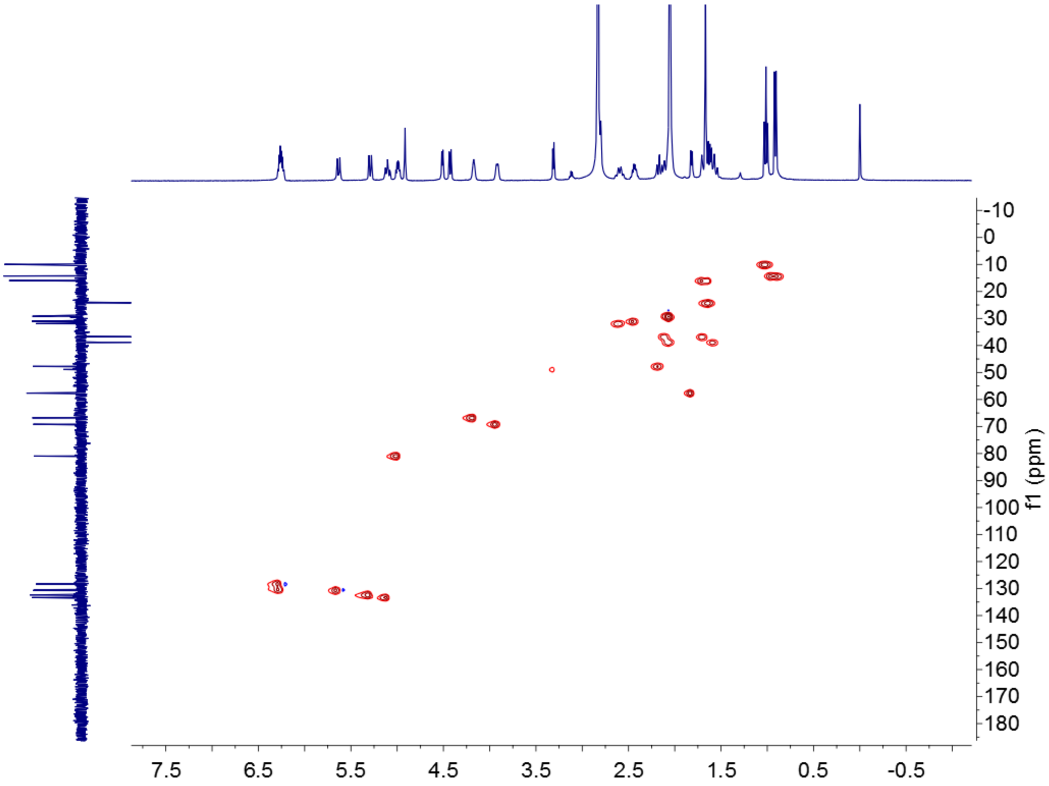

Supplementary Fig. 35. HSQC NMR spectrum of 10 in acetone-d_6 (100 MHz).

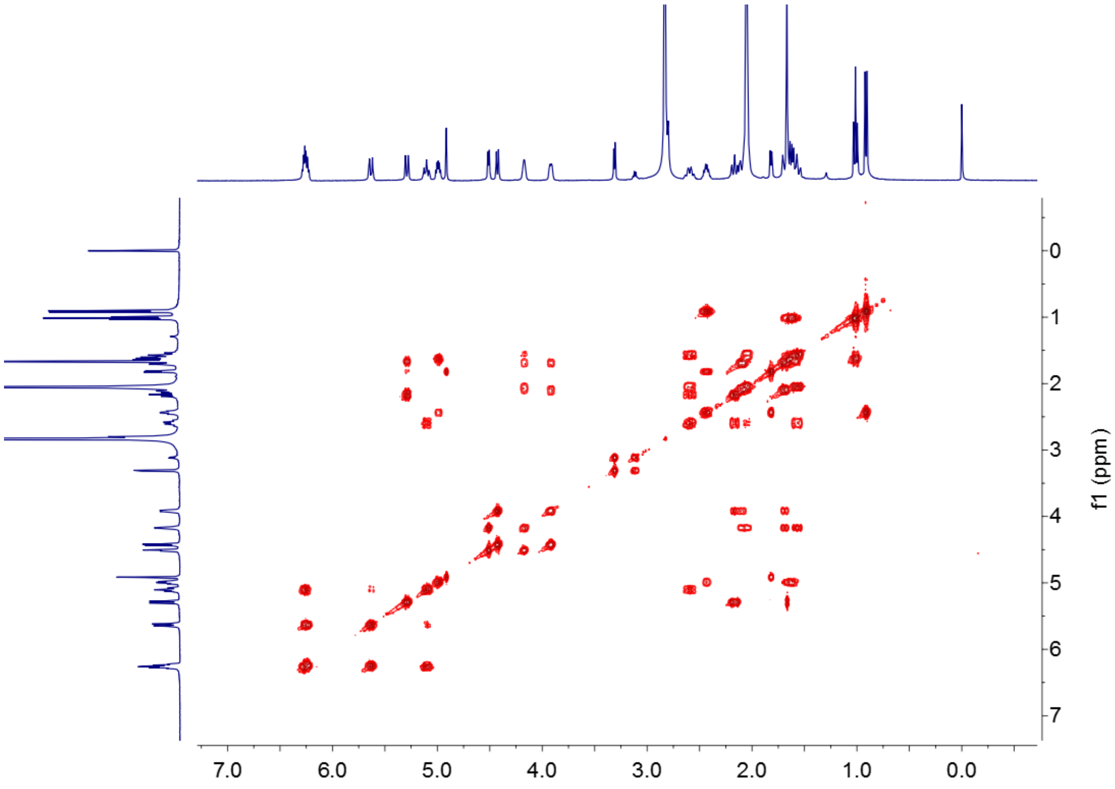

Supplementary Fig. 36. ^{1}H-^{1}H COSY NMR spectrum of 10 in acetone-d_6 (400 MHz).

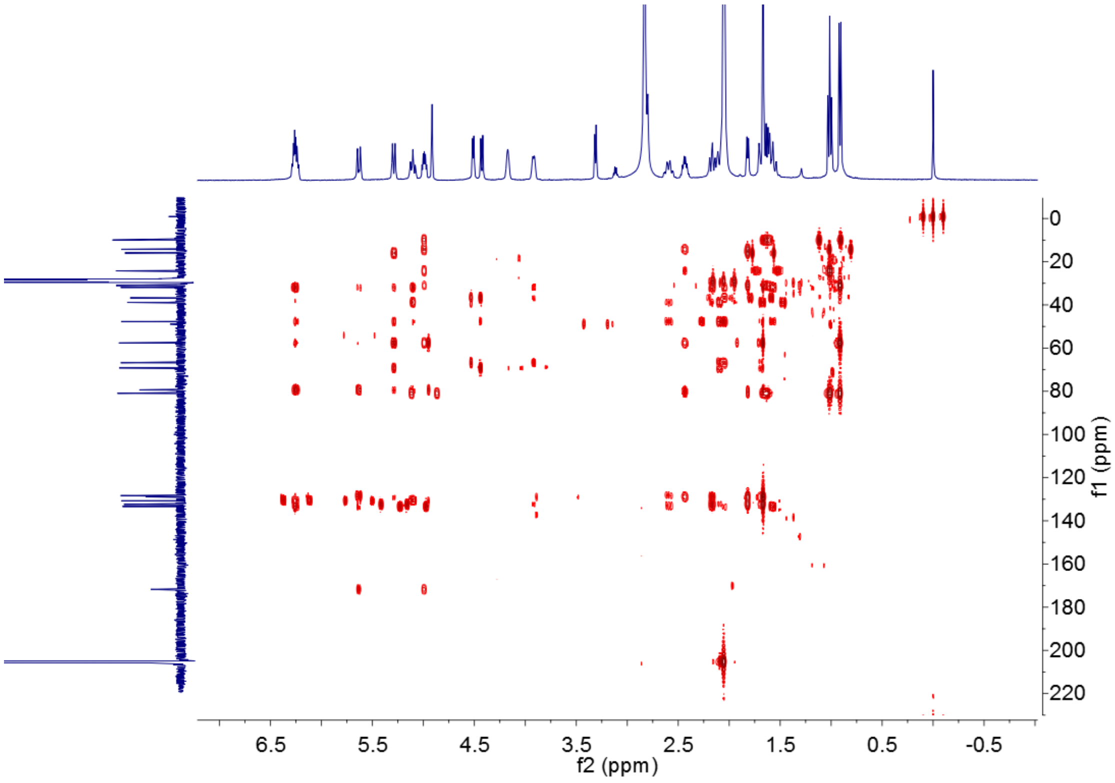

Supplementary Fig. 37. HMBC NMR spectrum of 10 in acetone-d_6 (600 MHz).

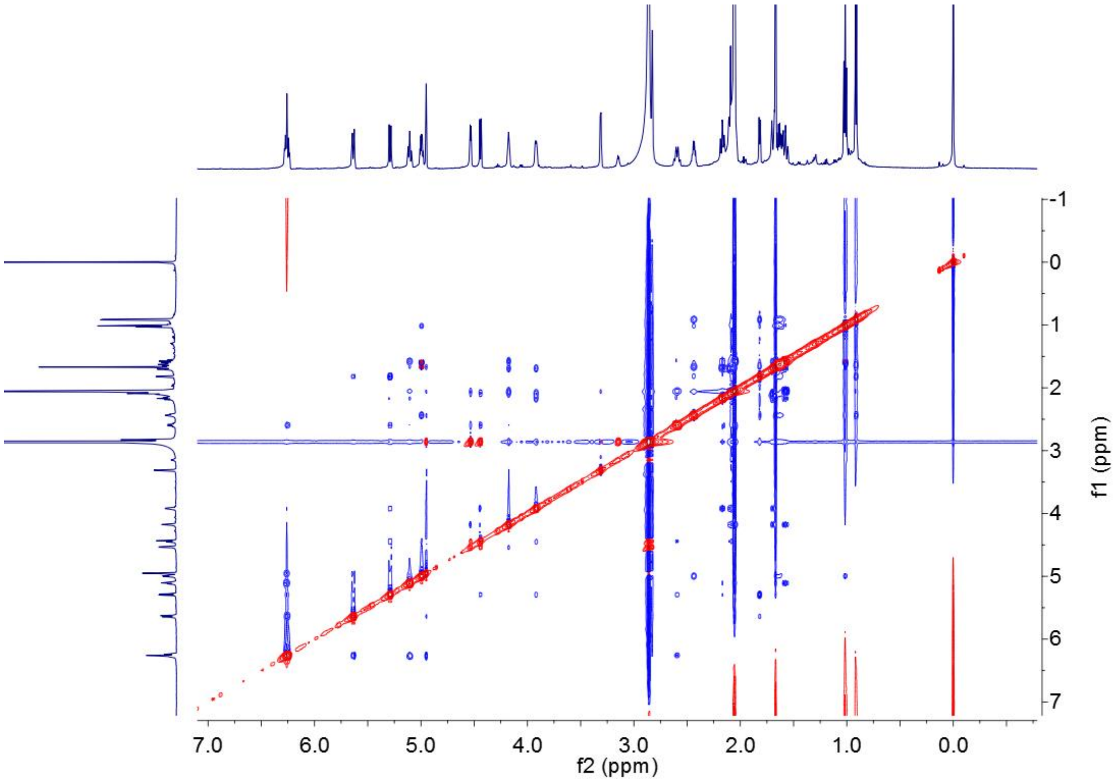

Supplementary Fig. 38. NOESY spectrum of 10 in acetone-d_6 (600 MHz).

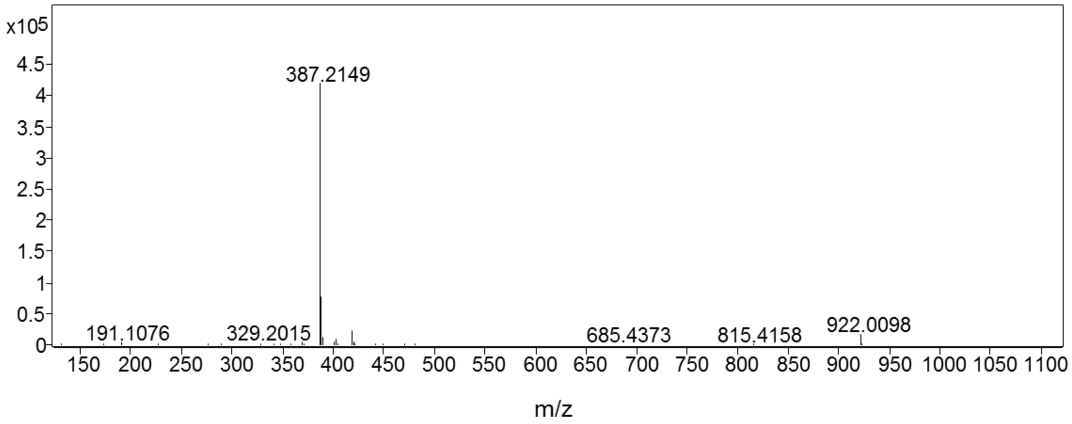

Supplementary Fig. 39. HR-ESIMS of spectrum of 11.

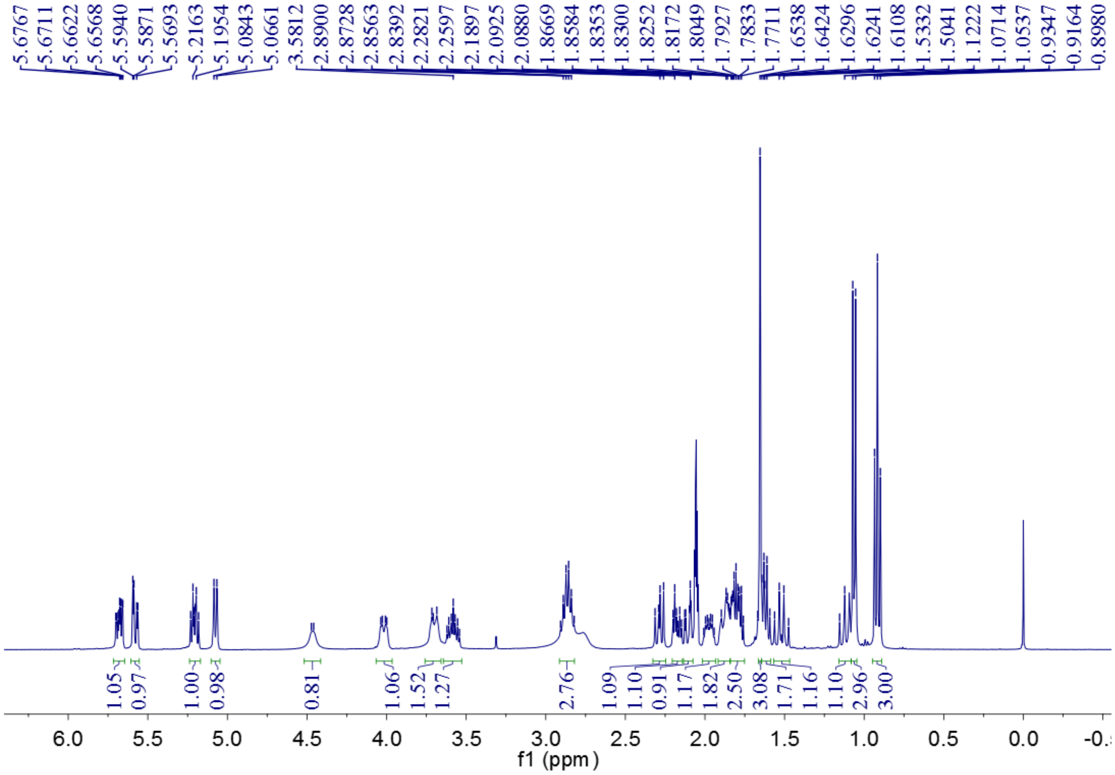

Supplementary Fig. 40. ^{1}H NMR spectrumof 11 in acetone-d_6 (400 MHz).

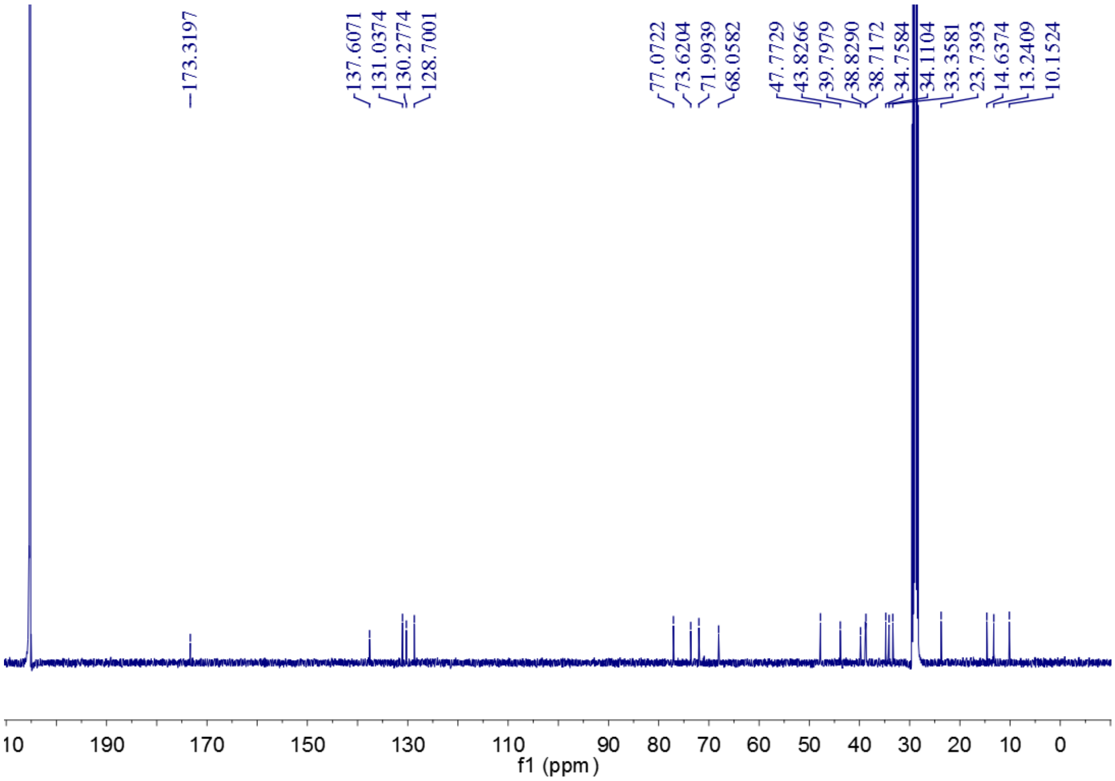

Supplementary Fig. 41. ^{13}C NMR spectrum of 11 in acetone-d_6 (100 MHz).

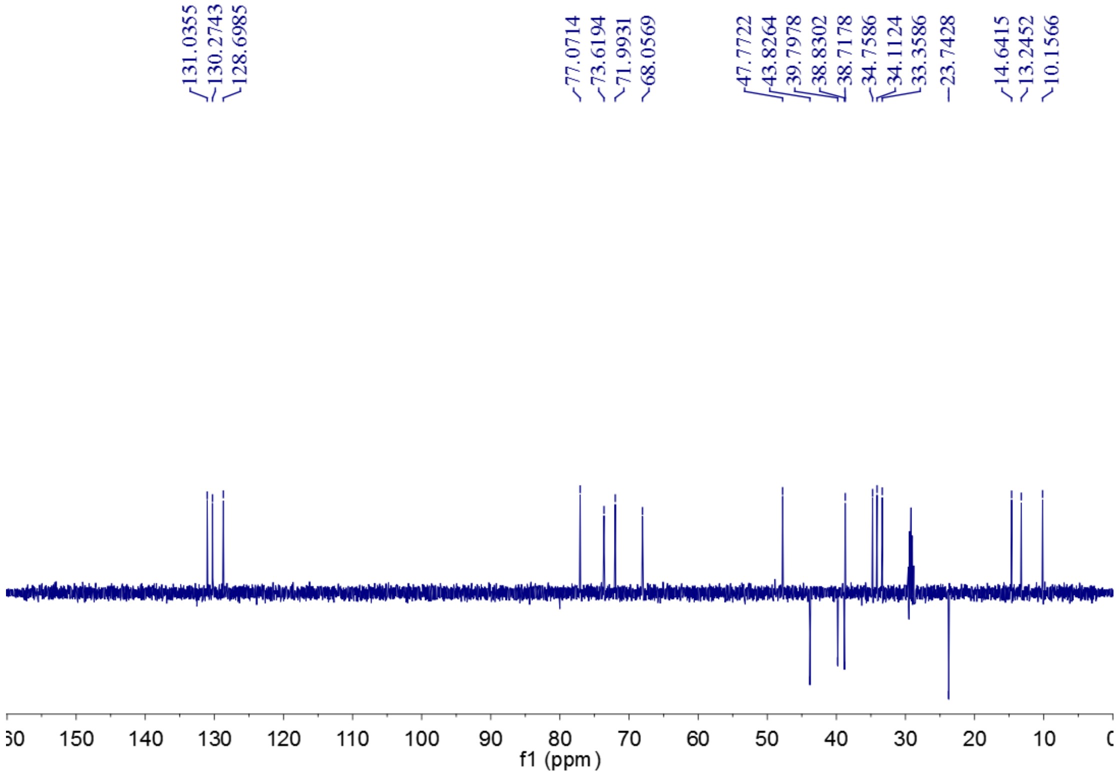

Supplementary Fig. 42. DEPT-135 NMR spectrum of 11 in acetone-d_6 (100 MHz).

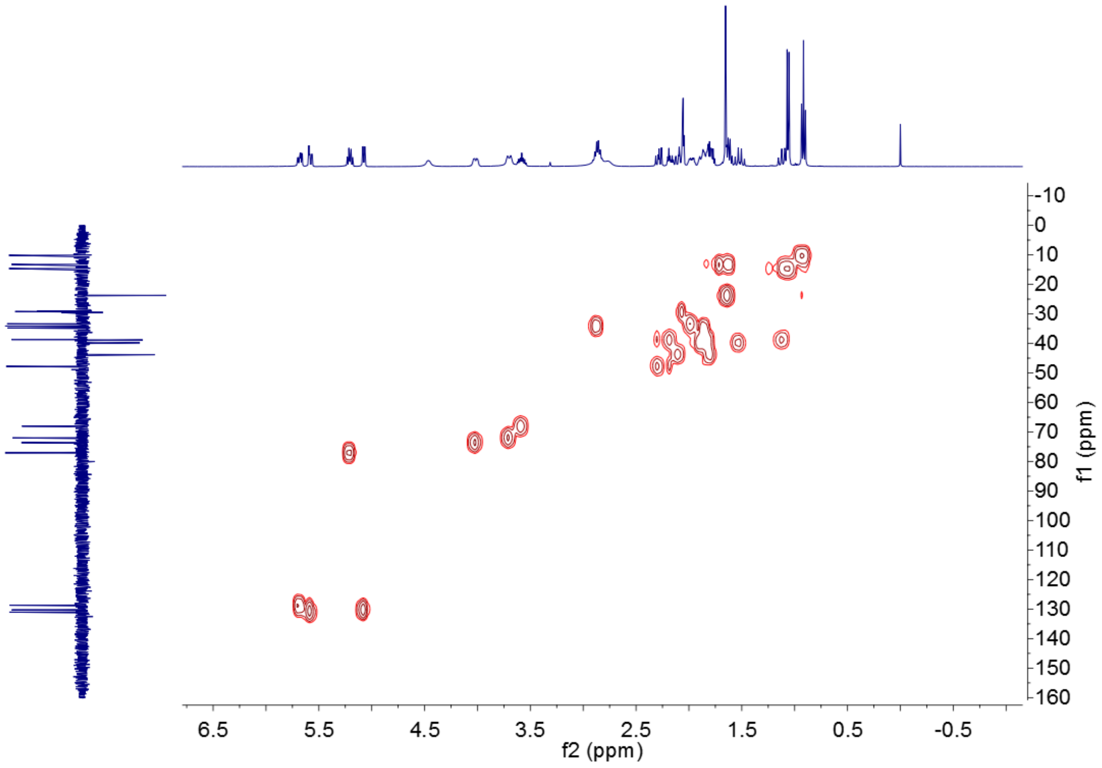

Supplementary Fig. 43. HSQC NMR spectrum of 11 in acetone-d_6 (400 MHz).

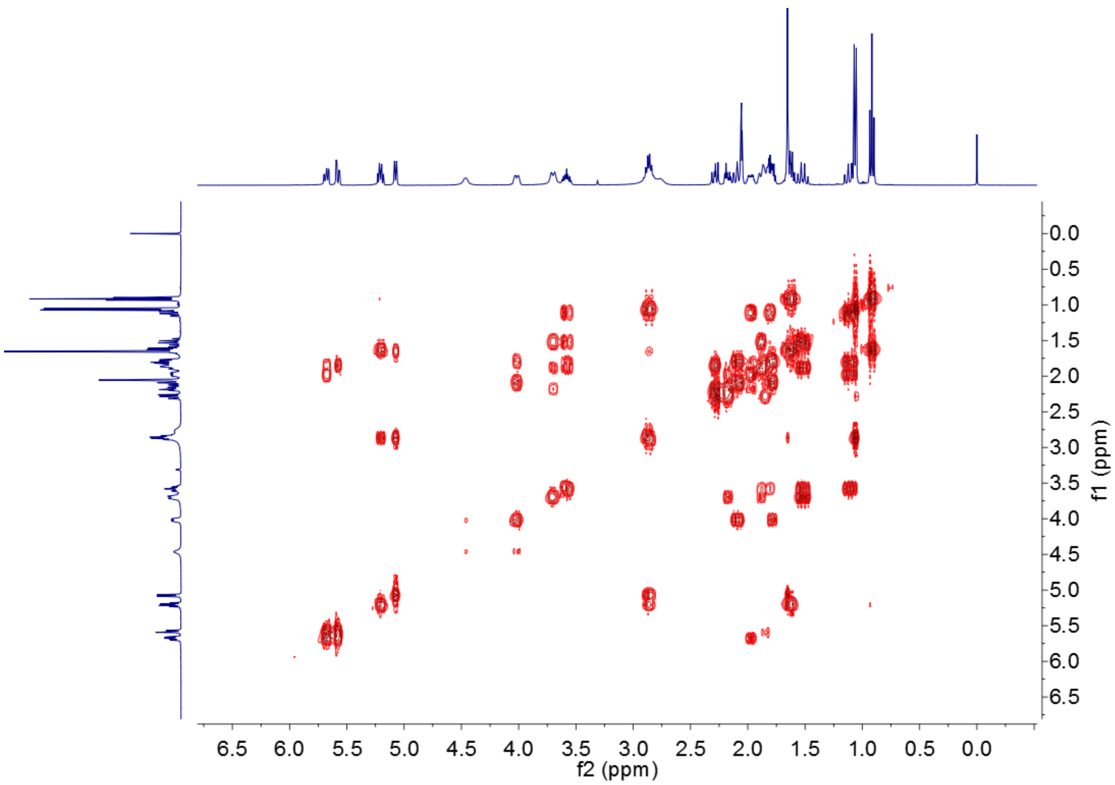

Supplementary Fig. 44. ^{1}H-^{1}H **COSY NMR spectrum of 11 in acetone-d_6 (400 MHz).**

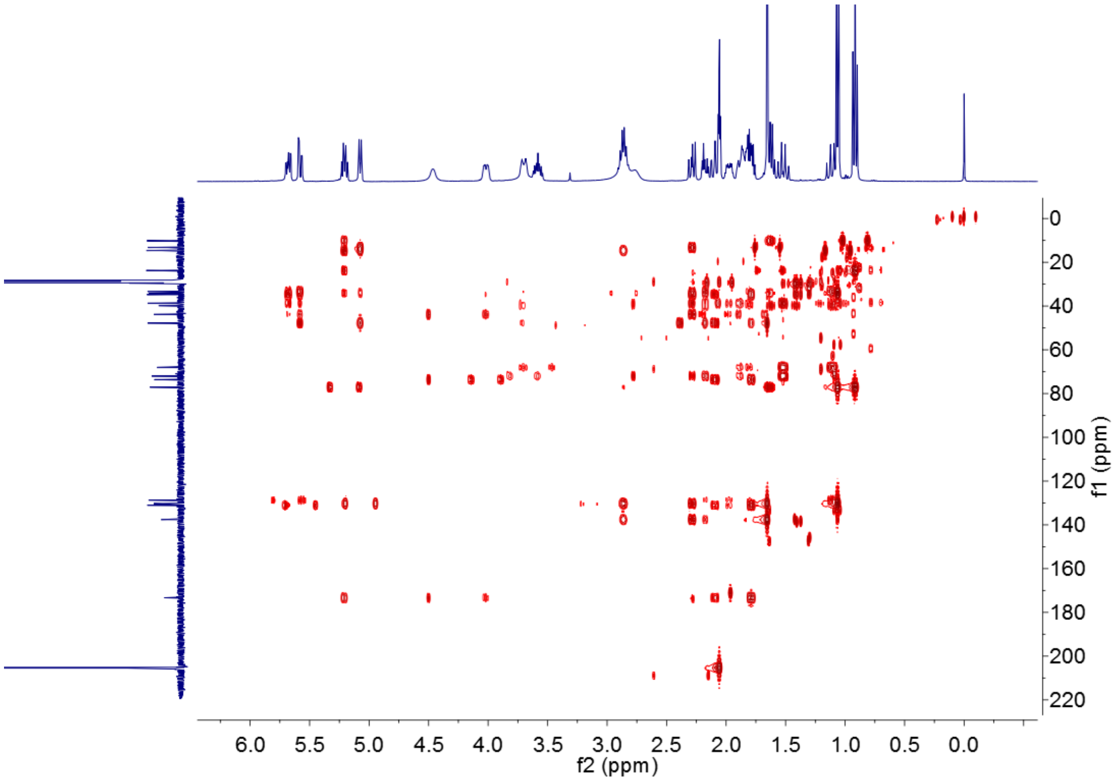

Supplementary Fig. 45. HMBC NMR spectrum of 11 in acetone-d_6 (600 MHz).

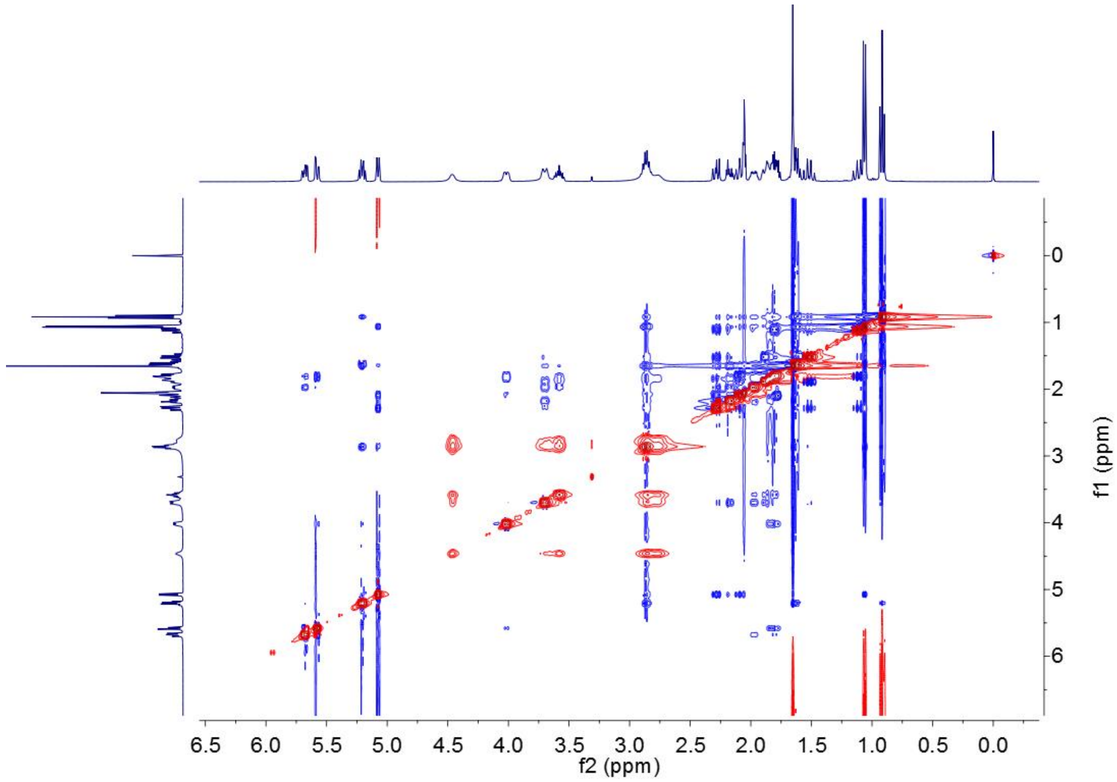

Supplementary Fig. 46. NOESY spectrum of 11 in acetone-d_6 (400 MHz).

Supplementary references

1. Bierman, M. *et al*. Plasmid cloning vectors for the conjugal transfer of DNA from *Escherichia coli* to *Streptomyces* spp. *Gene* **116**, 43-49 (1992).
2. Li, L. *et al*. Engineered jadomycin analogues with altered sugar moieties revealing JadS as a substrate flexible *O*-glycosyltransferase. *Appl. Microbiol. Biot.* **101**, 5291-5300 (2017).
3. Doumith, M. et al. Analysis of genes involved in 6-deoxyhexose biosynthesis and transfer in *Saccharopolyspora erythraea. Mol. Gen. Genet.* **264**, 477-485 (2000).
4. Green, M. R. & Sambrook, J. Molecular Cloning: A Laboratory Manual 4th ed. Cold Spring Harbor Laboratory Press, (2012).
5. Zhang, B. et al. Discovery, biosynthesis, and heterologous production of streptoseomycin, an anti-microaerophilic bacteria macrodilactone. *Org. lett.* **20**, 2967-2971 (2018).
6. Zhang, B. *et al.* Enzyme-catalysed [6+4] cycloadditions in the biosynthesis of natural products. *Nature* **568**, 122-126 (2019).
7. Edgar, R. C. MUSCLE: multiple sequence alignment with high accuracy and high throughput. *Nucleic Acids Res.* **32**, 1792-1797 (2004).
8. Robert, X. & Gouet, P. Deciphering key features in protein structures with the new ENDscript server. *Nucl. Acids Res.* **42**, W320-W324 (2014).
