# Supplementary material for: A [6+4]-cycloaddition adduct is the biosynthetic intermediate in streptoseomycin biosynthesis
Source: Nat Commun. 2021 Apr 7;12:2092. doi: 10.1038/s41467-021-22395-7 (PMC8027225; doi:10.1038/s41467-021-22395-7)
Supplement: Supplementary file 3 — Description of Additional Supplementary Files [file 41467_2021_22395_MOESM3_ESM.pdf]

Description of additional supplementary files

Title: Supplementary Data 1

Description: DFT-computed energies and Cartesian coordinates.
