# Supplementary material for: A [6+4]-cycloaddition adduct is the biosynthetic intermediate in streptoseomycin biosynthesis
Source: Nat Commun. 2021 Apr 7;12:2092. doi: 10.1038/s41467-021-22395-7 (PMC8027225; doi:10.1038/s41467-021-22395-7)
Supplement: Supplementary file 4 — Reporting Summary [file 41467_2021_22395_MOESM4_ESM.pdf]

nature research

Corresponding author(s): Hui Ming Ge
Last updated by author(s): Mar 6, 2021

Reporting Summary

Nature Research wishes to improve the reproducibility of the work that we publish. This form provides structure for consistency and transparency in reporting. For further information on Nature Research policies, see our Editorial Policies and the Editorial Policy Checklist.

Statistics

For all statistical analyses, confirm that the following items are present in the figure legend, table legend, main text, or Methods section.

n/a	Confirmed	
☐	☒	The exact sample size (*n*) for each experimental group/condition, given as a discrete number and unit of measurement
☐	☒	A statement on whether measurements were taken from distinct samples or whether the same sample was measured repeatedly
☒	☐	The statistical test(s) used AND whether they are one- or two-sided<br>*Only common tests should be described solely by name; describe more complex techniques in the Methods section.*
☒	☐	A description of all covariates tested
☒	☐	A description of any assumptions or corrections, such as tests of normality and adjustment for multiple comparisons
☐	☒	A full description of the statistical parameters including central tendency (e.g. means) or other basic estimates (e.g. regression coefficient) AND variation (e.g. standard deviation) or associated estimates of uncertainty (e.g. confidence intervals)
☒	☐	For null hypothesis testing, the test statistic (e.g. *F*, *t*, *r*) with confidence intervals, effect sizes, degrees of freedom and *P* value noted<br>*Give P values as exact values whenever suitable.*
☒	☐	For Bayesian analysis, information on the choice of priors and Markov chain Monte Carlo settings
☒	☐	For hierarchical and complex designs, identification of the appropriate level for tests and full reporting of outcomes
☒	☐	Estimates of effect sizes (e.g. Cohen's *d*, Pearson's *r*), indicating how they were calculated

Our web collection on statistics for biologists contains articles on many of the points above.

Software and code

Policy information about availability of computer code

Data collection	The crystallography data were collected at 17U beam line of SSRF. SDS-PAGE gels and agarose gels were detected on Bio-Rad Image Lab software (V5.2). HPLC data were collected by Agilent OpenLAB ChemStation C.01.07 and LC-MS data were collected by Agilent MassHunter Workstation software LC/MS data Acquisition B.06.01. The SEC data were collected by UNICORN 7.0.0.953.
Data analysis	Gaussian 09 program, imosfilm, AutoDock Vina 1_1_2, Phaser 2.7.0, PHENIX 1.14, PyMol 2.07, GaussView 6, GraphPad Prism 7.0, CCP4 7.0, Agilent Qualitative Analysis B.05.00, ExPASy ProtParam tool (https://web.expasy.org/protparam/), Excel 2013 (Microsoft), MAFFT version 7[illegible] Coot 0.8.9.1., Topspin 4.0.

For manuscripts utilizing custom algorithms or software that are central to the research but not yet described in published literature, software must be made available to editors and reviewers. We strongly encourage code deposition in a community repository (e.g. GitHub). See the Nature Research guidelines for submitting code & software for further information.

Data

Policy information about availability of data

All manuscripts must include a data availability statement. This statement should provide the following information, where applicable:
- Accession codes, unique identifiers, or web links for publicly available datasets
- A list of figures that have associated raw data
- A description of any restrictions on data availability

Atomic coordinates of NtfO1 and O2 has been deposited in the Protein Data Bank (PDB) with accession code 7E36.

April 2020

Field-specific reporting

Please select the one below that is the best fit for your research. If you are not sure, read the appropriate sections before making your selection.

☒ Life sciences ☐ Behavioural & social sciences ☐ Ecological, evolutionary & environmental sciences

For a reference copy of the document with all sections, see nature.com/documents/nr-reporting-summary-flat.pdf

Life sciences study design

All studies must disclose on these points even when the disclosure is negative.

Sample size	In this study, the HPLC and LC-MS analysis were determined with the sample of n=3.
Data exclusions	No data were excluded in this study.
Replication	All experiments were repeated independently and showed similar results. Number of repeats is given in the figure legends.
Randomization	In this study, the data represented are the biochemical assay on enzyme function, which require a rational approach to data collection and analysis; therefore, randomization is not applicable to the experimental set up.
Blinding	This issue is not related to our study, because it is an enzymatic study and appropriate control experiments were included.

Reporting for specific materials, systems and methods

We require information from authors about some types of materials, experimental systems and methods used in many studies. Here, indicate whether each material, system or method listed is relevant to your study. If you are not sure if a list item applies to your research, read the appropriate section before selecting a response.

Materials & experimental systems

n/a	Involved in the study
☒	☐ Antibodies
☒	☐ Eukaryotic cell lines
☒	☐ Palaeontology and archaeology
☒	☐ Animals and other organisms
☒	☐ Human research participants
☒	☐ Clinical data
☒	☐ Dual use research of concern

Methods

n/a	Involved in the study
☒	☐ ChIP-seq
☒	☐ Flow cytometry
☒	☐ MRI-based neuroimaging
